# Environmental determinants and demographic influences on global urban microbiomes, antimicrobial resistance and pathogenicity

**DOI:** 10.1038/s41522-023-00459-4

**Published:** 2023-12-07

**Authors:** Yang Chen, Xi Fu, Zheyuan Ou, Jiang Li, Simiao Lin, Yaoxuan Wu, Xuwei Wang, Yiqun Deng, Yu Sun

**Affiliations:** 1 Guangdong Provincial Key Laboratory of Protein Function and Regulation in Agricultural Organisms, College of Life Sciences, South China Agricultural University, Guangzhou, P. R. China; 2 Guangdong Provincial Engineering Research Center of Public Health Detection and Assessment, School of Public Health, Guangdong Pharmaceutical University, 510006 Guangzhou, P. R. China

**Keywords:** Environmental microbiology, Microbial communities, Antimicrobials

## Abstract

Urban microbiome plays crucial roles in human health and are related to various diseases. The MetaSUB Consortium has conducted the most comprehensive global survey of urban microbiomes to date, profiling microbial taxa/functional genes across 60 cities worldwide. However, the influence of environmental/demographic factors on urban microbiome remains to be elucidated. We collected 35 environmental and demographic characteristics to examine their effects on global urban microbiome diversity/composition by PERMANOVA and regression models. PM_10_ concentration was the primary determinant factor positively associated with microbial α-diversity (observed species: *p* = 0.004, β = 1.66, *R*^2^ = 0.46; Fisher’s alpha: *p* = 0.005, β = 0.68, *R*^2^ = 0.43), whereas GDP per capita was negatively associated (observed species: *p* = 0.046, β = −0.70, *R*^2^ = 0.10; Fisher’s alpha: *p* = 0.004, β = −0.34, *R*^2^ = 0.22). The β-diversity of urban microbiome was shaped by seven environmental characteristics, including Köppen climate type, vegetation type, greenness fraction, soil type, PM_2.5_ concentration, annual average precipitation and temperature (PERMANOVA, *p* < 0.001, *R*^2^ = 0.01–0.06), cumulatively accounted for 20.3% of the microbial community variance. Canonical correspondence analysis (CCA) identified microbial species most strongly associated with environmental characteristic variation. Cities in East Asia with higher precipitation showed an increased abundance of *Corynebacterium metruchotii*, and cities in America with a higher greenness fraction exhibited a higher abundance of *Corynebacterium casei*. The prevalence of antimicrobial resistance (AMR) genes were negatively associated with GDP per capita and positively associated with solar radiation (*p* < 0.005). Total pathogens prevalence was positively associated with urban population and negatively associated with average temperature in June (*p* < 0.05). Our study presents the first comprehensive analysis of the influence of environmental/demographic characteristics on global urban microbiome. Our findings indicate that managing air quality and urban greenness is essential for regulating urban microbial diversity and composition. Meanwhile, socio-economic considerations, particularly reducing antibiotic usage in regions with lower GDP, are paramount in curbing the spread of antimicrobial resistance in urban environments.

## Introduction

Urban microbiome research refers to the study of microorganisms (bacteria, viruses, fungi, and other microbes) in urban environments. The goal of this research area is to understand the complex relationships between the urban environment, human health, and biodiversity, and to catalog potential pathogens and protective microorganisms that exist in urban environments. The importance of studying the urban microbiome for human health cannot be understated. Firstly, the examination of the microbiome composition and diversity in domestic, healthcare, and public transportation environments has uncovered the prevalence, transmission mechanisms, and pathogenic microorganisms present in these settings^1–5^. Secondly, the urban microbiome studies also shed light on the potential risk and protective microorganisms associated with chronic and allergic diseases, such as asthma, rhinitis, allergies, and sick-building syndrom^6–8^. Third, urban microbiome research has critical implications for the growing issue of antimicrobial resistance (AMR). It is particularly important given the escalating global consumption of antimicrobial drugs, which increased by 65% from 2000 to 2015^9^. This surge in consumption has contributed to the rise of drug-resistant pathogens. If this trend continues, it could lead to 10 million deaths annually by 2050^10^. As such, in-depth exploration and understanding of the urban microbiome become crucial for devising strategies to curb the rise of AMR and protect human health.

The largest urban microbiome research to date is the MetaSUB consortium, an international collaboration of scientists and researchers from diverse backgrounds and disciplines focused on sampling urban public facilities. Between 2015 and 2017, the consortium collected 4,782 metagenomic samples from 60 cities worldwide and produced the first comprehensive catalog of global urban microbial ecosystems^11^. This project has significant implications for public health, as it provides officials with tools to assess risk microbial agents, given that urban microbiomes are closely related to many diseases. However, despite the exhaustive survey of urban microbiomes and microbial functional genes conducted by the MetaSUB project, it made limited efforts in terms of revealing the effect of environmental characteristics on urban microbiomes. The study considered only a few factors, including population, elevation, proximity to the coast, population density, and region, which cannot systematically and comprehensively reveal the influence of environmental/demographic characteristics on the diversity and composition of urban microbiomes.

There are numerous environmental and demographic factors that may impact the diversity, composition, antimicrobial resistance, and presence of pathogenic microorganisms in the urban microbiome. For instance, air pollutants, including particulate matter (PM), CO, SO_2_, NO_2_ and O_3_, have been shown to affect the composition of air-borne microorganisms^12,13^. The particulate matter can adsorb microorganisms, and microorganisms can interact with PM in the ambient air^12–14^. Meanwhile, the concentrations of SO_2_, NO_2_, and O_3_ can interfere with the growth and spread of bacteria^13^. Furthermore, meteorological conditions, such as temperature, annual precipitation, and relative humidity, also impact the composition, structure, and richness of the microbiome^15–20^. Additionally, vegetation and soil properties have been found to affect the variation of microbiome communities in urban environments^21^. However, a significant limitation of these studies is that they have largely been conducted in a limited number of urban regions or cities. As such, their findings often lack the broad applicability necessary to encapsulate the global patterns of how specific environmental and demographic factors influence urban microbiomes. A global-scale dataset offers a unique opportunity to address this limitation, enabling us to understand the overarching environmental and demographic factors affecting urban microbiomes across diverse geographic and cultural contexts.

This research aims to investigate the impact of various environmental and demographic factors on the diversity and structure of the urban microbiome at a global level using data from the MetaSUB project. The study analyzed 35 different environmental and demographic characteristics from public databases, including air pollutants, soil characteristics, precipitation, radiation, humidity, annual average temperature, vegetation, population density, and Gross Domestic Product (GDP). To assess the effects of these environmental factors on microbial diversity, composition, antimicrobial resistance, and pathogens, permutation and regression models were utilized. Studying the impact of environmental characteristics on the urban microbiome is important for improving our understanding of the complex relationships between urban environments, human health, and biodiversity, and for designing more sustainable and resilient cities.

## Results

### Global urban microbiome composition and diversity

The MetaSUB project reported the global urban microbiome across 60 cities in 33 countries. Predominantly, samples were obtained from high-contact surfaces in metro and transit stations, including ticket kiosks, turnstiles, railings, and seats or benches. Other locations included residences, seashores, schools, hospitals, and parks. To maintain sample source consistency, only samples from transportation systems were retained for further analysis. Following this refinement, it became evident that certain cities contributed a few samples, thus making them unsuitable for in-depth analysis. Accordingly, we excluded cities contributing fewer than 14 samples from transportation systems, leading to a refined set of 32 cities for further exploration. Our re-analysis of the global urban microbiome data from MetaSUB encompassed a comparative study of microbial alpha diversity, and the evaluation of the prevalence and abundance of antibiotic-resistance genes and potential pathogens.

**Fig. 1:**
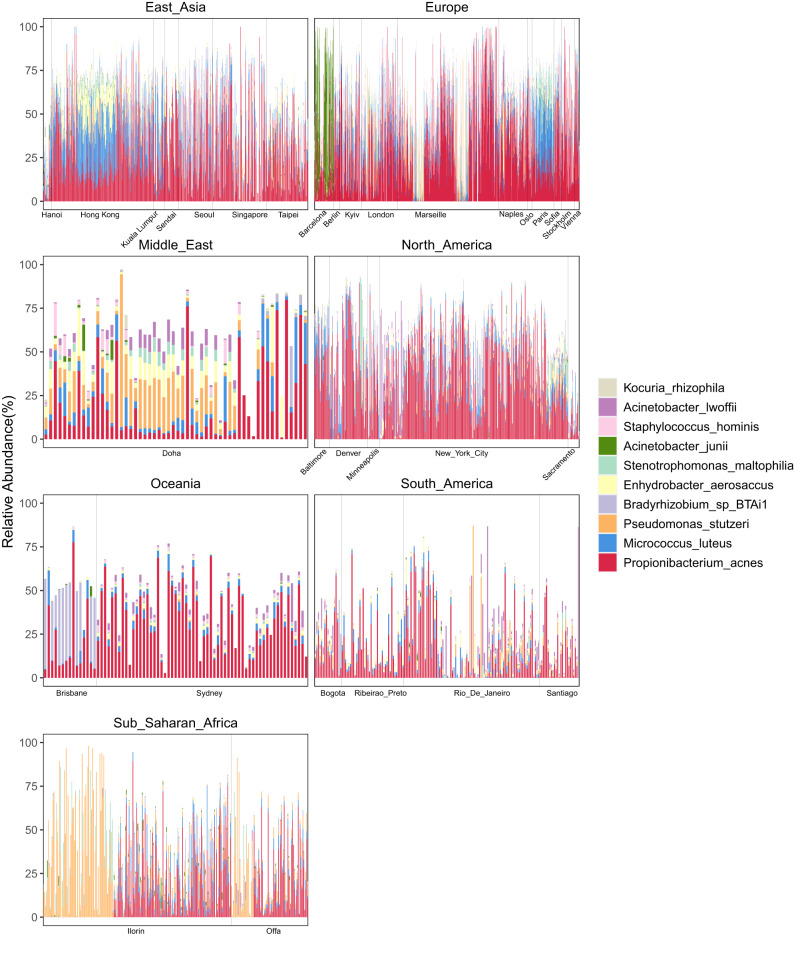
Top ten species in the global urban microbiome by continent. This figure illustrates the distribution of the top ten species across all samples in the global urban microbiome, with each panel representing a different continent.

The relative abundance of the top 10 species (Fig. 1) and genera (Supplementary Fig. 1) was calculated across all samples (Fig. 1, Supplementary Fig. 1). *Propionibacterium acnes* was ubiquitous across all surveyed cities, present in global urban regions with an average abundance of 27.7%. However, other species demonstrated stronger geographic patterns. For instance, *Pseudomonas stutzeri* was more commonly found in Ilorin, Doha and Offa (average relative abundance of 18.2%, 13.6% and 10.5%). *Bradyrhizobium* sp BTAi1 was most prominent in Brisbane (29.0%), while *Acinetobacter junii* largely dominated Barcelona (38.7%) (Fig. 1). Similarly, distinct geographic patterns were also observed at the genus level. For instance, the *Pseudomonas* genus had high abundance in Ilorin (24.8%), Doha (21.3%), and Offa (17.0%), while the *Micrococcus* genus was more abundant in Oslo (25.2%).

**Fig. 2:**
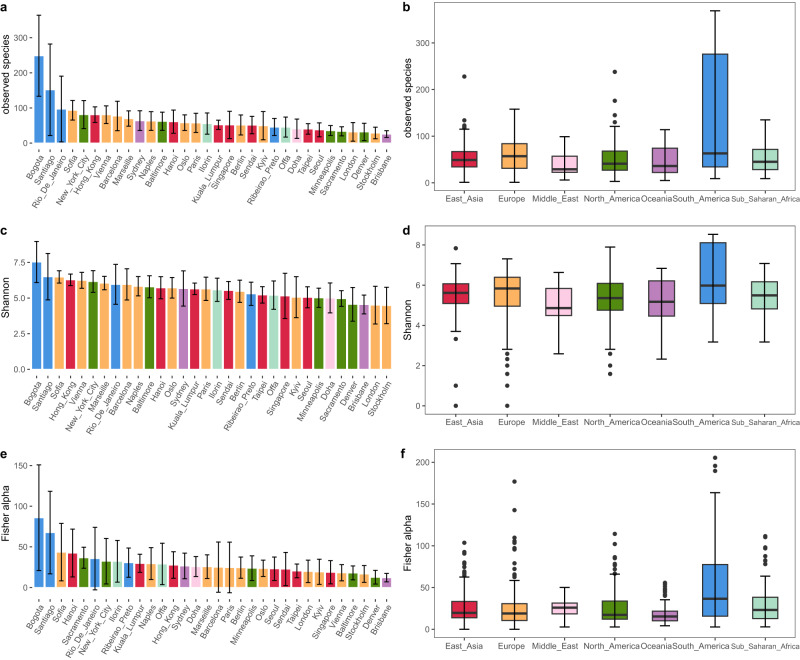
Alpha diversity of global urban microbial communities. The alpha diversity was assessed using **a**, **b** the number of observed species, **c**, **d** the Shannon index, and **e**, **f** Fisher’s alpha diversity. In both bar charts and boxplots, samples were categoried and color-coded according to continents. The median is represented by the center line in each box, while the bounds of the box and the whiskers represent the interquartile range (IQR) and 1.5 times the IQR, respectively.

The disparity in the number of samples per city could affect the estimation of alpha and beta diversity. To mitigate this, we standardized the sample size to 55 for each city, a figure chosen as it was the median sample number in the study. We achieved this standardization by employing a drawing without replacement statistical technique until the alpha diversity distribution of the subsample from each city was not significantly different from the total sample (Two Sample Kolmogorov–Smirnov test, average *p*-value: 0.89, Supplementary Table 5). This approach allowed for a balanced comparative analysis across cities. Using this standardized sample set, we found the Shannon index, number of observed species, and Fisher’s alpha diversity were highest in Bogota and Santiago in South America compared to other cities (Fig. 2). Alpha diversities were significantly higher in South American samples compared to other continents (*p* < 0.001, Mann–Whitney test; Supplementary Table 6), demonstrating significant continental differences in microbial diversities.

**Fig. 3:**
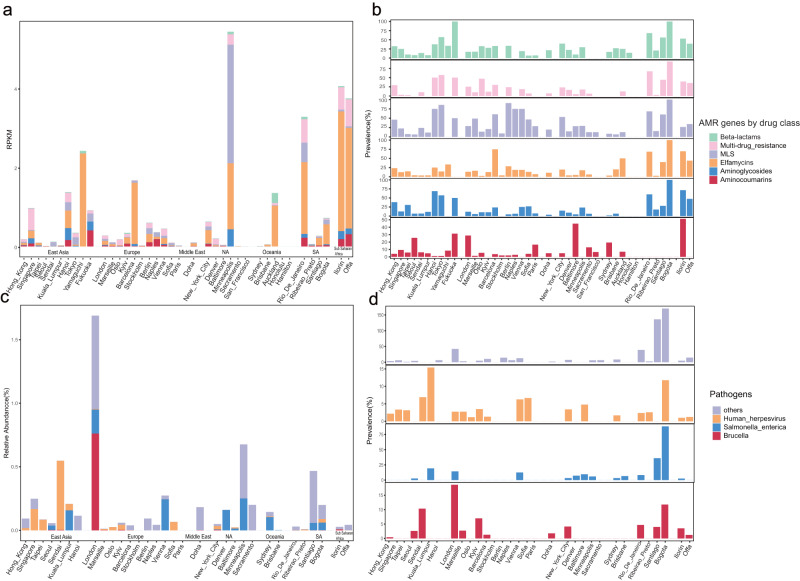
The distribution of abundance and prevalence of AMR genes by drug class, and abundance of pathogens. **a** The relative abundance using RPKM for 6 major classes of antibiotic resistance (AMR) genes. **b** The prevalence for 6 classes of AMR genes. **c** The relative abundance of the three most prevalent pathogens across the samples. **d** The prevalence of these three major pathogens in the samples.

**Fig. 4:**
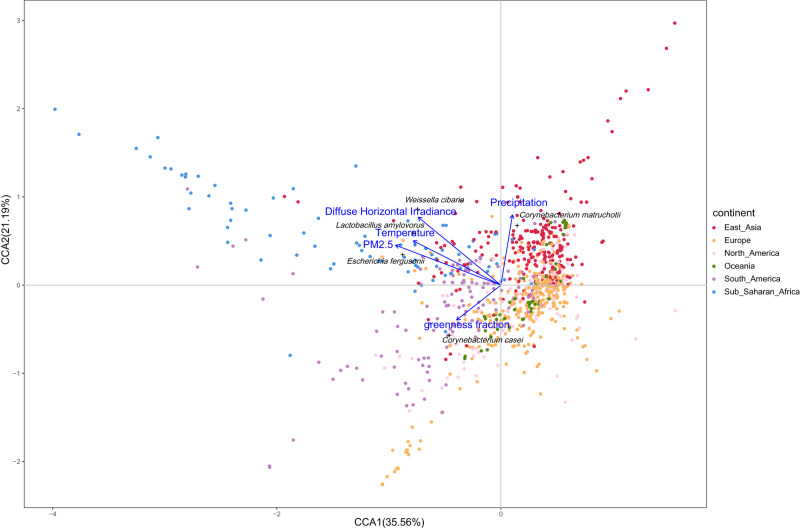
Canonical correspondence analysis (CCA) of global urban microbiome and environmental characteristics. This figure presents CCA plots illustrating the influence of significant environmental characteristics (depicted as blue arrows) on the composition of the global urban microbiome. Samples are categorized and color-coded according to continent. The microbial species most strongly correlated with these environmental characteristics are highlighted. The final multivariate model is statistically significant (*p* < 0.001, permutation test), and only those environmental characteristics that are significant in the final model are shown. The lengths of the lines representing environmental characteristics indicate the strength and direction of the relationship between these characteristics and the microbiome data.

In this study, we focused our analysis on the top six classes of antimicrobial resistance (AMR) genes (those with a prevalence > 20%). These included AMR genes related to resistance against MLS (Macrolide/Lincosamide/Streptogramin), Beta-lactams, Multi-drug, Aminoglycosides, Elfamycins, and Aminocoumarins (Figs. 3a, 4b). AMR genes responsible for resistance to MLS were observed in almost all cities, with the highest detection rates in Berlin (90.48%) and Bogota (100%). However, despite the high prevalence, the average abundance of MLS resistance genes in Berlin and Bogota was low (Average RPKM = 0.12 and 0.15), whereas it was highest in Minneapolis (RPKM = 2.99). Multi-drug resistance genes were commonly detected in Bogota (93.75%), but their average abundance (RPKM = 0.029) was much lower compared to Offa (RPKM = 0.68) and Rio de Janeiro (RPKM = 0.59). AMR genes conferring resistance to Beta-lactams were widespread in cities such as Bogota (100%), Fukuoka (100%), and Rio de Janeiro (100%), but their relative abundance (RPKM = 0.015, 0.005, 0.06, respectively) was generally low on a global scale. Overall, the abundance and prevalence of major AMR genes varied among cities. While Bogota exhibited the highest prevalence (>90%) of detected AMR genes, the average abundance of these genes was lower in Bogota compared to other cities like Barcelona and Kuala Lumpur.

Only three pathogens that were detected in more than 3% of samples were kept for further analysis, including *Salmonella enterica*, *Brucella*, and Human Herpesvirus (Figs. 3c, 4d). While *Salmonella enterica* was most prevalent in Bogota (88.2%) and Santiago (36%), its relative abundance was highest in Minneapolis (0.25%), Vienna (0.25%), and London (0.19%). Similarly, *Brucella* was prevalent in London (18.6%), Bogota (11.7%), and Sendai (10.3%), and its relative abundance was highest in London (0.76%). Finally, Human Herpesvirus showed high prevalence (6.9%) and abundance (0.54%) in Sendai, which may pose a potential threat to the city. The average abundance of these pathogens combined was highest in London (1.7%), followed by Minneapolis (0.7%) and Sendai (0.5%). The abundance and prevalence of all pathogens defined based on NIAID can be found in Supplementary Tables 7, 8.

### Variation of environmental and demographic characteristics

The demographic and environmental characteristics of the sampled cities are presented in Table 1, Supplementary Table 1, Supplementary Tables 3, 4. The highest GDP per capita was recorded in Switzerland at 83,073 USD, while the lowest was in Nigeria at 2176 USD. The median GDP per capita was 41,048 USD, with a range of 20,619-55,628 USD (Q1-Q3). The median and Q1–Q3 of the total population and population density of the cities were 1.62 million (0.61 million, 7.23 million) and 4310 people/km^2^ (2236 people/km^2^, 7282 people/km^2^), respectively. Shanghai had the highest population (24.2 million) and Paris had the highest population density (21,000 people/km^2^) among the sampled cities.

**Table 1:** The unit, median, Q1, and Q3 of environmental and demographic characteristics in this study.

	Environment factor	Unit	Median (Q1, Q3)/category
Air pollutants	PM_2.5_ concentration	μg/m^3^	11.71 (7.67,16.42)
	PM_10_ concentration	μg/m^3^	19.14 (16.15,28.11)
	O_3_ concentration	ppbv	41.58 (34.79,45.6)
	NO_2_ concentration	μg/m^3^	29.24 (19.2,39.62)
	CO concentration	ppbv	136.91 (132.56,138.74)
Soil characteristics	Soil pH	*10^−1^	63 (56,69.5)
	Soil organic carbon density	t ha^−1^	12 (7,16)
	Soil organic carbon	% of weight, 0.01	185.64 (131.32,346.47)
	Total carbon	% of weight, 0.01	247.41 (175.29,306.84)
	Soil type		10 categories
	Total phosphorus	% of weight, 0.0001	531.57 (339.81,607.94)
	Soil moisture	kg/m^2^	31.4 (29.15,34.47)
	Soil temperature	K	288.13 (283.15,293.68)
Precipitation	Precipitation	mm	945.44 (694.93,1279.49)
	Precipitation concentration index		10.96 (9.81,12.65)
	Standard deviation of monthly precipitation		39.06 (27.88,75.45)
Irradiation	UV index	1(0 ~ 15)	4.73 (2.62,7.26)
	Diffuse Horizontal Irradiance	W/m^2^	3.87 (2.97,4.7)
Humidity	Average rainy day per month	days	12.98 (10.42,15.46)
	Vapor pressure	Hecta-Pascals (x10)	12.38 (10.19,17.59)
	Rainy days in June	days	14.55 (11.88,17.27)
	RH night	%	78.8 (76.19,82.6)
	RH day	%	62.39 (54.69,68.09)
	RH	%	70.96 (65.95,74.97)
Temperature	Annual average temperature	°C	14.52 (10.22,18.75)
	Average temperature in June	°C	21.70 (17.85,25.45)
	Urban-rural temperature difference during day time	°C	2.44 (1.89,4.22)
	Urban-rural temperature difference during night time	°C	0.93 (0.46,1.37)
Demographic data	City population	hundred thousand	16.25 (6.05,72.62)
	Total antibiotic consumption	DDD/1,000/day	18.49 (12.075,25)
	GDP per capita	housand US dollar	41.05 (20.62,55.63)
	Population density	/km^2^	4310 (2236,7282)
Other characteristics	Adjacent to coast		Yes/no
	Elevation	meters	96 (11.75,414.75)
	Climate type		equatorial,arid,warm,temperate,snow
	Vegetation type		14 categories
	Greenness fraction	%	0.92 (0.89,0.94)
	Fire carbon emissions	g C m^−2^ month^−1^	0 (0,2.77)

The annual average concentration data for 5 air pollutants (PM_2.5_, PM_10_, O_3_, NO_2_, and CO) are presented in Table 1. Twelve cities had an annual average PM_2.5_ concentration exceeding 20 µg/m^3^, with the highest concentration recorded in Doha at 88.77 µg/m^3^. Seoul had high annual average concentrations of PM_10_, O_3_, NO, and CO at 48 µg/m^3^, 47.98 ppbv, 58.28 µg/m3, and 143.86 ppbv, respectively. The concentration of O_3_ was relatively high in Japan (median: 41.58; Q1-Q3: 34.79, 45.6), particularly in Tokyo (48.52 ppbv), Sendai (48.22 ppbv), Yamaguchi (47.56 ppbv), and Fukuoka (47.16 ppbv).

The annual average precipitation in Asia was found to be higher than in other regions (*p* < 0.05). The cities of Hong Kong, Fukuoka, Singapore, and Taipei experienced annual precipitation levels exceeding 2000 mm, compared to the global average of 945.44 mm. The Precipitation Concentration Index (PCI) is a metric for assessing the variation in temporal precipitation distribution. A higher PCI value indicates a greater variation in seasonal precipitation. Doha had the highest PCI value in this study (PCI = 54.35), followed by Santiago, San Francisco, and Sacramento with PCI values of 26.56, 19.36, and 18.87, respectively. The median annual average temperature among the cities was 14.5 °C. Doha had the highest annual average temperature at 29.13 °C, while Fairbanks had the lowest at 0.24 °C. Doha had the lowest greenness fraction (21.1%) among all cities, while Hamilton had the highest (97.5%). The highest daytime temperature difference between the urban area and buffer area was recorded in Mexico City (7.9 °C), while the lowest was in Fairbanks (0.13 °C) and Doha (−0.31 °C).

Since the MetaSUB samples were collected in June, we also collected environmental factors in June specifically. The number of rainy days was the lowest in Doha (0 days) and the highest in Zurich (27 days), while the highest temperature in June was in Doha (37.5 °C) and the lowest was in Santiago (8.3 °C). The soil physical and chemical properties were also collected, including soil type (10 soil types), total soil phosphorus (median: 5.3%, Q1, Q3: 3.4%, 6.1%), soil pH (median: 6.3; Q1, Q3: 5.6, 7.0), etc. Birmingham had the most acidic soil (pH=4.8), while Taipei had the highest soil pH (8.0).

Diffuse Horizontal Irradiance (DHI) is the radiation scattered from the atmosphere to the surface of the earth. UV index is the strength of sunburn-producing ultraviolet (UV) radiation. GHI and UV index are important factors for assessing the potential of solar energy. Among the cities evaluated, Ilorin and Offa had the highest DHI values of 2.94 W/m^2^ and 2.93 W/m^2^, respectively. These cities also had the highest GHI values, with Ilorin at 4.99 W/m^2^ and Offa at 4.76 W/m^2^. Fairbanks had the highest UV index at 12.63, followed by Ilorin at 10.63 and Offa at 10.31.

In 2016, France (29.2 DDD/1000/day) had the highest antibiotic consumption among 25 countries (Supplementary Tables 3, 4), followed by Spain (28.1 DDD/1000/day), Vietnam (27.1 DDD/1000/day), and Australia (25.7 DDD/1000/day). Nigeria had the lowest antibiotic consumption (7.5 DDD/1000/day), followed by Colombia (8.8 DDD/1000/day), Hong Kong (8.8 DDD/1000/day), Malaysia (9.9 DDD/1000/day), Sweden (11.6 DDD/1,000/day), and Singapore (12.8 DDD/1000/day).

### Environmental and demographic characteristics associated with urban microbiome diversity

The association between environmental and demographic characteristics and urban microbiome alpha diversity was evaluated using bivariate regression analysis (Table 2, Supplementary Tables 9, 10, 11). The most significant factor in determining microbial number of observed species and Fisher’s alpha was PM_10_ concentration (*p* = 0.004, β = 1.66, *R*^2^ = 0.46; *p* = 0.005, β = 0.68, *R*^2^ = 0.43), followed by GDP per capita (p = 0.046, β = −0.7, R^2^ = 0.10; *p* = 0.004, β = −0.34, *R*^2^ = 0.22). High PM_10_ concentration correlated with increased urban microbiome diversity. For example, Santiago, which had the highest PM_10_ concentration (66.83 μg/m^3^), ranked second in terms of number of observed species (151.78) and Fisher’s alpha (67.63) among all the cities. There was an inverse relationship between the number of observed species and Fisher’s alpha in global urban microbiomes and GDP per capita (Table 2), suggesting that microbial richness was lower in wealthier cities. To summarize, both environmental and demographic characteristics influenced the number of observed species and Fisher’s alpha in the urban microbiome, with PM_10_ concentration exerting the most significant effect. However, our research did not indicate a significant impact of environmental factors on the Shannon index.

**Table 2:** Bivariate associations between environmental characteristics and the number of observed species and Fisher’s alpha of urban microbiome (alpha diversity).

	Coef.	*P*>|t|	95%CI		Adj.*R*-squared
Number of observed species					
PM_10_	1.665	0.004	0.629	2.700	0.464
GDP per capita	−0.702	0.046	−1.390	−0.014	0.1011
Fisher’s alpha					
PM_10_	0.683	0.005	0.249	1.118	0.430
GDP per capita	−0.338	0.004	−0.559	−0.117	0.221

The associations were calculated by linear regression. The associations with *p*-value < 0.05 were presented in this table.

The association of environmental and demographic characteristics with urban microbiome beta diversity was examined using multivariate PERMANOVA analysis based on the Bray–Curtis distance matrix. The global urban microbial community composition was influenced by Köppen climate type (*p* < 0.001, *R*^2^ = 0.053), vegetation type (*p* < 0.001, *R*^2^ = 0.037), soil type (*p* < 0.001, *R*^2^ = 0.029), PM_2.5_ concentration (*p* < 0.001, *R*^2^ = 0.028), precipitation (*p* < 0.001, *R*^2^ = 0.021), greenness fraction (*p* < 0.001, *R*^2^ = 0.012), annual average temperature (*p* < 0.001, *R*^2^ = 0.011) (Table 3). In total, 20.3% of the variation in urban microbial communities was explained by these environmental characteristics. Environmental characteristics but no demographic characteristics were associated with urban microbiome beta diversity. Also, unlike a single dominant factor in microbial alpha diversity (PM_10_), urban microbiome beta diversity was shaped by a combination of ten environmental characteristics.

**Table 3:** Multivariate associations between environmental characteristics and community variation of urban microbiome (beta diversity).

	*F*	Pr(>*F*)	*R*^2^
Köppen climate type	16.885	<0.001	0.053
Vegetation type	13.267	<0.001	0.037
Soil type	13.094	<0.001	0.029
PM_2.5_	88.845	<0.001	0.028
Precipitation	65.764	<0.001	0.021
Greenness fraction	38.555	<0.001	0.012
Annual average temperature	33.838	<0.001	0.011

The associations were conducted by permutational multivariate analysis of variance (PERMANOVA) based on the Bray–Curtis distances. The statistical significance and F-statistics were calculated, using 999 permutations.

The relationship between environmental factors and urban microbiome beta diversity was further verified using canonical correspondence analysis (CCA) ordination analysis (Fig. 4). The results showed that a similar set of environmental factors, including DHI, annual average temperature, PM_2.5_ concentration, greenness fraction, precipitation, were associated with the urban microbiome’s beta diversity. CCA analysis also identified the microbial species most strongly associated with these environmental characteristics. For instance, cities in East Asia with higher precipitation showed an increased abundance of *Corynebacterium metruchotii* (Fig. 4). Meanwhile, cities in North and South America with a higher greenness fraction exhibited a higher abundance of *Corynebacterium casei*. Furthermore, an increase in PM_2.5_ concentration, temperature, and DHI was respectively linked to a higher abundance of *Escherichia fergusonii*, *Lactobacillus amylovorus*, and *Weissella cibaria*.

### Demographic and environmental characteristics affect AMR genes and pathogens

The abundance and prevalence of global urban microbial AMR genes were found to be affected by demographic characteristics. GDP per capita was significantly negatively correlated with the prevalence of resistance genes for Aminoglycosides (β = −0.0069, *p* = 0.0002), Elfamycins (β = −0.0054, *p* = 0.0024), total AMR (β = −0.064, *p* = 0.0015), and the abundance of resistance genes for Aminocoumarins (β = −0.0018, *p* = 0.0045; Table 4; Supplementary Fig. 2; Supplementary Table 12).

**Table 4:** Bivariate associations between environmental characteristics and the relative abundance and prevalence of AMR genes.

AMR genes characteristics	Environmental characteristics	Coefficient	95%CI		P>|t|	AdjR-squared
Aminocoumarins abundance	DHI	0.0853	0.0304	0.1402	0.0035	0.2262
	GDP per Capita	−0.0018	−0.0030	−0.0006	0.0045	0.2208
	Vapor pressure	0.0057	0.0019	0.0095	0.0045	0.2144
Aminoglycosides prevalence	GDP per Capita	−0.0069	−0.0102	−0.0036	0.0002	0.3723
	DHI	0.2986	0.1407	0.4565	0.0006	0.3098
	UV index	0.0472	0.0219	0.0725	0.0006	0.3037
Beta-lactams prevalence	UV index	0.0388	0.0153	0.0622	0.0021	0.2508
Elfamycins prevalence	GDP per Capita	−0.0054	−0.0088	−0.0021	0.0024	0.2508
Multi-drug resistance abundance	O_3_ concentration	−0.0124	−0.0205	−0.0042	0.0047	0.2788
	Vapor pressure	0.0162	0.0070	0.0254	0.0012	0.2776
	DHI	0.2206	0.0837	0.3575	0.0026	0.2406
Total AMR Prevalence	GDP per Capita	−0.0638	−0.1011	−0.0265	0.0015	0.2730
	UV index	0.4376	0.1525	0.7227	0.0038	0.2216

The associations were calculated by linear regression model, and only associations with *p*-value < 0.005 were presented in this table.

Two radiation-related environmental characteristics were also associated with microbial AMR genes. The UV index was significantly positively associated with the prevalence of resistance genes for Aminoglycosides (β = 0.0472, *p* = 0.0006), Beta-lactams (β = 0.0388, *p* = 0.0021), total AMR (β = 0.4376, *p* = 0.0038), while DHI was significantly positively associated with the prevalence of Aminoglycosides resistance genes (β = 0.2986, *p* = 0.0006), and the abundance of resistance genes for Aminocoumarins (β = 0.0853, *p* = 0.0035) and Multi-drug resistance (β = 0.2206, *p* = 0.0026; Table 4; Supplementary Figs. 3, 4; Supplementary Table 12). In addition, the O_3_ concentration was negatively associated related to the abundance of Multi-drug resistance genes (β = −0.0124, *p* = 0.0047; Table 4; Supplementary Fig. 5; Supplementary Table 10). The vapor pressure (Supplementary Fig. 6) were significantly positively associated with the abundance of resistance genes for Aminocoumarins (β = 0.0057, *p* = 0.0045) and Multi-drug resistance (β = 0.0162, *p* = 0.0012). Interestingly, antibiotic consumption was not associated with any of the AMR genes.

Associations between environmental and demographic characteristics and the abundance of human pathogens were also analyzed (Table 5 and Supplementary Table 13). Human pathogens were defined by NIAID, which classifies over 70 emerging diseases and pathogens that significantly threaten public health. In the MetaSUB shotgun dataset, most of these pathogens were absent or in very low abundance. Analysis was conducted only on *Brucella* species (*B. ovis* and *B. pinnipedialis*), *Salmonella enterica*, Human Herpesvirus, and a combination of these three pathogens. The prevalence of combined three major pathogens was positively associated with urban population (β = 0.0071, *p* = 0.0309) and negatively associated with temperature in June (β = −0.0365, *p* = 0.0273). Additionally, compared with non-coastal cities, coastal cities have a significantly higher abundance of *Salmonella enterica* (β = −0.0006, *p* = 0.0238).

**Table 5:** Bivariate associations between environmental characteristics and the abundance of pathogens.

y	x	Coef.	*P*>|*t*|	95%CI	95%CI	Adj *R*-squared
*Salmonella enterica* abundance	adjacent to coast	−0.0006	0.0238	−0.0011	−0.0001	0.131
Combined pathogen prevenlence	average temperature in June	−0.0365	0.0273	−0.0686	−0.0044	0.1369
	city population	0.0071	0.0309	0.0007	0.0135	0.1255

This study analyzed only three pathogens that were detected in more than 3% of all samples, including *Brucella* species (*B. ovis* and *B. pinnipedialis*), *Salmonella enterica*, and Human Herpesvirus. Alongside individual regression analysis for each pathogen, we also conducted a combined analysis for all these three pathogens. This table only presents associations with a p-value less than 0.05.

## Discussion

The strengths of this study include being the first to report on the comprehensive associations between environmental and demographic characteristics and urban microbiome data at a global scale. Thirty-four environmental and demographic characteristics were obtained from public databases, providing insight into the impact of these factors on urban microorganisms and antimicrobial resistance. In addition, the urban microbiome data was analyzed from multiple perspectives, including alpha diversity (Shannon index and observed species), beta diversity (PERMANOVA and CCA). Features of global urban microbiome data were better understood by examining the different levels of diversity.

However, there are also some limitations to consider. Firstly, the use of relative abundances in metagenomics studies can be a limitation. While relative abundance provides important insights into the distribution and prevalence of microbial species, it does not convey quantitative information regarding the absolute load of these entities. This is especially crucial when investigating pathogens and AMR genes^22,23^. Secondly, the accurate characterization of pathogens is challenging by the metagenomics data. The pathogens are characterized at the species level in this study. However, it is generally accepted that pathogens can be more accurately characterized at the strain level rather than just at the species level. Different strains of a species can have different genetic makeup, which can result in varying virulence or pathogenicity. For example, strains of a species may contain specific pathogenicity islands, which can contribute to the pathogenicity of the strain. However, the short-read shotgun metagenomics data were assembled in fragmented scaffolds, making it difficult to determine whether a particular species contains specific pathogenicity islands or not. In addition, it’s crucial to understand that the detection of DNA sequences of a pathogen doesn’t necessarily imply that the pathogen is live or capable of causing infection. The presence of DNA could result from dead or non-viable organisms, or free DNA persisting in the environment. Therefore, while metagenomic sequencing is a powerful tool for pathogen identification, additional laboratory tests are necessary to confirm the viability and potential infectiousness of detected pathogens. Third, our study implemented an associative analysis between environmental characteristics and the urban microbiome. It is crucial to note that association does not imply causation. Although we found a correlation between certain characteristics and microbial diversity, this does not definitively conclude that these characteristics directly influence microbial diversity. It’s conceivable that both may be influenced by an unobserved variable.

In this study, the concentration of PM_10_ has a significant positive impact on the alpha diversity of the urban microbiome, which accounts for 46% of the variation in alpha diversity. The size of non-biological particles in the air has been shown to affect the diversity and abundance of airborne microorganisms. A study in atmospheric aerosols has shown that bacterial diversity and relative abundance of coarse particles (diameter 2.5μm to 10μm) are 80% higher compared to fine particles (<2.5μm)^24^. Several other studies have also concluded that coarse particles play a more significant role in determining fungal and bacterial alpha diversity compared to fine particles, with the alpha diversity of fungi and bacteria increasing as the size of particulate matter increases from PM_2.5_ to PM_10_^25–27^. Moreover, it has been observed that the abundance of potential bacterial and fungal pathogens increases in correlation with PM diameter and pollution levels^25,28^, which may cause various diseases, such as cardiovascular and respiratory diseases^29^.

It’s important to note that the MetaSUB samples predominantly originate from indoor locations within transportation systems, whereas many of the examined environmental factors, including PM10, are outdoor variables. The influence of outdoor environmental characteristics on indoor microbiomes has been extensively documented in the literature^6,30,31^. These outdoor environmental features, such as particulate matter and pollutants, can impact the indoor microbiome through airflows and surface contacts. Moreover, a substantial portion of indoor microorganisms are derived from the outdoor environment (over 70%)^32^, further amplifying the significant influence outdoor environmental characteristics can have on the indoor microbiome.

Our findings revealed a negative correlation between GDP per capita and the number of observed species in the urban microbiome. This relationship can be interpreted in several ways. Firstly, cities with higher GDP per capita typically exhibit higher levels of urban development and human activity, which could lead to a reduction in the overall microbial diversity due to the decreased availability of natural habitats and niche diversification for microbes^33^. Also, wealthier cities often implement more stringent sanitation and public health measures, which could reduce the prevalence and diversity of certain microbial species.

In this study, we found that vegetation type, greenness fraction, PM_2.5_, and soil type are important environmental characteristics influencing the structure of the urban microbiome. These characteristics affect both the air and soil microbiome, which constitute essential parts of the urban microbiome^8^. Vegetation serves as a key source of airborne microbial particles that contribute to microbial communities in built environments close to green areas^34^. Also, higher greenness fractions typically mean more vegetation and, therefore, increased sources for airborne microbes^35^. PM_2.5_, particulate matter smaller than 2.5 micrometers, can serve as a vehicle for transporting and spreading air microbes within and between urban environments^36,37^. The type and abundance of microbes associated with these particles can vary depending on the source and composition of the PM2.5, ultimately influencing the diversity of urban and indoor microbiomes. Soil types, distinguished by variations in composition, pH, organic content, moisture, and nutrient availability, can harbor unique microbial communities^38^. These communities can differ in their ability to survive and adapt to urban, built, or indoor environments, leading to distinct beta-diversities.

Moreover, it is not surprising that general climatic factors, such as annual average temperature, precipitation, and climate type, also affect the urban microbiome composition. Long-term experimental studies have demonstrated that temperature plays a vital role in modifying the community structure of soil microbiome. Temperature changes affect the growth rate and yield of specific microorganisms, and the effects are more pronounced in fungi as compared to bacteria^39–41^. Precipitation can also significantly influence the structure of urban microbiomes^42^. Rainfall and other forms of precipitation can facilitate the spread and redistribution of microbes, while also influencing environmental conditions, such as humidity, that can affect microbial survival and growth. Since temperature, humidity, and precipitation are fundamental aspects of climate, different climate types consequently become critical drivers of microbial diversity and community structure.

An intriguing pattern observed in this study pertains to the high detection rates but low relative abundances of some AMR genes in specific cities, such as gene resistence to MLS in Berlin and Bogota, gene resistence to Beta-lactams in Bogota, Fukuok, and Rio deJaneiro. The high detection rate of AMR genes, despite their low relative abundance, signifies the ubiquity of these resistance elements in urban environments. From a public health perspective, this observation is highly significant. Even with low relative abundance, these AMR genes can be readily transferred among microbial communities through horizontal gene transfer mechanisms, especially under the selective pressure imposed by antibiotic usage, contributing to the rapid spread and diversification of antimicrobial resistance^43,44^. Moreover, even if these AMR genes are present at low levels, bacteria carrying these genes can rapidly multiply and increase their abundance when they enter a host or find a conducive environment, exacerbating the challenge of managing infectious diseases. Hence, ongoing surveillance and mitigating measures are imperative to curtail the spread of these resistance genes in urban environments.

In our study, antibiotic consumption was not found to be directly associated with the abundance or prevalence of AMR genes. This divergence suggests the potential influence of other factors in shaping the distribution and prevalence of AMR genes in the global urban microbiome, which warrants further investigation. We discovered a notable correlation between GDP per capita and the prevalence of antibiotic-resistance genes in the global urban microbiome, where lower GDP per capita is associated with higher prevalence. The high prevalence of antimicrobial resistance in low- and middle-income countries can be attributed to several reasons, such as the easy availability of over-the-counter antibiotics, poor medical facilities, and water sanitation, the widespread use of antibiotics in animal husbandry, and a lack of related education^45^.

Besides GDP per capita, solar radiation (DHI and UV index) had a positive correlation with the prevalence and abundance of aminocoumarins, aminoglycoside, beta-lactams, multi-drug resistance, and total AMR genes. Solar radiation, including ultraviolet light, can cause DNA damage in microorganisms, leading to an increased rate of mutation and inactivation of bacteria and viruses^46,47^. However, no previous studies directly investigate their impact on AMR genes in an urban context.

We also found that the total pathogen prevalence, including *Brucella* and *Salmonella*, and Human herpesvirus, increased with city population, which reflects a potential public health threat to high-population cities. *Brucella* and *Salmonella* are food borne pathogens^48,49^. Higher population can result in increased demand for food, leading to increased production and transportation and storage of food, which in turn can increase the risk of contamination and spread. Likewise, Human herpesviruses, which are typically transmitted through close personal contact^50^. In densely populated cities, the increased rate of close personal interactions and communal living spaces may facilitate the transmission of these viruses. As such, urban centers with high population densities may serve as hotspots for the transmission of these human herpesviruses. Thus, our findings suggest a possible link between population and the prevalence of potential food-borne and contact-based pathogens, reflecting potential health risks in high-population urban areas.

Besides the city population, we found a negative association between the combined pathogen prevalence and the average temperature in June, suggesting that higher temperature in summer reduce the abundance of these pathogens. Microorganisms, including pathogens, can be sensitive to temperature. If the temperature range in June is outside the optimal growth range of a particular microorganism, it could affect the abundance of the organism. However, the temperature ranges for *Brucella* and *Salmonella*, and Human herpesvirus have not been reported by previous studies.

Our study presents the first comprehensive analysis of the influence of environmental and demographic characteristics on global urban microbiome diversity and composition. We also discovered relationships between certain characteristics and the prevalence of antimicrobial resistance genes and specific pathogens, such as *Salmonella enterica*. These findings have significant implications for urban planning and public health strategies. Recognizing the environmental and demographic factors that shape urban microbiomes may help inform the design of urban environments to optimize microbial community structures for improved health outcomes. It may also guide interventions in existing urban areas to mitigate disease risks and influence the spread of antimicrobial resistance. This work therefore establishes a foundation for continued exploration and understanding of the interplay between urban environments and their resident microbiomes, which is critical in our rapidly urbanizing world.

## Methods

### Global urban microbiome, AMR and pathogen data collection

The sequencing data and metadata files for the global urban microbial metagenomics were obtained from the MetaSUB consortium, short for Metagenomics and Metadesign of Subways and Urban Biomes (https://pngb.io/metasub-2021)^11^. In the MetaSUB project, a majority of samples were collected from the transportation systems (number of samples 2,674), with a smaller proportion derived from residence (381), seaside (178), school (142), hospital^45^ and park^51^. For transportation systems, samples were predominantly gathered from frequently touched surfaces in metro and transit stations across the globe, including elements like ticket kiosks, turnstiles, railings, and seating areas. In this study, to maintain consistency in sample sources, we retained only the samples from the transportation system for further analysis.

Samples were subjected to shotgun metagenomic sequencing using Illumina Next-Generation Sequencing (NGS) platforms. Each sample yielded between 5 to 7 million 125 bp paired-end reads. Detailed technical processing information about metagenomics assembly can be found in a previous publication^11^. To summarize, the MetaSUB project employed an assembly-based approach for microbiome data assembly and analysis. Specifically, the sequences were analyzed and assembled with metaSPAdes (v3.10.1) utilizing Bridges and Bridges-2^52^. The remaining contigs were mapped back to the reads using Bowtie2 to generate coverage metrics for each contig^51^. Contigs with coverage information were binned with MetaBAT2. The antimicrobial resistance genes (AMR) were profiled using MEGARes antibiotic resistance database^53^, which were calculated as reads per kilobase per million mapped reads (RPKM). Sample information and microbiome abundance tables were downloaded from the website as a part of the metadata file.

The prevalence of potentially pathogenic microbial taxa was calculated from the abundance table. Pathogenic taxa were defined based on the NIAID (National Institute of Allergy and Infectious Diseases) category A, B, and C priority pathogens (https://www.niaid.nih.gov/research/emerging-infectious-diseases-pathogens). Pathogenic taxa were defined at both the species level (*Salmonella enterica*) and genus or above levels (*Brucella* and Human Herpesvirus). The abundance of pathogenic *Brucella* was calculated as the total abundance of two *Brucella* pathogens, *Brucella ovis* and *Brucella pinnipedialis*. Similarly, Human Herpesvirus included Human Herpesvirus 1, Human Herpesvirus 4, and Human Herpesvirus 5. Only pathogens that were detected in more than 3% of all samples in MetaSUB database were kept for further analysis. The total abundance of pathogens was calculated from these pathogenic species.

### Environmental and demographic data collection

Over 70 environmental and demographic variables were collected from public databases for this study (Supplementary Table 1). These variables included air pollutants, soil data, climatic information, irradiation characteristics and demographic information. The environmental and demographic data were available in three formats: tabular, Esri grid (TIF/TIFF, NetCDF), and shapefile. The Esri grid data was extracted using ArcMap (version 10.8), a tool from the ArcGIS geospatial processing program. Variables in each city were extracted based on the latitude and longitude of the city, and a grid feature layer was created using the NetCDF data before sampling. The shapefile format data was converted to Esri grid format before extraction using the same process as described above. To eliminate highly correlated environmental and demographic characteristics, a spearman correlation analysis was performed using the psych package (version 2.2.5) in R (version 4.2.1).

We processed and standardized the environmental and demographic data to mitigate the impact of temporal fluctuations. The microbial samples analyzed in this study were collected in 2016 as part of the MetaSUB project, and the corresponding environmental and demographic data for the same year were also collated to ensure consistency. For many environmental characteristics, data were collected on a monthly and daily basis, and therefore, the annual averages for 2016 were calculated from these monthly and daily data. For instance, air pollutants such as PM_2.5_, PM_10_, O_3_, NO_2_, and CO were measured throughout the entire year and represented as yearly averages for 2016. Similarly, variables such as soil moisture, soil temperature, precipitation, and related parameters were also averaged on a monthly basis for the same year. For irradiation characteristics like Diffuse Horizontal Irradiance (DHI), the data represent daily averages calculated throughout the year 2016 (Supplementary Table 1).

We excluded characteristics showing a high degree of correlation, defined by a Spearman’s rank correlation coefficient exceeding 0.85, to prevent multicollinearity in subsequent regression analyses (refer to Supplementary Table 2). Consequently, we retained 35 environmental and demographic characteristics for regression and PERMANOVA analysis, detailed in Table 1 and Supplementary Tables 1, 3. To assess the quality of the collected demographic and environmental characteristics, we calculated the proportion of missing values for all collected attributes (Supplementary Table 1). Out of the 35 environmental and demographic characteristics, 24 had complete data for all 32 cities. Air pollution data had a high proportion of missing values, with PM_10_ (53.13% missing), NO_2_ (46.88%), CO (34.38%), and O_3_ (25.00%) leading the list. GDP per capita (3.13%), oil moisture (9.38%), soil temperature (9.38%), population density (9.38%), urban-rural temperature difference during daytime (21.88%), urban-rural temperature difference during nighttime (21.88%), and relative humidity (21.88%) had a lower proportion of missing values.

### Random sampling methodology and diversity estimation

Upon this screening, we observed extensive variation in sampling intensity among cities in the MetaSUB project, ranging from 1 to 548 samples. Consequently, cities with fewer than 14 samples from transportation systems were removed, resulting in a total of 32 cities included for subsequent analysis (Supplementary Table 5). Given the disparities in the number of samples collected per city, we standardized the sample size to 55 for each city to ensure a balanced comparative analysis. This was achieved using a drawing without replacement statistical technique. The number 55 was chosen because half of the cities had collected 55 or more samples from transportation systems. This selection process was iterated until the alpha diversity distribution of each city’s subsample was not significantly different from alpha diversity distribution of the total sample distribution, as determined by a Two Sample Kolmogorov–Smirnov test (Supplementary Table 5). This strategy allowed us to control for sampling bias and to ensure the robustness of our conclusions to differences in sample size across cities.

The alpha and beta diversities of the global urban microbiome data were calculated using the vegan package in R (version 2.6.2). Alpha diversity, the intra-sample diversity, was estimated based on three indices: the number of observed species, the Shannon entropy, and Fisher’s alpha diversity. The Shannon entropy reflects the diversity of the microbial community in the sample, taking into account both the richness and evenness of species distribution. The observed species index represents the richness of the sample community, regardless of the relative abundance of each species. Fisher’s alpha diversity, on the other hand, is a measure that combines both species richness and evenness.

Beta diversity, the inter-sample diversity, was measured using the Bray–Curtis distance metrix. A PERMANOVA analysis was conducted to examine the relationship between environmental/demographic characteristics and beta diversity using the Adonis function in the vegan package (version 2.6.2) in R (version 4.2.1). The analysis was performed with 999 permutations and was constrained by the “margin” option. A bivariate analysis was first conducted for each environmental/demographic characteristic, and characteristics with a p-value less than 0.05 were further included in the multivariate analysis. The multivariate analysis was conducted using a forward stepwise approach, adding the characteristic with the lowest *p*-value first and then adding the characteristic with the second lowest p-value. If the newly added characteristic did not reach the *p*-value cutoff of less than 0.05, it was removed from the model.

We performed a Detrended Correspondence Analysis (DCA) to evaluate the microbial distribution. The DCA output provides an “axis length,” which in our case was 5.8419 for DCA1. This value is a measure of species turnover across the environmental gradient represented in the dataset. Lower values (<3) indicate that species turnover is relatively low, suggesting linear relationships and rendering linear methods such as Redundancy Analysis (RDA) suitable. However, higher values (>4) suggest substantial species turnover and indicate that unimodal methods, such as Canonical Correlation Analysis (CCA), are more appropriate. Given the axis length of DCA1 (5.8419), indicating higher species turnover, we deemed a CCA more suitable than an RDA for assessing the correlation between microbial composition and environmental characteristics. Consequently, we conducted a CCA to visualize the impact of environmental characteristics on the urban microbiome composition.

Finally, regression analysis was conducted in Stata (version 15.1) to examine the relationship between global urban environmental and demographic characteristics and alpha diversity, pathogens, and AMR genes. Both bivariate and multivariate analyses were performed, with the multivariate analysis following the same forward stepwise approach as the PERMANOVA analysis.

### Reporting summary

Further information on research design is available in the Nature Research Reporting Summary linked to this article.

### Supplementary information

Supplementary Information file

Reporting Summary

## Data Availability

Authors can confirm that all relevant data are included in the paper and its supplementary information files.

## Code availability

The analysis and visualization codes utilized in this study have been included in the supplementary information of the manuscript.
