# Supplementary material for: Environmental determinants and demographic influences on global urban microbiomes, antimicrobial resistance and pathogenicity
Source: NPJ Biofilms Microbiomes. 2023 Dec 7;9:94. doi: 10.1038/s41522-023-00459-4 (PMC10703778; doi:10.1038/s41522-023-00459-4)
Supplement: Supplementary file 1 — Supplementary Information file [file 41522_2023_459_MOESM1_ESM.pdf]

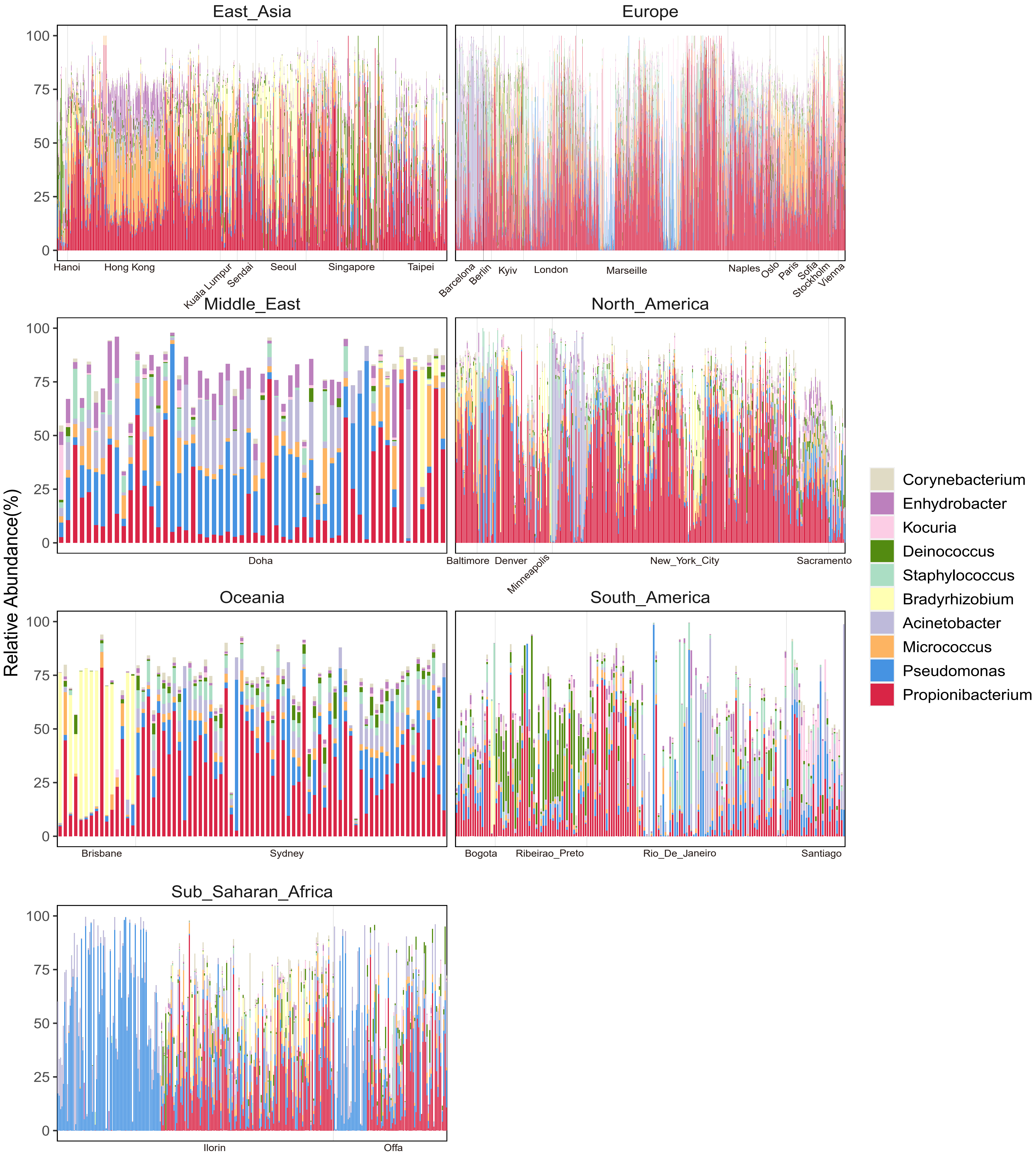

Supplementary Figure 1. Distribution of the top ten genus of all samples, grouped by continent.

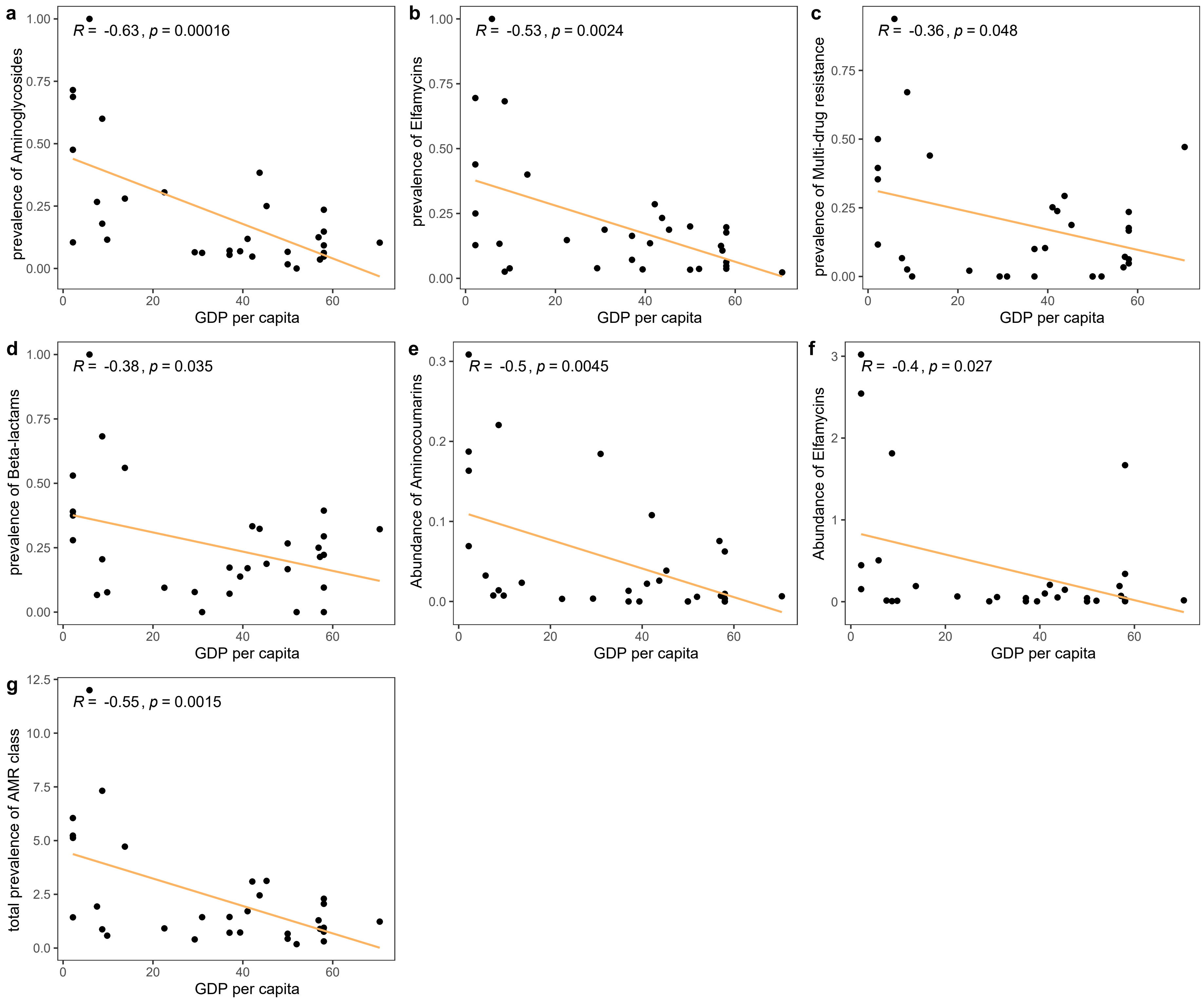

Supplementary Figure 2. The relationship between GDP per capita and the prevalence of Aminoglycosides (a), Elfamycins (b), Multi-drug resistance (c), Beta-lactams (d), total AMR class (g), and the abundance of Aminocoumarins (e), Elfamycins (f), as the main correlated environmental variable with AMR genes by drug class. Linear regression analysis and Pearson correlation coefficient was used.

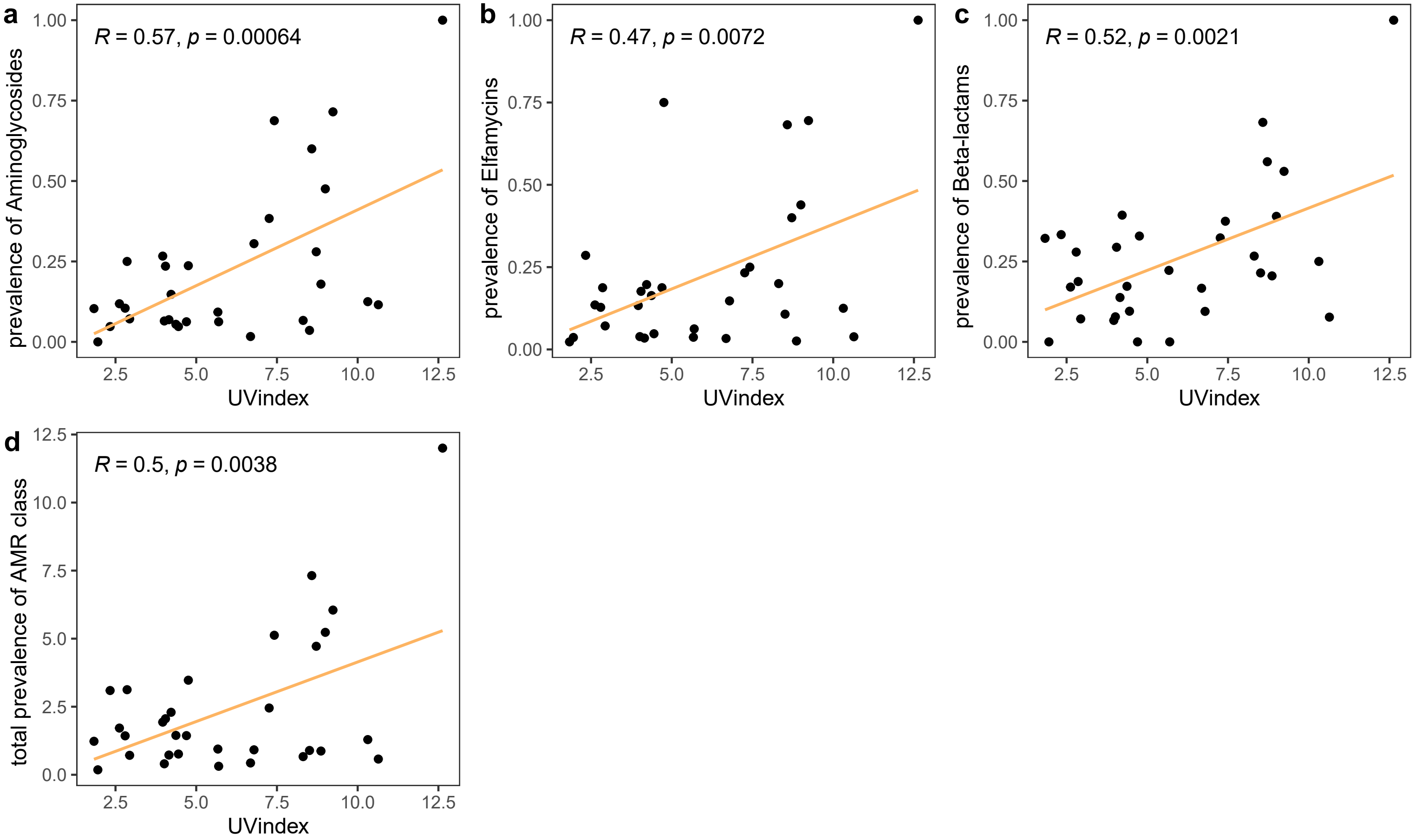

Supplementary Figure 3. The relationship between UVindex and the prevalence of Aminoglycosides (a), Elfamycins (b), Beta-lactams (c), total AMR classes (d). Linear regression analysis and Pearson correlation coefficient was used.

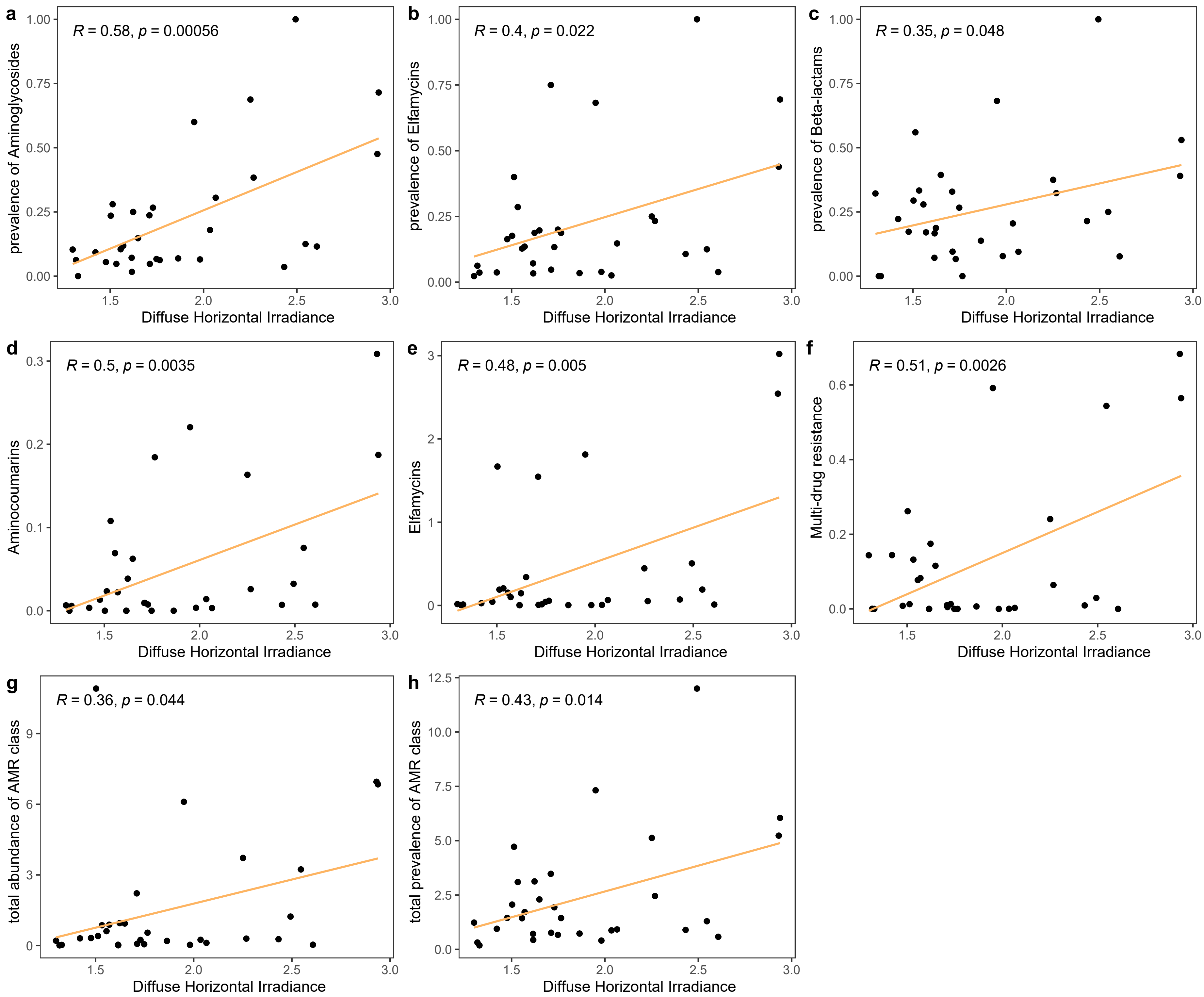

Supplementary Figure 4. The relationship between Diffuse Horizontal Irradiance and the prevalence of Aminoglycosides (a), Elfamycins (b), Beta-lactams (c), total AMR classes (h), and the abundance of Aminocoumarins (d), Elfamycins (e), Multi-drug resistance (f), total AMR classes (g). Linear regression analysis and Pearson correlation coefficient was used.

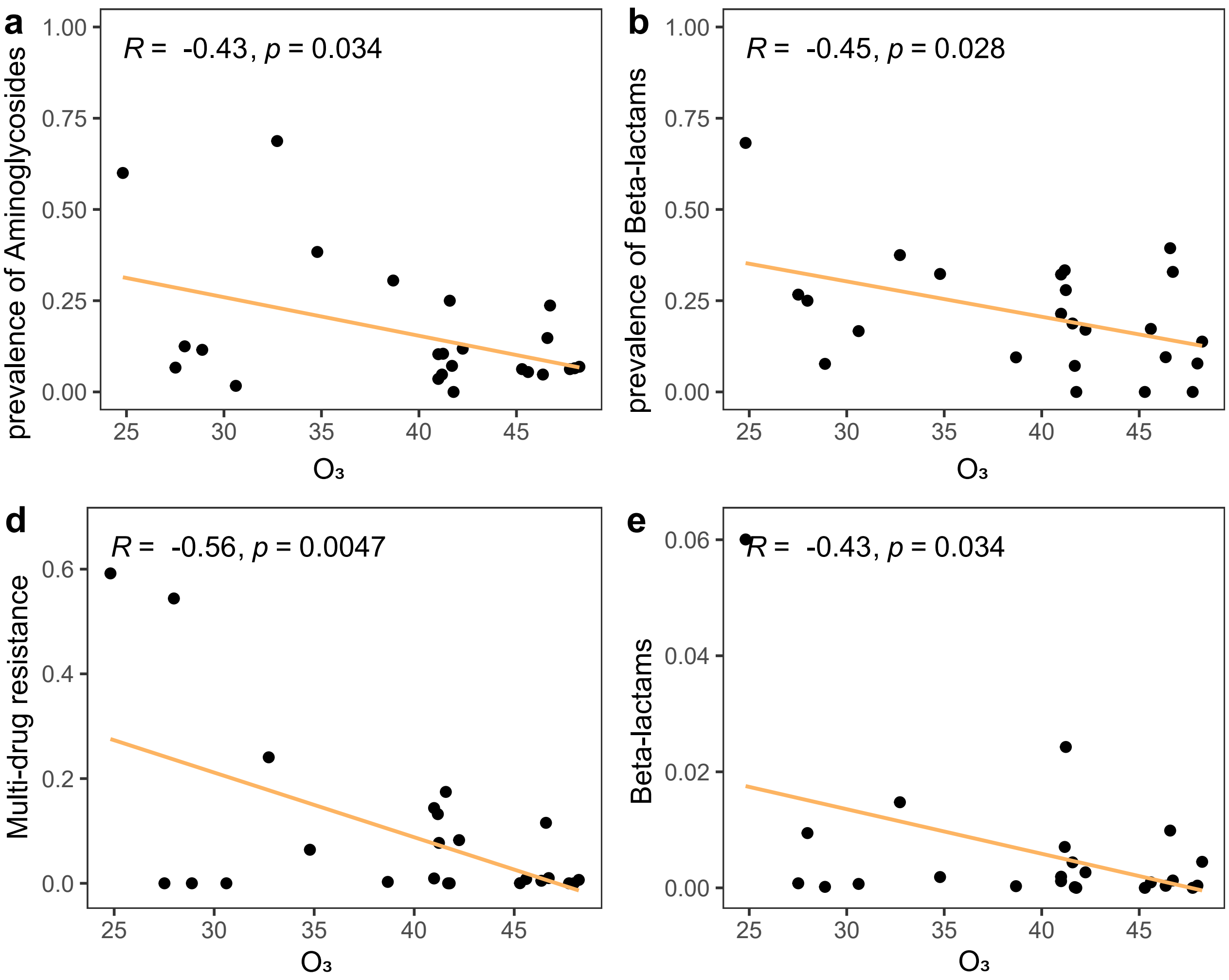

Supplementary Figure 5. The relationship between O3 concentration and the prevalence of Aminoglycosides (a), Beta-lactams (b), and the abundance of Aminoglycosides (c), Multi-drug resistance (d), Beta-lactams (e). Linear regression analysis and Pearson correlation coefficient was used.

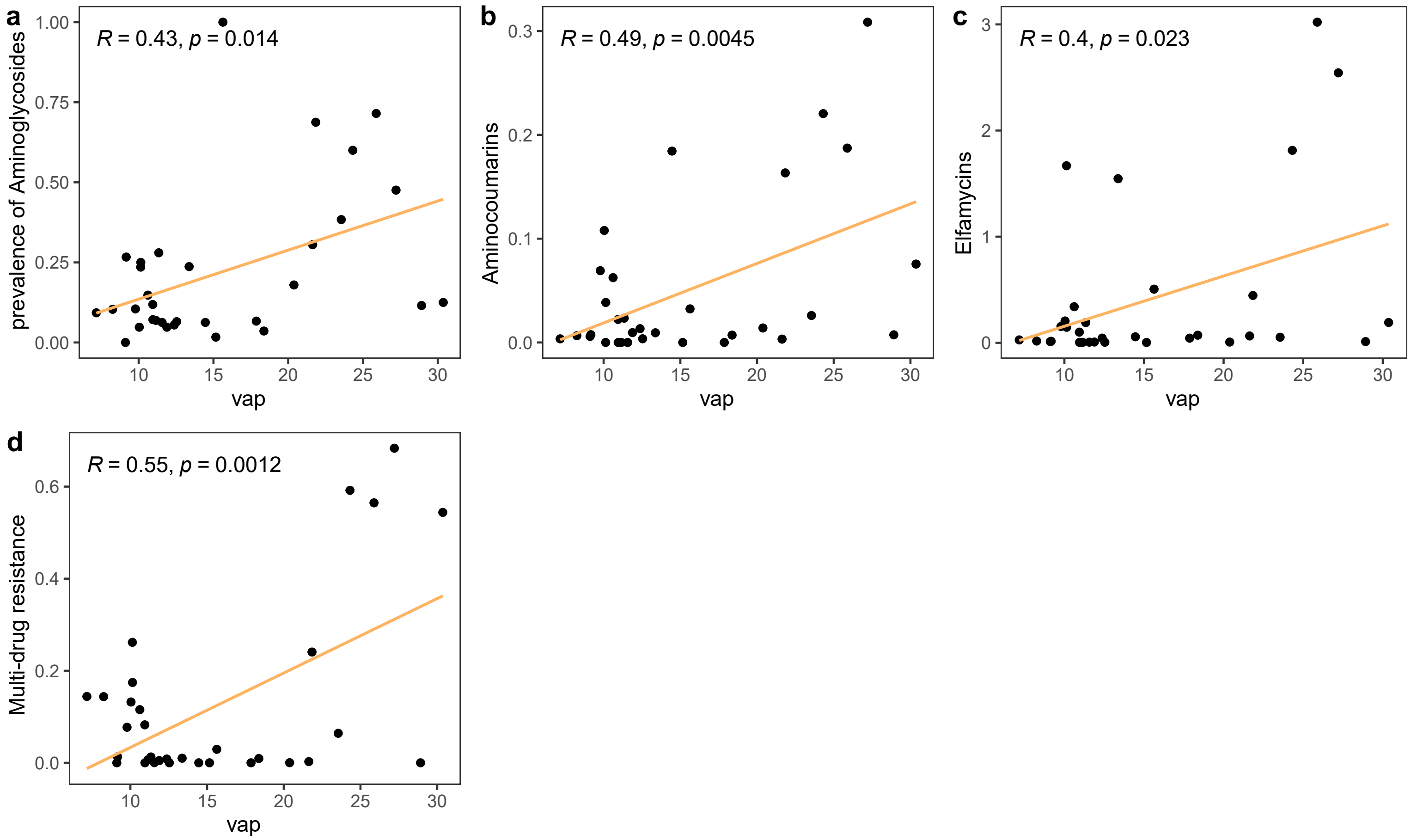

Supplementary Figure 6. The relationship between vapor pressure and the prevalence of Aminoglycosides (a), and the abundance of Aminocoumarins (b), Elfamycins (c), Multi-drug resistance (d). Linear regression analysis and Pearson correlation coefficient was used.

Supplementary Table 1. Details of environment and demographic data.

	environment factor	definition	unit	median(Q1,Q3)	NA% in 32 cities	date	time	database	link
air pollutant	PM2.5 concentration	annual average PM2.5 concentration	μg/m3	11.71(7.67,16.42)	0.00%	2016		GBD	Global Burden of Disea
	PM10 concentration	annual average PM10 concentration	μg/m3	19.14(16.15,28.11)	53.13%	2016		WHO	Air pollution (who.int)
	O3 concentration	annual average O3 concentration	ppbv	41.58(34.79,45.6)	25.00%	2016	Monthly average	EARTHDATA	https://giovanni.gsfc.na
	NO2 concentration	annual average NO2 concentration	μg/m3	29.24(19.2,39.62)	46.88%	2016	Monthly average	EARTHDATA	https://giovanni.gsfc.na
	CO concentration	annual average CO concentration	ppbv	136.91(132.56,138.74)	34.38%	2016	Monthly average	EARTHDATA	https://giovanni.gsfc.na
soil characteristics	soil ph	soil pH in H2O; 10 cm depth below surface,	*10-1	63(56,69.5)	0.00%	2016	yearly	Zenodo	https://zenodo.org/rec
	soil organic carbon density	Soil organic carbon density	t ha -1	12(7,16)	0.00%		yearly	Land-Atmosphere Interaction Research Group at Sun Yat-sen	http://globalchange.br
	soil organic carbon	soil organic carbon at 4.5cm;Organic Carbon is present in the soil organic matter fraction	% of weight, 0.01	185.64(131.32,346.47)	9.38%		yearly	Land-Atmosphere Interaction Research Group at Sun Yat-sen	http://globalchange.br
	total carbon	Total carbon in soils is the sum of both organic and inorganic C.	% of weight, 0.01	247.41(175.29,306.84)	0.00%		yearly	Land-Atmosphere Interaction Research Group at Sun Yat-sen	http://globalchange.br
	total phosphorus	total phosphorus in soil	% of weight, 0.0001	531.57(339.81,607.94)	0.00%		yearly	Land-Atmosphere Interaction Research Group at Sun Yat-sen	http://globalchange.br
	soil moisture	soil moisture(0-10cm)/Soil water Content: The water lost from soil upon drying to constant mass at 105 degrees Celcius; expressed either as the mass of water per unit mass of drysoil or as the volume of water per unit bulk volume of soil.	kg/m2	31.4(29.15,34.47)	6.25%	2016	Monthly average	EARTHDATA	https://giovanni.gsfc.na
	soil temperature	soil temperature(0-10cm): The temperature measured at a given soil depth, typically at 2, 4, 8, and sometimes 20 and 40 in. Many biological processes, including seed germination, plant emergence, microbial activity, and soil respiration are a function of soil temperature.	K	288.13(283.15,293.68)	6.25%	2016	Monthly average	EARTHDATA	https://giovanni.gsfc.na
precipitation	precipitation	annual average precipitation	mm	945.44(694.93,1279.49)	0.00%	2016	Monthly average	WorldClim	WorldClimWorldClim
	precipitation concentration index	Precipitation Concentration Index:The large the difference in monthly precipitation, the larger the concentration of precipitation during intra-annual, PCI values that less than 10 indicate a uniform monthly rainfall distribution in the year, whereas values from 11 to 20 denote seasonality of precipitation distribution. Values above 20 correspond to climates with substantial monthly variability in precipitation amounts, therefore, the greater the PCI value, the more variable the monthly precipitation.		10.96(9.81,12.65)	0.00%	2016	Monthly average	WorldClim	WorldClimWorldClim
	standard deviation of monthly precipitation	The standard deviation of monthly precipitation		39.0597(27.87901,75.45	0.00%	2016	Monthly average	WorldClim	WorldClimWorldClim
humidity	DHI(Diffuse Horizontal Irradiance)	Diffuse Horizontal Irradiance	W/m^2	3.87(2.97,4.7)	0.00%	2016	daily average	SOLARGIS	https://solargis.com/m
	UV	UV index	1(0~15)	4.73(2.62,7.26)	0.00%	2016		EARTHDATA	https://giovanni.gsfc.na
	wet(average rain day per month)	average rain day per month	Days	12.98(10.42,15.46)	0.00%	2016	Monthly average	CEDA Archive	https://catalogue.ceda
	vap	vapor pressure	Hecta-Pascals (x10)	12.38(10.19,17.59)	0.00%	2016	Monthly average	CEDA Archive	https://catalogue.ceda
	RH	Annual average relative humidity (RH)	%	70.96(65.95,74.97)	25.00%	2016	Monthly average	EARTHDATA	https://giovanni.gsfc.na
temperature	temperature	Annual average temperature	℃	14.52(10.22,18.75)	0.00%	2016	Monthly average	CEDA Archive	https://catalogue.ceda
	June temperature	average June temperature	℃	21.70(17.85,25.45)	9.38%			MetaSUB	Pangea (pangeabio.io)
	urban-rural temperature difference day	The difference (in degrees Celsius) in average summer daytime maximum land surface temperature between the urban area and buffer	℃	2.44(1.89,4.22)	21.88%	2013	summer daily average	Kaggle	https://www.kaggle.co
	urban-rural temperature difference night	The difference (in degrees Celsius) in average summer nighttime minimum land surface temperature between the urban area and buffer	℃	0.93(0.46,1.37)	21.88%	2013	summer daily average	Kaggle	https://www.kaggle.co
demographic data	city population	total population in the city	per ten thousand	16.248545(6.0535875,7	0.00%			MetaSUB	Pangea (pangeabio.io)
	population density	population density in the city	People per kilometer o	4310(2236,7282)	9.38%			MetaSUB	Pangea (pangeabio.io)
	Total antibiotic consumption	Global antibiotic consumption and usage in humans	daily doses per 1,000 p	18.49(12.075,25)	0.00%	2016		GRAM	Antibiotic usage and c
	GDP per Capita	GDP per Capita	per thousand US dollar	41.048(20.6185,55.627	3.13%	2016		DB.nomics	https://db.nomics.worl
others	adjacent to coast	Coastal cities are located on the interface or transition areas between land and sea, including large inland lakes.		1(0,1)	0.00%			MetaSUB	Pangea (pangeabio.io)
	elevation	elevation above sea level	meters	96(11.75,414.75)	0.00%			MetaSUB	Pangea (pangeabio.io)
	climate	1: equatorial; 2: arid; 3: warm temperate; 4: snow			0.00%	2016		Vetmeduni	World Maps of Köppe
	vegetation type	1=Tropical Evergreen Rain forest,Mangrove Forest;2=Tropical/Subtropical Evergreen Seasonal Broad leaved Forest;3=Temperate Evergreen Seasonal Broad leaved Forest,Summer Rain;4=Evergreen Broad leaved Sclerophyllous Forest, Winter Rain;5=Temperate/Subpolar Evergreen Needle leaved Forest;6=Tropical/Subtropical Drought-Deciduous Forest;7=Cold-Deciduous Forest,With Evergreens;8=Cold-Deciduous Forest,WithoutEvergreens;9=Xeromorphic Forest/Woodland;10=Evergreen Broad leaved Sclerophyllous Woodland;11=Tal/Medium/Short Grass land with 10-40% Woody Tree Cover;12=Tal/Medium/Short Grassland with Shrub Cover:13=Meadow .Short Grassland.No			0.00%	2016		EARTHDATA	https://webmap.ornl.g
	greenness fraction	Fraction of Vegetation Cover	%	0.92(0.89,0.94)	0.00%	2016		EARTHDATA	https://giovanni.gsfc.na
	soil type	1=Alfisols; 2=Andisols; 3=Entisols; 4=Gelisols; 5=Inceptisols; 6=Mollisols; 7=Oxisols; 8=Spodosols; 9=Ultisols; 10=Vertisols			0.00%	2015	yearly	USDA	https://www.nrcs.usda.
	fire carbon emissions	fire carbon emissions.Biomass burning C emissions.	g C m-2 month-1	0(0,2.77)	0.00%	2016		EARTHDATA	https://daac.ornl.gov/c

Supplementary Table 2. Results of Spearman's rank correlation coefficient between environment and demographic characteristics

env1	env2	spearman cor	spearman p	|cor|
city_ave_june_temp_c	pm2.5	0.622663184	5.07E-08	0.622663184
	SOCD	-0.690598992	3.78E-10	0.690598992
	TP	-0.597351407	2.36E-07	0.597351407
	sol_organi	-0.529434824	8.14E-06	0.529434824
	soil_tmp.0.10cm.	0.621314704	5.52E-08	0.621314704
	soil_tmp.10.40cm.	0.618749144	6.49E-08	0.618749144
	standard deviation of mon	0.574219788	8.63E-07	0.574219788
	PCI	0.502630572	2.69E-05	0.502630572
	Vegetation_type.0.12.	0.513882004	1.65E-05	0.513882004
	DIF	0.680189575	8.71E-10	0.680189575
	GHI	0.54254766	4.36E-06	0.54254766
	Uvindex	0.552492154	2.67E-06	0.552492154
	pet	0.64138155	1.48E-08	0.64138155
	vap	0.610071956	1.11E-07	0.610071956
	MAT	0.659451922	4.17E-09	0.659451922
	fire_carbon_emissions	0.58374964	5.12E-07	0.58374964
city_latitude	lat	0.980930397	0	0.980930397
	june_CO	0.842482621	0	0.842482621
	june_UV	-0.668499163	2.14E-09	0.668499163
	june_soil_tmp	-0.81022734	8.88E-16	0.81022734
	june_soil_mois	-0.703905749	1.23E-10	0.703905749
	june_vap	-0.719478683	3.07E-11	0.719478683
	june_tmp	-0.696520562	2.31E-10	0.696520562
	PERGDP	0.656202328	5.27E-09	0.656202328
	pm2.5	-0.549651958	3.08E-06	0.549651958
	o3	0.595939964	2.57E-07	0.595939964
	no2	0.519723267	1.27E-05	0.519723267
	co	0.688343038	4.54E-10	0.688343038
	SOCD	0.853554545	0	0.853554545
	OC	0.78236993	3.69E-14	0.78236993
	TC	0.788450407	1.71E-14	0.788450407
	TP	0.841221273	0	0.841221273
	sol_organi	0.817559806	4.44E-16	0.817559806
	soil_tmp.0.10cm.	-0.968692551	0	0.968692551
	soil_tmp.10.40cm.	-0.969545906	0	0.969545906
	pre_y	-0.765122356	2.86E-13	0.765122356
	standard deviation of mon	-0.914231645	0	0.914231645
	PCI	-0.647443234	9.78E-09	0.647443234
	Vegetation_type.0.12.	-0.871420266	0	0.871420266
	Vegetation_type.1.5.	-0.65423724	6.07E-09	0.65423724
	DIF	-0.904220199	0	0.904220199
	DNI	-0.62271658	5.05E-08	0.62271658
	GHI	-0.899459991	0	0.899459991
	Uvindex	-0.98334698	0	0.98334698
	pet	-0.88504727	0	0.88504727
	vap	-0.958234106	0	0.958234106
	MAT	-0.959464903	0	0.959464903
	fire_carbon_emissions	-0.865524197	0	0.865524197
city_longitude	june_pre	0.802477925	2.66E-15	0.802477925
	june_RH	0.556829782	2.14E-06	0.556829782
	june_wet	0.513580641	1.67E-05	0.513580641
	june_vap	0.51458419	1.60E-05	0.51458419
	PERGDP	-0.543858753	4.09E-06	0.543858753
	city_total_population	0.535939213	5.99E-06	0.535939213
	pm2.5	0.523551555	1.07E-05	0.523551555
	pre_y	0.735815596	6.43E-12	0.735815596
	standard deviation of mon	0.519481902	1.28E-05	0.519481902
	DIF	0.638322755	1.82E-08	0.638322755
	vap	0.535592872	6.09E-06	0.535592872
	fire_carbon_emissions	0.597466208	2.35E-07	0.597466208
city_population_density	pm2.5	0.593479907	2.96E-07	0.593479907
	pm10	0.646637286	1.03E-08	0.646637286
	N_T_DIFF	0.583155455	5.30E-07	0.583155455
	R_Humidity_night	-0.513551194	1.67E-05	0.513551194
city_total_population	pm2.5	0.69347514	2.98E-10	0.69347514
	pm10	0.604186955	1.58E-07	0.604186955
	soil_moisture.0.10cm.	0.68871897	4.40E-10	0.68871897
	soil_moisture.10.40cm.	0.727896029	1.39E-11	0.727896029
	pre_y	0.550070507	3.01E-06	0.550070507
	DIF	0.517508524	1.40E-05	0.517508524
co	SOCD	0.601948575	1.80E-07	0.601948575
	OC	0.597989199	2.28E-07	0.597989199
	TC	0.633399258	2.53E-08	0.633399258
	TP	0.576834544	7.49E-07	0.576834544
	sol_organi	0.676960188	1.12E-09	0.676960188
	soil_tmp.0.10cm.	-0.664588016	2.86E-09	0.664588016
	soil_tmp.10.40cm.	-0.668046881	2.21E-09	0.668046881
	PCI	-0.539643344	5.02E-06	0.539643344
	Vegetation_type.0.12.	-0.55147766	2.81E-06	0.55147766
	Vegetation_type.1.5.	-0.747759254	1.90E-12	0.747759254
	DNI	-0.758659091	5.90E-13	0.758659091

	GHI	-0.772836434	1.17E-13	0.772836434
	Uvindex	-0.707597918	8.94E-11	0.707597918
	pet	-0.741580464	3.60E-12	0.741580464
	vap	-0.59133727	3.34E-07	0.59133727
	MAT	-0.654882042	5.79E-09	0.654882042
coastal_city	PHH2O	-0.66640297	2.50E-09	0.66640297
	R_Humidity_day	0.505595003	2.37E-05	0.505595003
DIF	GHI	0.770879832	1.47E-13	0.770879832
	Uvindex	0.910531129	0	0.910531129
	pet	0.792808825	9.77E-15	0.792808825
	vap	0.958852672	0	0.958852672
	MAT	0.93924428	0	0.93924428
	fire_carbon_emissions	0.893754154	0	0.893754154
DNI	GHI	0.868749978	0	0.868749978
	Uvindex	0.670856545	1.79E-09	0.670856545
	wet	-0.753165504	1.07E-12	0.753165504
	pet	0.827523966	0	0.827523966
	R_Humidity_night	-0.635359548	2.22E-08	0.635359548
	vap	0.501895963	2.78E-05	0.501895963
	MAT	0.623800568	4.72E-08	0.623800568
GHI	Uvindex	0.942397858	0	0.942397858
	pet	0.983532375	0	0.983532375
	vap	0.847407187	0	0.847407187
	MAT	0.91996633	0	0.91996633
	fire_carbon_emissions	0.746871438	2.09E-12	0.746871438
june_CO	june_soil_tmp	-0.652544216	6.84E-09	0.652544216
	june_tmp	-0.519782396	1.27E-05	0.519782396
	o3	0.718273606	3.43E-11	0.718273606
	no2	0.579687321	6.41E-07	0.579687321
	co	0.876028341	0	0.876028341
	SOCD	0.771537854	1.36E-13	0.771537854
	OC	0.669649197	1.96E-09	0.669649197
	TC	0.795407346	6.88E-15	0.795407346
	TP	0.707655881	8.89E-11	0.707655881
	sol_organi	0.733688145	7.93E-12	0.733688145
	soil_tmp.0.10cm.	-0.828488144	0	0.828488144
	soil_tmp.10.40cm.	-0.830649545	0	0.830649545
	pre_y	-0.516677718	1.46E-05	0.516677718
	standard deviation of mon	-0.704071332	1.22E-10	0.704071332
	PCI	-0.560776823	1.75E-06	0.560776823
	Vegetation_type.0.12.	-0.746066508	2.27E-12	0.746066508
	Vegetation_type.1.5.	-0.67673219	1.14E-09	0.67673219
	DIF	-0.670890595	1.78E-09	0.670890595
	DNI	-0.62776225	3.66E-08	0.62776225
	GHI	-0.810680767	8.88E-16	0.810680767
	Uvindex	-0.851772562	0	0.851772562
	pet	-0.798019578	4.88E-15	0.798019578
	vap	-0.772434164	1.23E-13	0.772434164
	MAT	-0.804676612	1.78E-15	0.804676612
	fire_carbon_emissions	-0.666197754	2.54E-09	0.666197754
june_O3	june_CO	0.611525973	1.02E-07	0.611525973
	june_RH	-0.73950864	4.44E-12	0.73950864
	june_pet	0.655213174	5.66E-09	0.655213174
	o3	0.974682712	0	0.974682712
	PHH2O	0.612638033	9.48E-08	0.612638033
	pre_y	-0.590326986	3.54E-07	0.590326986
	N_T_DIFF	0.55350929	2.54E-06	0.55350929
	R_Humidity_night	-0.711433123	6.37E-11	0.711433123
	R_Humidity_day	-0.673469488	1.47E-09	0.673469488
	RH	-0.729252953	1.22E-11	0.729252953
june_pet	june_tmp	0.583426167	5.22E-07	0.583426167
	city_ave_june_temp_c	0.567373407	1.24E-06	0.567373407
	o3	0.600840374	1.93E-07	0.600840374
	PCI	0.592382825	3.15E-07	0.592382825
	N_T_DIFF	0.581759055	5.72E-07	0.581759055
	DNI	0.519930864	1.26E-05	0.519930864
	wet	-0.744159386	2.77E-12	0.744159386
	R_Humidity_night	-0.87222731	0	0.87222731
	R_Humidity_day	-0.571964618	9.74E-07	0.571964618
	RH	-0.75307936	1.08E-12	0.75307936
june_pre	june_O3	-0.560516721	1.78E-06	0.560516721
	june_RH	0.757099292	7.00E-13	0.757099292
	june_soil_mois	0.533469532	6.74E-06	0.533469532
	june_wet	0.718247455	3.44E-11	0.718247455
	june_pet	-0.610727682	1.07E-07	0.610727682
	PERGDP	-0.51209828	1.79E-05	0.51209828
	city_total_population	0.58015149	6.25E-07	0.58015149
	o3	-0.589800551	3.65E-07	0.589800551
	PHH2O	-0.542986744	4.27E-06	0.542986744
	pre_y	0.875593217	0	0.875593217
	standard deviation of mon	0.551977841	2.74E-06	0.551977841
	oriVegetation_type.0.31.	-0.548969316	3.18E-06	0.548969316
	DIF	0.595172486	2.68E-07	0.595172486
	wet	0.673049183	1.51E-09	0.673049183

	R_Humidity_night	0.565995261	1.34E-06	0.565995261
	RH	0.556623877	2.17E-06	0.556623877
	vap	0.534318901	6.47E-06	0.534318901
	fire_carbon_emissions	0.60323668	1.67E-07	0.60323668
june_RH	june_wet	0.555526551	2.29E-06	0.555526551
	june_pet	-0.70513568	1.11E-10	0.70513568
	o3	-0.66744588	2.31E-09	0.66744588
	PHH2O	-0.568377047	1.18E-06	0.568377047
	pre_y	0.757736013	6.53E-13	0.757736013
	N_T_DIFF	-0.598150679	2.26E-07	0.598150679
	wet	0.720236277	2.86E-11	0.720236277
	R_Humidity_night	0.775423791	8.57E-14	0.775423791
	R_Humidity_day	0.907618495	0	0.907618495
	RH	0.914590447	0	0.914590447
june_soil_mois	june_vap	0.512177285	1.78E-05	0.512177285
	PERGDP	-0.681364501	7.94E-10	0.681364501
	city_total_population	0.760617026	4.75E-13	0.760617026
	pm2.5	0.618628467	6.54E-08	0.618628467
	pm10	0.507344811	2.20E-05	0.507344811
	SOCD	-0.537949407	5.44E-06	0.537949407
	OC	-0.547955491	3.35E-06	0.547955491
	TP	-0.616504867	7.47E-08	0.616504867
	sol_organi	-0.521970463	1.15E-05	0.521970463
	soil_moisture.0.10cm.	0.874599568	0	0.874599568
	soil_moisture.10.40cm.	0.924475929	0	0.924475929
	soil_tmp.0.10cm.	0.634237724	2.39E-08	0.634237724
	soil_tmp.10.40cm.	0.634082224	2.42E-08	0.634082224
	pre_y	0.635918147	2.14E-08	0.635918147
	standard deviation of mon	0.686224786	5.39E-10	0.686224786
	Vegetation_type.0.12.	0.6746639	1.34E-09	0.6746639
	DIF	0.656818237	5.04E-09	0.656818237
	GHI	0.52649309	9.33E-06	0.52649309
	Uvindex	0.63811071	1.85E-08	0.63811071
	vap	0.643750534	1.26E-08	0.643750534
	MAT	0.615588345	7.91E-08	0.615588345
	fire_carbon_emissions	0.693105547	3.07E-10	0.693105547
june_soil_tmp	june_soil_mois	0.507730265	2.16E-05	0.507730265
	june_vap	0.947695172	0	0.947695172
	june_tmp	0.972315957	0	0.972315957
	PERGDP	-0.572655947	9.39E-07	0.572655947
	city_ave_june_temp_c	0.834137077	0	0.834137077
	pm2.5	0.671240714	1.74E-09	0.671240714
	co	-0.500852882	2.91E-05	0.500852882
	SOCD	-0.910528531	0	0.910528531
	OC	-0.784995427	2.66E-14	0.784995427
	TC	-0.709543224	7.53E-11	0.709543224
	TP	-0.840642717	0	0.840642717
	sol_organi	-0.839118605	0	0.839118605
	soil_tmp.0.10cm.	0.912325257	0	0.912325257
	soil_tmp.10.40cm.	0.910891269	0	0.910891269
	pre_y	0.582585363	5.47E-07	0.582585363
	standard deviation of mon	0.878211273	0	0.878211273
	PCI	0.766913875	2.33E-13	0.766913875
	D_T_DIFF	0.510779799	1.89E-05	0.510779799
	Vegetation_type.0.12.	0.780861325	4.44E-14	0.780861325
	DIF	0.86478676	0	0.86478676
	DNI	0.624770395	4.43E-08	0.624770395
	GHI	0.873550186	0	0.873550186
	Uvindex	0.88021584	0	0.88021584
	pet	0.928150168	0	0.928150168
	vap	0.865381614	0	0.865381614
	MAT	0.922426553	0	0.922426553
	fire_carbon_emissions	0.838768174	0	0.838768174
june_tmp	PERGDP	-0.530560432	7.72E-06	0.530560432
	city_ave_june_temp_c	0.899537797	0	0.899537797
	pm2.5	0.708464295	8.28E-11	0.708464295
	SOCD	-0.861472115	0	0.861472115
	OC	-0.715702913	4.33E-11	0.715702913
	TC	-0.617218676	7.14E-08	0.617218676
	TP	-0.788317847	1.73E-14	0.788317847
	sol_organi	-0.781406667	4.13E-14	0.781406667
	soil_tmp.0.10cm.	0.8167481	4.44E-16	0.8167481
	soil_tmp.10.40cm.	0.814601688	4.44E-16	0.814601688
	standard deviation of mon	0.802410495	2.66E-15	0.802410495
	PCI	0.760324289	4.91E-13	0.760324289
	Vegetation_type.0.12.	0.701292145	1.54E-10	0.701292145
	DIF	0.809277808	8.88E-16	0.809277808
	DNI	0.562987577	1.56E-06	0.562987577
	GHI	0.797417845	5.33E-15	0.797417845
	Uvindex	0.782055172	3.82E-14	0.782055172
	pet	0.862687017	0	0.862687017
	R_Humidity_night	-0.510180704	1.94E-05	0.510180704
	vap	0.770873447	1.47E-13	0.770873447
	MAT	0.841367669	0	0.841367669

	fire_carbon_emissions	0.777088341	7.02E-14	0.777088341
june_UV	june_soil_tmp	0.955509936	0	0.955509936
	june_pet	0.629350614	3.30E-08	0.629350614
	june_vap	0.921469663	0	0.921469663
	june_tmp	0.971166822	0	0.971166822
	city_ave_june_temp_c	0.849883515	0	0.849883515
	pm2.5	0.631644998	2.84E-08	0.631644998
	SOCD	-0.808957427	8.88E-16	0.808957427
	OC	-0.720160249	2.88E-11	0.720160249
	TC	-0.599952637	2.03E-07	0.599952637
	TP	-0.746436775	2.19E-12	0.746436775
	sol_organi	-0.803791711	2.22E-15	0.803791711
	soil_tmp.0.10cm.	0.795852401	6.44E-15	0.795852401
	soil_tmp.10.40cm.	0.793767922	8.44E-15	0.793767922
	standard deviation of mon	0.774930919	9.10E-14	0.774930919
	PCI	0.794330328	7.99E-15	0.794330328
	Vegetation_type.0.12.	0.672852858	1.54E-09	0.672852858
	DIF	0.747538313	1.95E-12	0.747538313
	DNI	0.631425049	2.88E-08	0.631425049
	GHI	0.815544891	4.44E-16	0.815544891
	Uvindex	0.770049178	1.62E-13	0.770049178
	pet	0.869366773	0	0.869366773
	R_Humidity_night	-0.568417204	1.18E-06	0.568417204
	vap	0.741945899	3.47E-12	0.741945899
	MAT	0.817433208	4.44E-16	0.817433208
	fire_carbon_emissions	0.723198866	2.17E-11	0.723198866
june_vap	june_tmp	0.960841009	0	0.960841009
	PERGDP	-0.670223498	1.88E-09	0.670223498
	city_ave_june_temp_c	0.889642075	0	0.889642075
	pm2.5	0.780871648	4.44E-14	0.780871648
	SOCD	-0.831213897	0	0.831213897
	OC	-0.633241082	2.56E-08	0.633241082
	TC	-0.556732775	2.16E-06	0.556732775
	TP	-0.74537292	2.44E-12	0.74537292
	sol_organi	-0.703470677	1.28E-10	0.703470677
	soil_tmp.0.10cm.	0.830333535	0	0.830333535
	soil_tmp.10.40cm.	0.827929549	0	0.827929549
	pre_y	0.645475004	1.12E-08	0.645475004
	standard deviation of mon	0.835431073	0	0.835431073
	PCI	0.623991596	4.66E-08	0.623991596
	Vegetation_type.0.12.	0.705646642	1.06E-10	0.705646642
	DIF	0.890014888	0	0.890014888
	GHI	0.721065033	2.65E-11	0.721065033
	Uvindex	0.783833004	3.06E-14	0.783833004
	pet	0.787227399	2.00E-14	0.787227399
	vap	0.829030474	0	0.829030474
	MAT	0.854805104	0	0.854805104
	fire_carbon_emissions	0.831505236	0	0.831505236
june_wet	june_pet	-0.697282064	2.17E-10	0.697282064
	co	0.588052809	4.03E-07	0.588052809
	OC	0.534952024	6.28E-06	0.534952024
	sol_organi	0.634388923	2.37E-08	0.634388923
	PCI	-0.656049503	5.33E-09	0.656049503
	DNI	-0.818665176	2.22E-16	0.818665176
	GHI	-0.582856817	5.38E-07	0.582856817
	wet	0.909805252	0	0.909805252
	pet	-0.555487816	2.30E-06	0.555487816
	R_Humidity_night	0.712824718	5.62E-11	0.712824718
	RH	0.576638233	7.57E-07	0.576638233
lat	june_CO	0.801236186	3.11E-15	0.801236186
	june_UV	-0.777053558	7.06E-14	0.777053558
	june_soil_tmp	-0.892391562	0	0.892391562
	june_soil_mois	-0.683939488	6.47E-10	0.683939488
	june_vap	-0.832063895	0	0.832063895
	june_tmp	-0.805631982	1.78E-15	0.805631982
	PERGDP	0.693352965	3.01E-10	0.693352965
	city_ave_june_temp_c	-0.58930301	3.75E-07	0.58930301
	pm2.5	-0.639077304	1.73E-08	0.639077304
	o3	0.522907654	1.10E-05	0.522907654
	co	0.619147928	6.33E-08	0.619147928
	SOCD	0.891291255	0	0.891291255
	OC	0.778125381	6.20E-14	0.778125381
	TC	0.782590731	3.57E-14	0.782590731
	TP	0.866968016	0	0.866968016
	sol_organi	0.82983166	0	0.82983166
	soil_tmp.0.10cm.	-0.988214879	0	0.988214879
	soil_tmp.10.40cm.	-0.988233805	0	0.988233805
	pre_y	-0.780818467	4.44E-14	0.780818467
	standard deviation of mon	-0.950423386	0	0.950423386
	PCI	-0.682067361	7.51E-10	0.682067361
	D_T_DIFF	-0.502432916	2.72E-05	0.502432916
	Vegetation_type.0.12.	-0.886432841	0	0.886432841
	Vegetation_type.1.5.	-0.593048941	3.03E-07	0.593048941
	DIF	-0.951731893	0	0.951731893

	DNI	-0.592390441	3.15E-07	0.592390441
	GHI	-0.905106531	0	0.905106531
	Uvindex	-0.990295144	0	0.990295144
	pet	-0.909524688	0	0.909524688
	vap	-0.978591624	0	0.978591624
	MAT	-0.986032604	0	0.986032604
	fire_carbon_emissions	-0.907289551	0	0.907289551
MAT	fire_carbon_emissions	0.87735727	0	0.87735727
N_T_DIFF	R_Humidity_night	-0.692201828	3.31E-10	0.692201828
	R_Humidity_day	-0.628265079	3.54E-08	0.628265079
	RH	-0.692648382	3.19E-10	0.692648382
no2	co	0.607193066	1.32E-07	0.607193066
	OC	0.50183951	2.79E-05	0.50183951
	TC	0.612111266	9.79E-08	0.612111266
	soil_tmp.0.10cm.	-0.506183116	2.31E-05	0.506183116
	soil_tmp.10.40cm.	-0.510424298	1.92E-05	0.510424298
	DNI	-0.515984594	1.50E-05	0.515984594
	GHI	-0.526610958	9.28E-06	0.526610958
	Uvindex	-0.524399638	1.03E-05	0.524399638
	pet	-0.54544833	3.79E-06	0.54544833
o3	co	0.552321056	2.69E-06	0.552321056
	PHH2O	0.593344965	2.98E-07	0.593344965
	TC	0.521664753	1.16E-05	0.521664753
	soil_tmp.0.10cm.	-0.51004694	1.95E-05	0.51004694
	soil_tmp.10.40cm.	-0.513816381	1.66E-05	0.513816381
	pre_y	-0.630095232	3.14E-08	0.630095232
	Vegetation_type.0.12.	-0.504862986	2.45E-05	0.504862986
	Vegetation_type.1.5.	-0.537758594	5.49E-06	0.537758594
	Uvindex	-0.53999187	4.94E-06	0.53999187
	R_Humidity_night	-0.616956265	7.26E-08	0.616956265
	R_Humidity_day	-0.556997322	2.13E-06	0.556997322
	RH	-0.610085862	1.11E-07	0.610085862
	vap	-0.565504778	1.37E-06	0.565504778
OC	TC	0.835393888	0	0.835393888
	TP	0.818755301	2.22E-16	0.818755301
	sol_organi	0.894862782	0	0.894862782
	soil_tmp.0.10cm.	-0.801026708	3.11E-15	0.801026708
	soil_tmp.10.40cm.	-0.800502765	3.55E-15	0.800502765
	standard deviation of mon	-0.756749117	7.27E-13	0.756749117
	PCI	-0.862053665	0	0.862053665
	Vegetation_type.0.12.	-0.766232843	2.52E-13	0.766232843
	Vegetation_type.1.5.	-0.555763419	2.26E-06	0.555763419
	DIF	-0.622199582	5.22E-08	0.622199582
	DNI	-0.782325604	3.69E-14	0.782325604
	GHI	-0.868905227	0	0.868905227
	Uvindex	-0.804317584	2.00E-15	0.804317584
	wet	0.547785097	3.37E-06	0.547785097
	pet	-0.887111223	0	0.887111223
	R_Humidity_night	0.518274978	1.36E-05	0.518274978
	vap	-0.717931312	3.54E-11	0.717931312
	MAT	-0.799544264	4.00E-15	0.799544264
	fire_carbon_emissions	-0.71712405	3.81E-11	0.71712405
oriVegetation_type	Vegetation_type.0.12.	-0.567175626	1.26E-06	0.567175626
	fire_carbon_emissions	-0.516624212	1.46E-05	0.516624212
PCI	Vegetation_type.0.12.	0.692149766	3.33E-10	0.692149766
	DIF	0.528379614	8.55E-06	0.528379614
	DNI	0.848329259	0	0.848329259
	GHI	0.860262716	0	0.860262716
	Uvindex	0.714027664	5.04E-11	0.714027664
	wet	-0.698195242	2.01E-10	0.698195242
	pet	0.856542035	0	0.856542035
	R_Humidity_night	-0.68619091	5.40E-10	0.68619091
	vap	0.587422633	4.17E-07	0.587422633
	MAT	0.703342363	1.29E-10	0.703342363
	fire_carbon_emissions	0.614155407	8.64E-08	0.614155407
PERGDP	city_total_population	-0.645743817	1.10E-08	0.645743817
	pm2.5	-0.8428986	0	0.8428986
	pm10	-0.668723357	2.10E-09	0.668723357
	SOCD	0.617037814	7.22E-08	0.617037814
	sol_organi	0.592234387	3.18E-07	0.592234387
	soil_tmp.0.10cm.	-0.655225463	5.65E-09	0.655225463
	soil_tmp.10.40cm.	-0.65286078	6.69E-09	0.65286078
	pre_y	-0.647271941	9.90E-09	0.647271941
	standard deviation of mon	-0.676332811	1.18E-09	0.676332811
	Vegetation_type.0.12.	-0.586848933	4.31E-07	0.586848933
	DIF	-0.766159369	2.54E-13	0.766159369
	GHI	-0.500943055	2.90E-05	0.500943055
	Uvindex	-0.640138507	1.61E-08	0.640138507
	vap	-0.690893424	3.69E-10	0.690893424
	MAT	-0.655852243	5.41E-09	0.655852243
	fire_carbon_emissions	-0.741626518	3.59E-12	0.741626518
pet	vap	0.86382055	0	0.86382055
	MAT	0.938481359	0	0.938481359
	fire_carbon_emissions	0.774655747	9.41E-14	0.774655747

PHH2O	pre_y	-0.639051028	1.74E-08	0.639051028
	oriVegetation_type.0.31.	0.551954593	2.74E-06	0.551954593
	RH	-0.501548791	2.82E-05	0.501548791
pm10	SOCD	-0.588619648	3.90E-07	0.588619648
	sol_organi	-0.605162314	1.49E-07	0.605162314
pm2.5	pm10	0.765208328	2.83E-13	0.765208328
	SOCD	-0.695113844	2.60E-10	0.695113844
	sol_organi	-0.607144704	1.32E-07	0.607144704
	soil_tmp.0.10cm.	0.593751612	2.91E-07	0.593751612
	soil_tmp.10.40cm.	0.590720841	3.46E-07	0.590720841
	standard deviation of mon	0.656380424	5.20E-09	0.656380424
	Vegetation_type.0.12.	0.553385814	2.55E-06	0.553385814
	DIF	0.72572934	1.71E-11	0.72572934
	Uvindex	0.568983196	1.14E-06	0.568983196
	pet	0.517576894	1.40E-05	0.517576894
	vap	0.591169304	3.37E-07	0.591169304
	MAT	0.613830368	8.81E-08	0.613830368
	fire_carbon_emissions	0.692746422	3.17E-10	0.692746422
pre_y	standard deviation of mon	0.799160473	4.00E-15	0.799160473
	D_T_DIFF	0.536303145	5.89E-06	0.536303145
	oriVegetation_type.0.31.	-0.58628383	4.45E-07	0.58628383
	Vegetation_type.0.12.	0.648843129	8.87E-09	0.648843129
	DIF	0.85126427	0	0.85126427
	Uvindex	0.71733189	3.74E-11	0.71733189
	R_Humidity_day	0.543328501	4.20E-06	0.543328501
	vap	0.824971844	0	0.824971844
	MAT	0.726424225	1.60E-11	0.726424225
	fire_carbon_emissions	0.782111914	3.80E-14	0.782111914
R_Humidity_day	RH	0.949892942	0	0.949892942
R_Humidity_night	R_Humidity_day	0.724646849	1.89E-11	0.724646849
	RH	0.896002076	0	0.896002076
dard deviation of monthly precipita	PCI	0.714882721	4.67E-11	0.714882721
	D_T_DIFF	0.543141482	4.24E-06	0.543141482
	oriVegetation_type.0.31.	-0.557324755	2.09E-06	0.557324755
	Vegetation_type.0.12.	0.885484155	0	0.885484155
	DIF	0.90358013	0	0.90358013
	DNI	0.531379525	7.43E-06	0.531379525
	GHI	0.834633703	0	0.834633703
	Uvindex	0.922249348	0	0.922249348
	pet	0.849758349	0	0.849758349
	vap	0.917886778	0	0.917886778
	MAT	0.922587175	0	0.922587175
	fire_carbon_emissions	0.925630979	0	0.925630979
SOCD	OC	0.820836975	2.22E-16	0.820836975
	TC	0.775123675	8.90E-14	0.775123675
	TP	0.817622384	4.44E-16	0.817622384
	sol_organi	0.865910056	0	0.865910056
	soil_tmp.0.10cm.	-0.913697849	0	0.913697849
	soil_tmp.10.40cm.	-0.911749543	0	0.911749543
	pre_y	-0.56031221	1.79E-06	0.56031221
	standard deviation of mon	-0.864390063	0	0.864390063
	PCI	-0.753610718	1.02E-12	0.753610718
	Vegetation_type.0.12.	-0.825385787	0	0.825385787
	Vegetation_type.1.5.	-0.522977457	1.10E-05	0.522977457
	DIF	-0.841000081	0	0.841000081
	DNI	-0.635257095	2.24E-08	0.635257095
	GHI	-0.875737571	0	0.875737571
	Uvindex	-0.890258389	0	0.890258389
	pet	-0.911103708	0	0.911103708
	vap	-0.85444187	0	0.85444187
	MAT	-0.913613043	0	0.913613043
	fire_carbon_emissions	-0.815159063	4.44E-16	0.815159063
soil_moisture.0.10cm.	soil_moisture.10.40cm.	0.988382524	0	0.988382524
soil_moisture.10.40cm.	oriVegetation_type.0.31.	-0.520372714	1.23E-05	0.520372714
soil_tmp.0.10cm.	soil_tmp.10.40cm.	0.999929409	0	0.999929409
	pre_y	0.737715438	5.32E-12	0.737715438
	standard deviation of mon	0.933758129	0	0.933758129
	PCI	0.698742599	1.92E-10	0.698742599
	D_T_DIFF	0.503404955	2.61E-05	0.503404955
	Vegetation_type.0.12.	0.892588663	0	0.892588663
	Vegetation_type.1.5.	0.582322687	5.55E-07	0.582322687
	DIF	0.928663559	0	0.928663559
	DNI	0.634014981	2.43E-08	0.634014981
	GHI	0.923747826	0	0.923747826
	Uvindex	0.990278779	0	0.990278779
	pet	0.936674383	0	0.936674383
	vap	0.979921075	0	0.979921075
	MAT	0.995709343	0	0.995709343
	fire_carbon_emissions	0.881015123	0	0.881015123
	pre_y	0.73786744	5.24E-12	0.73786744
	standard deviation of mon	0.932843722	0	0.932843722
	PCI	0.697460098	2.14E-10	0.697460098
	Vegetation_type.0.12.	0.892623736	0	0.892623736
	Vegetation_type.1.5.	0.586908172	4.29E-07	0.586908172

soil_tmp.10.40cm.	DIF	0.927805051	0	0.927805051
	DNI	0.634318433	2.38E-08	0.634318433
	GHI	0.923799674	0	0.923799674
	Uvindex	0.990701464	0	0.990701464
	pet	0.936541588	0	0.936541588
	vap	0.980236851	0	0.980236851
	MAT	0.995678898	0	0.995678898
	fire_carbon_emissions	0.880730164	0	0.880730164
soil_type	greenfraction	0.501431032	2.84E-05	0.501431032
sol_organi	soil_tmp.0.10cm.	-0.850609959	0	0.850609959
	soil_tmp.10.40cm.	-0.849677057	0	0.849677057
	standard deviation of mon	-0.767918802	2.07E-13	0.767918802
	PCI	-0.885403634	0	0.885403634
	Vegetation_type.0.12.	-0.754139074	9.66E-13	0.754139074
	Vegetation_type.1.5.	-0.609653896	1.14E-07	0.609653896
	DIF	-0.701430674	1.53E-10	0.701430674
	DNI	-0.8434829	0	0.8434829
	GHI	-0.945532698	0	0.945532698
	Uvindex	-0.865353664	0	0.865353664
	wet	0.58879509	3.86E-07	0.58879509
	pet	-0.93037887	0	0.93037887
	R_Humidity_night	0.520077275	1.25E-05	0.520077275
	vap	-0.760293037	4.92E-13	0.760293037
	MAT	-0.85110882	0	0.85110882
	fire_carbon_emissions	-0.71715274	3.80E-11	0.71715274
TC	TP	0.873045367	0	0.873045367
	sol_organi	0.734632926	7.23E-12	0.734632926
	soil_tmp.0.10cm.	-0.800015387	3.55E-15	0.800015387
	soil_tmp.10.40cm.	-0.799371806	4.00E-15	0.799371806
	pre_y	-0.501192576	2.87E-05	0.501192576
	standard deviation of mon	-0.771058187	1.44E-13	0.771058187
	PCI	-0.697278764	2.17E-10	0.697278764
	Vegetation_type.0.12.	-0.794172184	7.99E-15	0.794172184
	Vegetation_type.1.5.	-0.544650796	3.94E-06	0.544650796
	DIF	-0.635886525	2.15E-08	0.635886525
	DNI	-0.609810541	1.13E-07	0.609810541
	GHI	-0.787563458	1.91E-14	0.787563458
	Uvindex	-0.812888587	4.44E-16	0.812888587
	pet	-0.813929421	4.44E-16	0.813929421
	vap	-0.734137306	7.59E-12	0.734137306
	MAT	-0.781776109	3.95E-14	0.781776109
	fire_carbon_emissions	-0.74110909	3.78E-12	0.74110909
TP	sol_organi	0.766675244	2.39E-13	0.766675244
	soil_tmp.0.10cm.	-0.86673004	0	0.86673004
	soil_tmp.10.40cm.	-0.865284421	0	0.865284421
	pre_y	-0.570966755	1.03E-06	0.570966755
	standard deviation of mon	-0.827308318	0	0.827308318
	PCI	-0.710317199	7.03E-11	0.710317199
	Vegetation_type.0.12.	-0.850852882	0	0.850852882
	DIF	-0.778913205	5.64E-14	0.778913205
	DNI	-0.650349182	7.99E-09	0.650349182
	GHI	-0.85814033	0	0.85814033
	Uvindex	-0.8701323	0	0.8701323
	pet	-0.882085022	0	0.882085022
	vap	-0.813880254	4.44E-16	0.813880254
	MAT	-0.865478532	0	0.865478532
	fire_carbon_emissions	-0.780461839	4.66E-14	0.780461839
Uvindex	pet	0.938594222	0	0.938594222
	vap	0.965738239	0	0.965738239
	MAT	0.985102159	0	0.985102159
	fire_carbon_emissions	0.869386824	0	0.869386824
vap	MAT	0.982492382	0	0.982492382
	fire_carbon_emissions	0.88257808	0	0.88257808
Vegetation_type.0.12.	DIF	0.792114512	1.07E-14	0.792114512
	DNI	0.581241322	5.89E-07	0.581241322
	GHI	0.833406905	0	0.833406905
	Uvindex	0.886667318	0	0.886667318
	pet	0.832979328	0	0.832979328
	vap	0.869405173	0	0.869405173
	MAT	0.883904435	0	0.883904435
	fire_carbon_emissions	0.808674038	1.11E-15	0.808674038
Vegetation_type.1.5.	DIF	0.520849905	1.21E-05	0.520849905
	GHI	0.613130106	9.20E-08	0.613130106
	Uvindex	0.636748194	2.03E-08	0.636748194
	pet	0.596597807	2.47E-07	0.596597807
	vap	0.573635596	8.91E-07	0.573635596
	MAT	0.600669026	1.95E-07	0.600669026
wet	R_Humidity_night	0.795488691	6.66E-15	0.795488691
	R_Humidity_day	0.626146952	4.06E-08	0.626146952
	RH	0.770212488	1.59E-13	0.770212488

Supplementary Table 3. Specific data of environment and demographic characteristics in 59 cities

metasub_data											
**city**	**city_longi**	**city_latitu**	**coastal_ci**	**city_total_**	**city_popu**	**city_land_**	**city_ave_j**	**coastal**	**koppen_climate**	**elevation**	**continent**
**Auckland**	174.7667	-36.8667	1	16.29	1500	1102	10.6	('coastal', 'coastal')	marine_west_coast_climate	196	oceania
**Baltimore**	-76.6122	39.29038	1	6.21	2919	210	25.8	('coastal', 'coastal')	humid_subtropical_climate	150	north_america
**Barcelona**	2.15899	41.38879	1	16.21	16000	101	24.4	('coastal', 'coastal')	hot-summer_mediterranean_climate	12	europe
**Belfast**	-5.90167	54.61447	1	NA	NA	NA	NA	('low altitude', 'not_coastal')	warm summer continental climate	63	europe
**Berlin**	13.41053	52.52437	0	37.12	4200	891	19.4	('low altitude', 'not_coastal')	warm_humid_continental_climate	34	europe
**Birmingham**	-1.89589	52.47582	0	NA	NA	NA	NA	('low altitude', 'not_coastal')	marine_west_coast_climate	140	europe
**Bogota**	-74.0818	4.60971	0	80.81	4310	1587	16.1	('high altitude', 'not_coastal'	marine_west_coast_climate	2640	south_america
**Bradford**	-1.76045	53.79797	0	NA	NA	NA	NA	('low altitude', 'not_coastal')	marine_west_coast_climate	134	europe
**Brisbane**	153.0281	-27.4679	1	24.08	148	15842	15.3	('coastal', 'coastal')	humid_subtropical_climate	28	oceania
**Bury**	-2.27879	53.59794	0	NA	NA	NA	NA	('low altitude', 'not_coastal')	marine_west_coast_climate	304	europe
**Denver**	-104.985	39.73915	0	6.00	1745	401	20.3	('high altitude', 'not_coastal'	tropical_and_subtropical_steppe_climat	1600	north_america
**Doha**	51.52245	25.27932	1	13.51	10000	132	37.5	('coastal', 'coastal')	hot_desert_climate	10	middle_east
**Eastbourne**	0.267977	50.7727	1	NA	NA	NA	NA	('low altitude', 'not_coastal')	marine_west_coast_climate	34	europe
**Eden**	-2.7567	54.66394	0	NA	NA	NA	NA	('low altitude', 'not_coastal')	marine_west_coast_climate	79	europe
**Edinburgh**	-3.2023	55.95185	1	NA	NA	NA	NA	('low altitude', 'not_coastal')	marine_west_coast_climate	47	europe
**Fairbanks**	-147.716	64.83778	0	0.52	388	82	invalid mu	('low altitude', 'not_coastal')	continental_subarctic_climate	136	north_america
**Fukuoka**	130.4198	33.60994	1	NA	NA	NA	NA	('low altitude', 'not_coastal')	humid_subtropical_climate	8	east_asia
**Hamilton**	175.2833	-37.7833	0	2.03	230	877	17.8	('low altitude', 'not_coastal')	marine_west_coast_climate	95	oceania
**Hanoi**	105	21	0	75.88	2300	3329	29	('low altitude', 'not_coastal')	humid_subtropical_climate	16	east_asia
**Hong_Kong**	114.1577	22.28552	1	74.53	6777	1108	30.3	('coastal', 'coastal')	humid_subtropical_climate	957	east_asia
**Honolulu**	-157.858	21.30694	1	3.37	2236	177.2	27.5	('coastal', 'coastal')	tropical_rainforest_climate	6	oceania
**Ilorin**	4.54214	8.49664	0	7.78	1188	765	25.6	('low altitude', 'not_coastal')	tropical_savanna_climate	310	sub_saharan_africa
**Islington**	-0.11661	51.55719	0	NA	NA	NA	NA	('low altitude', 'not_coastal')	marine_west_coast_climate	34	europe
**Jaywick**	1.121304	51.77583	1	NA	NA	NA	NA	('low altitude', 'not_coastal')	marine_west_coast_climate	18	europe
**Kensington**	-0.19922	51.49879	0	NA	NA	NA	NA	('low altitude', 'not_coastal')	marine_west_coast_climate	14	europe
**Kuala_Lumpur**	101.6865	3.1412	0	17.90	6891	243	29.7	('low altitude', 'not_coastal')	tropical_rainforest_climate	66	east_asia
**Kyiv**	30.5238	50.45466	0	29.01	3299	839	23.9	('low altitude', 'not_coastal')	warm_humid_continental_climate	179	europe
**Lisbon**	-9.23083	38.75382	1	5.06	6458	100	23.9	('coastal', 'coastal')	hot-summer_mediterranean_climate	2	europe
**London**	-0.12574	51.50853	0	87.88	5590	1572	14.4	('low altitude', 'not_coastal')	marine_west_coast_climate	11	europe
**Marseille**	5.45	43.25	1	8.70	3600	NA	29	('coastal', 'coastal')	hot-summer_mediterranean_climate	0	europe
**Mexico_City**	99	19	0	89.19	6000	1485	18	('high altitude', 'not_coastal'	oceanic_subtropical_highland_climate	2250	north_america
**Minneapolis**	-93.2638	44.97997	0	3.83	2959	151	22.2	('low altitude', 'not_coastal')	cold_semi_arid_climates	253	north_america
**Montevideo**	-56.1882	-34.9033	1	17.19	6726	201	12.2	('coastal', 'coastal')	humid_subtropical_climate	43	south_america
**Naples**	14.24641	40.85631	1	9.67	8200	117	30.3	('coastal', 'coastal')	hot-summer_mediterranean_climate	0.9144	europe
**New_York_City**	-74.006	40.71427	1	86.23	11000	783.84	23.3	('coastal', 'coastal')	humid_subtropical_climate	10	north_america
**Newcastle**	-1.61239	54.97252	0	NA	NA	NA	NA	('low altitude', 'not_coastal')	marine_west_coast_climate	56	europe
**Offa**	4	8	0	0.90	NA	NA	NA	('low altitude', 'not_coastal')	tropical_savanna_climate	435	sub_saharan_africa
**Oslo**	10.74609	59.91273	1	6.73	1400	454	10	('coastal', 'coastal')	warm_humid_continental_climate	22.9	europe
**Paris**	2.3488	48.85341	0	22.06	21000	105	19.7	('low altitude', 'not_coastal')	marine_west_coast_climate	35	europe
**Porto**	-8.61099	41.14961	1	2.38	5700	41.42	20.6	('coastal', 'coastal')	warm-summer_mediterranean_climate	97	europe
**Ribeirao_Preto**	-47.8525	-21.1692	0	7.03293	NA	NA	NA	('low altitude', 'not_coastal')	tropical_savanna_climate	520	south_america
**Rio_De_Janeiro**	-43.2075	-22.9028	1	66.89	2705	1221	22.8	('coastal', 'coastal')	tropical_savanna_climate	5	south_america
**Sacramento**	-121.494	38.58157	0	4.66	1952	259	23.6	('low altitude', 'not_coastal')	hot-summer_mediterranean_climate	9.1	north_america
**San_Francisco**	-122.419	37.77493	1	8.84	7282	121	15.6	('coastal', 'coastal')	warm-summer_mediterranean_climate	16	north_america
**Santiago**	-70.6483	-33.4569	0	63.10	9821	641	8.3	('low altitude', 'not_coastal')	warm-summer_mediterranean_climate	570	south_america
**Sao_Paulo**	-46.6361	-23.5475	0	121.07	8959	1520	18.6	('low altitude', 'not_coastal')	humid_subtropical_climate	760	south_america
**Sendai**	140.8667	38.25759	1	10.86	1380	786	22.2	('coastal', 'coastal')	humid_subtropical_climate	1500	east_asia
**Seoul**	126.9784	37.566	0	98.39	16000	605	24.2	('low altitude', 'not_coastal')	hot_humid_continental_climate	38	east_asia
**Shanghai**	121	31	1	241.83	3800	6341	24	('coastal', 'coastal')	humid_subtropical_climate	4	east_asia
**Singapore**	103.8501	1.28967	1	57.92	7796	723.2	29.2	('coastal', 'coastal')	tropical_rainforest_climate	163.63	east_asia
**Sofia**	23.33	42.7	0	12.38	2517	492	19	('low altitude', 'not_coastal')	marine_west_coast_climate	500	europe
**Stockholm**	18.0649	59.33258	1	9.60	5100	188	13.1	('coastal', 'coastal')	warm_humid_continental_climate	0	europe
**Swansea**	-3.97798	51.60766	1	NA	NA	NA	NA	('low altitude', 'not_coastal')	marine_west_coast_climate	65	europe
**Sydney**	151.2069	-33.8839	1	48.23991	NA	NA	NA	('low altitude', 'not_coastal')	humid_subtropical_climate	19	oceania
**Taipei**	121.5319	25.04776	1	85.00	7500	1140	29.4	('coastal', 'coastal')	humid_subtropical_climate	1120	east_asia
**Tokyo**	139.6917	35.6895	1	138.40	2662	2188	21.7	('coastal', 'coastal')	humid_subtropical_climate	40	east_asia
**Vienna**	16.37208	48.20849	0	18.99	4326	395	25	('low altitude', 'not_coastal')	warm_humid_continental_climate	151	europe
**Yamaguchi**	131	34	1	1.99	194	1023	21.8	('coastal', 'coastal')	humid_subtropical_climate	157	east_asia
**Zurich**	8	47	0	4.09	4700	88	16	('low altitude', 'not_coastal')	marine_west_coast_climate	408	europe

air_pollution					soil										precipitation		
pm2.5	pm10	o3	no2	co	soil ph	soil orgar	soil_type	soil orgar	total carb	total pho	soil_mois	soil_mois	soil_tmp(	soil_tmp(	precipitat	standard	PCI
5.92	NA	29.75237	NA	77.4585	53	16	2	90.9101	158.108	288.782	33.16413	98.88027	289.9782	289.6883	1235.589	29.45424	8.958421
8.634367	NA	46.35755	24.1	135.4191	54	12	9	131.163	632.319	339.807	31.64492	95.37441	288.5561	288.4817	1052.284	33.37888	9.440136
12.53463	24.07	46.72474	39.19	133.546	52	4	5	144.465	267.461	558.631	30.56758	92.10003	292.6521	292.3382	610.6091	24.11573	10.04913
8.747455	18.81	42.34842	39.62	139.2156	56	52	2	NA	374.045	657.657	36.77107	111.0223	282.3456	282.1012	816.6778	20.44689	9.022851
14.28546	23.16	41.18097	30.37	136.5262	63	5	5	195.008	229	600	30.80621	90.60998	283.2175	283.0398	639.0946	37.76426	12.17416
11.41867	16.19	41.2914	21.31	137.058	48	15	3	125.429	159.797	358.231	27.35114	82.25762	283.1523	283.0288	789.1153	28.8987	9.808593
31.84177	NA	NA	NA	NA	53	37	5	578.438	536.262	593.076	32.21507	97.41745	288.4642	288.2607	1154.113	48.55358	10.28021
10.75545	NA	41.23973	46.09	137.5561	55	51	5	383.715	306.841	628.15	27.88105	83.31042	281.5027	281.3629	941.1478	29.19629	9.391934
4.317352	15.21	27.51378	7.22	77.44507	52	2	1	79.3409	16.1114	273.516	31.27084	94.41352	297.1607	296.8447	1007.684	61.30471	12.40462
9.514265	15.24	41.17816	29.64	137.962	71	33	5	1509.88	1469.94	699.227	29.96403	89.82325	281.6786	281.5963	1093.541	23.38572	8.836398
6.732575	28.1	NA	35	NA	60	11	6	117.712	161.8	543.237	32.59021	94.67437	285.5186	285.0861	569.3694	39.0597	13.51014
88.76576	NA	40.99329	NA	131.1673	67	NA	2	8.14391	179	20.8638	NA	NA	NA	NA	114.9563	23.51153	54.34725
NA	17.77	42.54452	12.25	137.8284	NA	14	9	281.828	299.443	553.929	26.05536	76.67504	284.3627	283.9353	890.9185	45.05923	11.14707
7.695349	NA	41.40933	NA	139.0737	56	16	5	724.14	337.032	669.511	28.42692	84.21126	279.9319	279.8132	865.2822	14.66384	8.64925
7.195759	NA	41.28257	NA	138.2903	59	27	1	366.662	127.104	359.321	29.19799	87.45593	281.226	281.0855	695.3277	24.76803	9.729047
2.814321	20.3	NA	NA	NA	61	30	4	2791.69	1203	600	31.37672	86.86286	274.1582	273.5423	427.8569	32.05384	14.50718
16.89717	NA	47.16202	NA	141.6794	64	10	9	216.197	422.517	602.627	36.34372	109.7546	291.4178	291.2286	2062.322	122.7488	12.23019
6.210583	NA	29.28088	20.33	76.84067	68	7	2	627	1639	1200	26.86048	79.3521	285.7114	285.8286	1148.033	25.32878	8.868775
34.32814	NA	32.72764	NA	135	69	9	9	160.502	203.813	502.403	28.01183	85.43351	295.2982	295.5908	1762.402	117.2809	13.20455
24.29226	NA	34.78623	NA	140.3887	71	2	5	NA	72.8189	126.275	36.7523	111.7162	297.1714	297.019	2734.027	153.2625	11.79001
4.327677	NA	33.26465	NA	124.4526	57	NA	2	238.082	187.061	397.724	13.85191	43.96642	299.6734	299.4821	1183.149	87.1204	14.29755
50.94361	NA	NA	NA	NA	64	8	1	143.88	161.539	500	22.82504	79.09687	300.9563	300.7728	1153.835	82.60119	13.97072
13.61919	NA	42.19747	NA	136.9144	73	15	5	180.051	284.378	684.141	34.47322	103.8248	284.5433	284.4404	580.2217	27.05352	10.72473
11.52115	NA	42.54354	NA	137.9726	74	12	6	286.881	241.312	571.493	32.20112	95.82454	283.9348	283.525	506.5103	23.08927	10.61913
14.34161	NA	42.24453	NA	136.8804	74	16	5	181.732	278.731	669.416	29.72807	89.32845	284.3283	284.2015	675.4219	27.87901	10.20745
16.34169	NA	28.88487	NA	122.4258	57	8	9	187.512	250.692	591.371	32.42783	97.94821	302.4538	302.2772	2214.66	90.92601	10.18753
20.41958	NA	41.2364	NA	136.9443	71	11	3	134.961	92.6054	176.402	34.78909	104.3882	282.4948	282.1706	694.925	27.04355	9.999216
9.868903	NA	46.3058	NA	132.556	60	5	5	124.003	237.179	696.902	23.30039	68.42542	290.8766	290.5852	727.3587	49.50768	13.42947
13.20107	NA	42.24426	9	136.906	76	15	5	180.051	284.378	684.141	34.47322	103.8248	284.5433	284.4404	765.2038	43.04587	11.81431
16.37748	26.35	45.59589	47.91	135.4467	63	15	5	NA	385.175	319.019	NA	NA	NA	NA	438.6781	29.78267	13.40357
22.4388	38.72	NA	27.86	NA	71	9	9	183.766	247.412	578.428	28.50083	85.98047	298.6625	298.8337	1529.924	98.84367	12.92479
6.899737	18.55	NA	11.6	NA	73	27	6	496.673	203.878	548.547	33.00008	95.65216	283.1455	283.2427	923.1306	47.96821	11.30345
8.968233	NA	29.67779	NA	76.52531	63	NA	10	NA	213.001	531.571	38.30033	115.3581	292.6345	292.3603	945.4397	36.12123	9.93898
14.47263	NA	47.74057	NA	133.6686	72	3	2	313.662	1308.59	999.21	29.33875	88.11168	292.9548	292.5391	749.6649	36.16844	10.89379
7.588338	NA	46.58686	25.77	136.7241	61	9	2	429.227	204.662	72.8318	29.71639	89.92212	287.7894	287.6951	1084.005	30.62376	9.211237
9.07397	11.36	41.55108	33.67	138.6507	67	16	5	238.253	253.978	684.904	29.12891	87.21948	282.5432	282.3805	604.8829	14.48579	8.964195
48.20771	NA	NA	NA	NA	66	7	1	129.88	114.51	233.501	25.04217	80.1934	300.341	300.2297	1333.472	72.64333	11.59783
9.140924	18.16	40.99335	40.37	NA	67	15	8	489.062	689.429	696.284	31.02617	93.13898	278.1315	277.4636	699.2312	24.86432	9.72426
14.60426	28.12	41.69506	50.87	135.5395	75	7	1	217.208	300.529	500	31.51976	95.13284	285.9434	285.8336	694.1502	30.61197	10.47262
7.270323	NA	NA	NA	135.1138	79	20	5	276.445	180.231	362.503	30.92475	93.26517	290.4659	290.2365	1227.174	74.13358	12.34764
12.73691	NA	NA	NA	NA	54	7	7	126	251	400	36.29412	112.8366	296.8531	296.8544	1476.225	91.86868	12.59346
11.09045	NA	24.81399	NA	86.24653	55	NA	9	132.271	313	153.989	34.96167	105.3403	299.1643	298.9607	1056.321	40.78112	9.972862
7.282394	14.78	45.28757	9.6	NA	60	15	3	160.363	230.976	407.68	33.67123	101.9616	291.9884	291.687	366.7219	35.89961	18.87474
7.570096	13.45	45.77112	15.8	137.2321	56	11	6	61.6371	100.371	322.1	29.6349	90.54553	289.0622	288.8569	688.8849	68.98143	19.36303
31.51672	66.83	NA	NA	NA	69	6	3	92.9552	117.713	500	37.83977	113.7202	292.3132	291.8369	739.1345	95.14825	26.56168
15.18672	28.25	25.34104	39.62	NA	56	12	7	228.506	251	400	39.0152	117.2837	295.7933	295.56	1362.399	85.36992	12.65244
9.781563	NA	48.2279	NA	140.9051	58	16	3	224.732	257.503	279.281	35.93136	107.6383	285.8088	285.6683	1213.225	70.52519	12.05039
29.74192	48	47.98453	58.28	143.8628	59	8	9	147	252	600	32.68848	98.41455	287.4654	287.2915	1279.491	101.5475	15.26211
39.73029	NA	44.29701	43	144.2187	52	7	5	183.124	294.592	375.439	36.17943	109.9181	289.5909	289.213	1124.496	64.85015	11.9918
15.6741	26	27.98634	26	117.8192	49	0	9	669.416	187	150.29	31.81055	96.5025	301.9597	301.8229	2335.503	75.45221	9.481423
27.21036	33.52	NA	22.98	NA	55	13	1	339.733	229.805	505.444	31.43277	91.76753	283.0773	282.6148	751.6374	30.63074	10.16013
5.671827	20.3	41.77447	28.84	138.1528	64	13	10	NA	175.288	613.504	25.7408	77.66463	280.6595	280.2724	524.5652	23.74119	10.58653
11.39012	19.14	42.17123	30.36	138.7446	55	24	9	517.595	397.822	607.941	34.984	105.0611	282.8994	282.8125	1064.757	26.5951	9.019603
7.591171	16.51	30.60954	14.69	77.48063	72	NA	8	70.7604	63.7583	244.45	28.63895	86.90441	293.9237	293.6478	1455.634	119.8641	15.7921
14.68329	NA	38.67941	NA	140.9604	80	5	5	132.674	146.977	430.389	35.35589	106.7048	295.9476	295.7677	3266.951	99.82137	9.360296
16.53523	NA	48.52045	NA	139.4556	63	18	5	403.725	1140.12	909.106	32.59847	98.51215	291.3283	291.1169	1613	68.31003	10.30618
12.85594	NA	41.58079	NA	NA	65	7	5	106.513	224.205	563.153	30.29791	89.07242	284.367	284.0782	751.8198	37.81973	11.1169
11.90298	NA	47.54892	NA	140.842	65	10	7	131.375	228	367.008	NA	NA	NA	NA	2091.289	104.4442	11.07701
11.36677	16.1	NA	33.89	NA	63	12	5	204.652	229.718	606.583	30.34869	90.72276	279.2103	278.7436	1363.273	66.68043	10.96496

heat_island		vegetation			solar[kWh/m2]				vapor						temperature	fire_carbon_emissions	koppen_climate2	GDP per Capita	main_climate
urban-rur	urban-rur	oriVegeta	Vegetatio	green frac	Diffuse H	Direct No	Global H	UVindex	wet	pet	umidity_n	umidity_	RH	vap					
4.184655	0.468551	28	2	0.519568	1.76	3.64	3.902	5.888793	14.4942	2.375	84.32032	68.09767	76.20899	14.875	15.90833	0.159355	32	40.08	warm
2.857058	0.568732	11	1	0.914581	1.712	4.227	4.15	4.448986	10.1775	3.083333	74.64064	52.37239	63.50652	11.88333	13.79167	0	31	58.021	warm
2.459533	1.139082	13	1	0.934522	1.71	4.731	4.417	4.755525	7.8783	3.25	76.52345	67.71614	72.1198	13.375	17.06667	0		26,549	warm
1.6008	-0.28597	11	1	0.828645	1.535	1.874	2.464	2.205815	16.1017	1.441667	84.82293	72.91667	78.8698	10.56667	9.725	0	32	41.048	warm
2.01991	1.347622	8	1	0.923127	1.533	2.628	2.901	2.332905	12.9800	2.125	76.20312	54.34245	65.27278	10.04167	10.51667	0	32	42.107	warm
2.15158	0.7674	11	1	0.930289	1.551	2.145	2.646	2.445448	12.7300	1.558333	78.80469	53.39845	66.10157	10.70833	10.16667	0	32	41.048	warm
3.034889	-1.94599	1	1	0.937447	2.493	3.349	4.742	12.63003	14.9142	2.475	NA	NA	NA	15.63333	16.21667	0.08254	32	5.87	warm
NA	NA	11	1	0.931004	1.527	2.034	2.546	2.312082	14.2042	1.45	78.78126	55.13412	66.95769	10.05833	9.183333	0	32	41.048	warm
2.307865	-0.78131	1	1	0.383865	1.748	5.179	4.898	8.310615	9.6142	4.083333	80.11458	64.77864	72.44661	17.86667	21.26667	0.565361	31	49.971	warm
NA	NA	11	1	0.93259	1.523	1.964	2.513	2.287064	15.5025	1.441667	80.56772	60.16276	70.36524	10.19167	9.366667	0.043612	32	NA	warm
4.955293	2.952912	28	2	0.897555	1.421	5.948	4.779	5.67031	7.7617	3.55	NA	NA	NA	7.166667	10.225	0	26	58.021	arid
-0.31417	1.751693	30	3	0.21137	2.432	5.005	5.837	8.508491	1.1517	7.066667	49.43229	42.43229	45.93229	18.375	29.125	0	22	57.162	arid
NA	NA	NA	NA	0.928909	1.573	2.895	3.139	3.016407	11.5950	1.775	81.90104	72.07291	76.98697	11.36667	11.39167	0	32	41.048	warm
NA	NA	11	1	0.68456	1.529	2.057	2.551	2.186398	16.0133	1.366667	82.39584	63.77343	73.08464	9.816667	8.641667	0	32	NA	warm
NA	NA	11	1	0.921496	1.476	1.857	2.417	1.979346	15.4608	1.358333	83.98178	62.38933	73.18555	9.558333	8.333333	0	32	41.048	warm
0.132727	1.334306	8	1	0.79016	NA	NA	NA	1.883531	8.5883	1.45	NA	NA	NA	5.866667	0.241667	0	43	58.021	snow
NA	NA	5	1	0.935866	1.965	2.975	3.862	4.924561	15.7992	2.775	76.18229	61.49219	68.83724	15.96667	17.16667	0	31	39.4	warm
1.180951	0.444378	28	2	0.974753	1.707	3.665	3.847	5.359426	13.7100	2.158333	84.72915	60.44662	72.58788	13.86667	14.51667	0	32	42.315	warm
0.782574	-0.03907	23	2	0.920142	2.251	1.886	3.677	7.417872	13.3817	3.141667	82.63542	59.95702	71.29622	21.84167	23.70833	16.97349	37	2.192	warm
NA	NA	2	1	0.547892	2.268	2.268	3.889	7.258766	16.4717	3.125	82.52865	70.91406	76.72135	23.55	23.6	5.676851	37	43.731	warm
7.061642	0.42833	13	1	NA	2.005	5.435	5.698	10.12546	13.9192	3.775	80.26042	72.79947	76.52995	22.59167	23.8	0	32	58.021	warm
2.562387	0.702586	NA	NA	0.889876	2.938	2.685	4.994	9.234736	8.9100	3.625	NA	NA	NA	25.88333	27.23333	9.89105	14	2.176	equatorial
NA	NA	11	1	0.927833	1.569	2.363	2.792	2.62154	11.5850	1.666667	78.36197	54.69402	66.52799	10.94167	10.7	0.011737	32	41.048	warm
NA	NA	11	1	0.900239	1.569	2.713	2.987	2.786788	14.2117	1.775	82.56772	71.44792	77.00782	11.19167	11.18333	0	32	NA	warm
NA	NA	11	1	0.927833	1.572	2.383	2.805	2.62154	11.5033	1.675	78.36197	54.69402	66.52799	10.875	10.51667	0	32	58.021	warm
2.050255	0.952482	1	1	0.94104	2.607	2.482	4.543	10.63971	19.1267	3.166667	84.20313	68.52605	76.36459	28.91667	26.43333	3.306612	11	9.817	equatorial
NA	NA	8	1	0.893424	1.556	2.999	3.205	2.798255	10.8567	2.116667	76.71614	50.08986	63.403	9.783333	9.8	8.498328	42	2.187	snow
NA	NA	13	1	0.740569	1.627	5.188	4.697	5.500316	10.5275	3.25	74.39584	67.78777	71.0918	14.275	17.775	5.120857	28	19.978	arid
2.640413	1.016044	11	1	0.927833	1.57	2.368	2.799	2.62154	11.5850	1.666667	78.36197	54.69402	66.52799	10.94167	10.7	0.011737	32	41.048	warm
NA	NA	13	1	0.947473	1.477	4.775	4.362	4.370143	10.5425	3.141667	75.4453	63.63803	69.54166	12.375	15.4	0	34	37.037	warm
7.395606	-0.19155	28	2	0.938623	2.402	3.7	4.984	9.367176	10.8575	3.475	NA	NA	NA	25.24167	26.33333	183.7457	14	NA	equatorial
2.032664	1.667722	11	1	0.796373	1.503	4.308	3.829	4.047702	10.8842	2.575	NA	NA	NA	10.13333	9.408333	9.167905	41	58.021	snow
0.196511	0.593673	25	2	0.94155	1.628	5.063	4.702	6.283023	9.6875	3.133333	76.50521	54.47656	65.49088	14.925	16.80833	0	31	NA	warm
NA	NA	10	1	0.929619	1.765	4.439	4.416	4.697157	10.4400	2.825	73.78906	68.07682	70.93294	14.45833	17.15833	0.049683	34	30.939	warm
4.343503	1.561933	11	1	0.88632	1.649	4.083	4.031	4.221608	10.4175	3.416667	74.80728	51.47266	63.13997	10.61667	13.29167	0	31	58.021	warm
NA	NA	11	1	0.929666	1.505	2.127	2.542	2.264145	14.4908	1.466667	82.375	63.72135	73.04818	9.883333	8.95	0	32	41.048	warm
1.970164	0.656775	23	2	0.91236	2.931	2.399	4.76	8.998062	9.5567	3.4	84.16928	NA	NA	27.20833	27.31667	49.12289	14	2.176	equatorial
1.538722	0.216821	10	1	0.957	1.299	2.806	2.606	1.833417	14.3658	1.583333	84.10676	69.30598	76.70637	8.258333	6.525	0	42	70.46	snow
2.640333	1.474924	11	1	0.912261	1.615	2.813	3.143	2.937564	13.7108	2.25	76.31252	50.30469	63.30861	10.95	12.49167	0	32	37.037	warm
1.839292	0.552321	10	1	0.946279	1.576	4.779	4.368	4.837737	12.7958	2.8	76.18489	67.07812	71.63151	13.84167	15.85833	0	35	19.978	warm
2.697417	1.423189	9	1	0.938802	2.035	4.928	5.31	8.862691	16.5267	3.225	NA	NA	NA	20.38333	23.39167	26.03781	14	8.71	equatorial
4.873962	1.911203	9	1	0.950449	1.95	4.195	4.831	8.578001	14.7692	3.066667	80.75522	66.89843	73.82683	24.31667	24.34167	0.059769	12	8.71	equatorial
NA	NA	8	1	0.933378	1.317	6.511	5.206	5.694605	5.3167	4.125	60.68622	38.48959	49.5879	11.56667	17.21667	8.046167	34	58.021	warm
NA	NA	6	1	0.951771	1.469	5.685	4.967	5.557291	5.6233	3.158333	70.86459	61.88152	66.37305	12.475	15.73333	0	35	58.021	warm
0.964597	1.102701	12	1	0.913727	1.513	6.449	5.553	8.716774	3.0450	3.091667	NA	NA	NA	11.34167	13.61667	0.394888	35	13.753	warm
4.440022	0.978752	9	1	0.892943	2.077	3.822	4.627	8.01193	18.3517	2.725	83.57813	76.63542	80.10678	19.28333	20.49167	0	31	8.71	warm
2.028352	1.140216	10	1	0.523805	1.864	2.967	3.593	4.154019	21.5175	2.141667	80.08072	59.54298	69.81185	11.15	11.04167	238.3903	31	39.4	warm
1.985432	1.345567	10	1	0.878822	1.981	3.35	3.938	4.01142	8.6992	2.525	73.34897	57.03386	65.19141	12.54167	12.29167	2.770404	49	29.288	snow
5.019442	0.898588	10	1	0.889943	2.134	2.299	3.673	5.259441	14.4833	2.558333	75.63282	61.79948	68.71615	17.59167	17.56667	628.9973	31	NA	warm
1.912663	1.194157	1	1	0.441317	2.546	2.549	4.509	10.31279	20.1842	3.466667	84.65104	68.29428	76.47266	30.36667	28.09167	0.151523	11	56.848	equatorial
NA	NA	10	1	0.892037	1.729	3.681	3.881	3.96226	7.9358	2.483333	NA	NA	NA	9.166667	10.69167	0	32	7.548	warm
2.482641	-0.39441	10	1	0.950509	1.328	2.96	2.713	1.953063	12.8367	1.525	82.78124	69.48698	76.13411	9.116667	7.366667	0	32	51.965	warm
NA	NA	11	1	0.93765	1.57	2.406	2.834	2.505962	15.2508	1.383333	82.89844	66.26563	74.58204	10.74167	9.558333	0	32	41.048	warm
5.084159	0.904471	6	1	0.970126	1.616	4.853	4.487	6.681353	11.4542	3.583333	79.95052	63.2578	71.60416	15.15833	18.75	0.230876	31	49.971	warm
3.809336	2.303532	2	1	0.924454	2.065	2.321	3.699	6.788897	18.7617	2.858333	79.88543	75.26302	77.57423	21.63333	21.14167	38.40085	31	22.54	warm
5.60867	1.433815	10	1	0.922019	1.849	3.374	3.86	4.726744	17.0875	2.65	76.97915	57.3646	67.17187	14.20833	15.99167	0	31	39.4	warm
NA	NA	10	1	0.887718	1.623	2.99	3.286	2.85837	13.8383	2.383333	78.37239	49.12565	63.74902	10.14167	11.34167	0	32	45.276	warm
NA	NA	5	1	0.933258	1.967	2.933	3.842	4.957915	16.8083	2.625	77.3776	64.59114	70.98437	15.96667	17.08333	0.509194	31	39.4	warm
2.427958	0.90023	11	1	0.91129	1.523	2.759	3.141	3.4392	18.2733	1.633333	NA	NA	NA	6.441667	4.141667	0	32	83.073	warm

Supplementary Table 4. Specific data of environment and demographic characteristics 32 cities

city	country	anti_consum	continent	city_longitud	city_latitude	city_total_population	city_population_densit	city_ave_june_tem	elevation_meters	pm25	pm10	o3	no2
**Denver**	USA	25	North_Am	-104.9847	39.73915	6.00158	1745	20.3	1600	6.732575	28.1	NA	35
**Stockholm**	Sweden	11.6	Europe	18.0649	59.33258	9.60031	5100	13.1	0	5.671827	20.3	41.77447	28.84
**New_York_City**	USA	25	North_Am	-74.00597	40.71427	86.22698	11000	23.3	10	7.588338	NA	46.58686	25.77
**Singapore**	Republic c	12.8	East_Asia	103.85007	1.28967	57.91901	7796	29.2	163.63	15.6741	26	27.98634	26
**Baltimore**	USA	25	North_Am	-76.61219	39.29038	6.20961	2919	25.8	150	8.634367	NA	46.35755	24.1
**Hanoi**	Vietnam	27.1	East_Asia	105	21	75.878	2300	29	16	34.32814	NA	32.72764	NA
**London**	England	20	Europe	-0.12574	51.50853	87.87892	5590	14.4	11	13.20107	NA	42.24426	9
**Hong_Kong**	China	8.8	East_Asia	114.15769	22.28552	74.5323	6777	30.3	957	24.29226	NA	34.78623	NA
**Ilorin**	Nigeria	7.5	Sub_Sahar	4.54214	8.49664	7.77667	1188	25.6	310	50.94361	NA	NA	NA
**Marseille**	France	29.2	Europe	5.45	43.25	8.69815	3600	29	0	16.37748	26.35	45.59589	47.91
**Taipei**	Taiwan	19.8	East_Asia	121.53185	25.04776	85	7500	29.4	1120	14.68329	NA	38.67941	NA
**Oslo**	Norway	14.3	Europe	10.74609	59.91273	6.73469	1400	10	22.9	9.140924	18.16	40.99335	40.37
**Kyiv**	Ukraine	12.2	Europe	30.5238	50.45466	29.0092	3299	23.9	179	20.41958	NA	41.2364	NA
**Rio_De_Janeiro**	Brazil	15.6	South_Am	-43.2075	-22.90278	66.8893	2705	22.8	5	11.09045	NA	24.81399	NA
**Offa**	Nigeria	7.5	Sub_Sahar	4	8	0.9	NA	NA	435	48.20771	NA	NA	NA
**Seoul**	Korea	10.5	East_Asia	126.9784	37.566	98.38892	16000	24.2	38	29.74192	48	47.98453	58.28
**Barcelona**	Spain	28.1	Europe	2.15899	41.38879	16.20809	16000	24.4	12	12.53463	24.07	46.72474	39.19
**Sydney**	Australia	25.7	Oceania	151.2068648	-33.88394	48.23991	NA	NA	19	7.591171	16.51	30.60954	14.69
**Doha**	Qatar	19.7	Middle_Ea	51.52245	25.27932	13.51	10000	37.5	10	88.76576	NA	40.99329	NA
**Ribeirao_Preto**	Brasil	15.6	South_Am	-47.8524681	-21.169223	7.03293	NA	NA	520	12.73691	NA	NA	NA
**Sendai**	Japan	14.3	East_Asia	140.8667	38.25759	10.86012	1380	22.2	1500	9.781563	NA	48.2279	NA
**Kuala_Lumpur**	Malaysia	9.9	East_Asia	101.68653	3.1412	17.9	6891	29.7	66	16.34169	NA	28.88487	NA
**Santiago**	Chile	11.7	South_Am	-70.64827	-33.45694	63.1	9821	8.3	570	31.51672	66.83	NA	NA
**Berlin**	Germany	17	Europe	13.41053	52.52437	37.1193	4200	19.4	34	14.28546	23.16	41.18097	30.37
**Bogota**	Colombia	8.8	South_Am	-74.08175	4.60971	80.80734	4310	16.1	2640	31.84177	NA	NA	NA
**Minneapolis**	USA	25	North_Am	-93.26384	44.97997	3.82578	2959	22.2	253	6.899737	18.55	NA	11.6
**Sacramento**	USA	25	North_Am	-121.4944	38.58157	4.66488	1952	23.6	9.1	7.282394	14.78	45.28757	9.6
**Naples**	Italy	24.2	Europe	14.24641	40.85631	9.67068	8200	30.3	0.9144	14.47263	NA	47.74057	NA
**Vienna**	Austria	17	Europe	16.37208	48.20849	18.99055	4326	25	151	12.85594	NA	41.58079	NA
**Brisbane**	Australia	25.7	Oceania	153.02809	-27.46794	24.08223	148	15.3	28	4.317352	15.21	27.51378	7.22
**Sofia**	Bulgaria	22.9	Europe	23.33	42.7	12.38438	2517	19	500	27.21036	33.52	NA	22.98
**Paris**	France	29.2	Europe	2.3488	48.85341	22.06488	21000	19.7	35	14.60426	28.12	41.69506	50.87
**NA**		0				0	3	3	0	0	17	8	15
**NA%**		0%				0.00%	9.38%	9.38%	0.00%	0.00%	53.13%	25.00%	46.88%

co	SPH	SOCD	SOC	TC	TP	soil_m	soil_mo2	soil_tmp	precipitatior	standard deviation of monthly precipitati	PCI	temp_diff_day	temp_diff_nigh
NA	60	11	117.712	161.8	543.237	32.59021	94.67437	285.5186	569.36944	39.05969588	13.51014	4.955292505	2.952911544
138.1528	64	13	NA	175.288	613.504	25.7408	77.66463	280.6595	524.5652	23.74118958	10.58653	2.482641071	-0.394412229
136.7241	61	9	429.227	204.662	72.8318	29.71639	89.92212	287.7894	1084.0051	30.62375502	9.211237	4.343503322	1.561932733
117.8192	49	8	669.416	187	150.29	31.81055	96.5025	301.9597	2335.5025	75.45220773	9.481423	1.91266336	1.194156609
135.4191	54	12	131.163	632.319	339.807	31.64492	95.37441	288.5561	1052.2839	33.3788775	9.440136	2.857058359	0.568732221
135	69	9	160.502	203.813	502.403	28.01183	85.43351	295.2982	1762.4017	117.280908	13.20455	0.78257406	-0.039067864
136.906	76	15	180.051	284.378	684.141	34.47322	103.8248	284.5433	765.2038	43.04587188	11.81431	2.640412913	1.016043538
140.3887	71	2	NA	72.8189	126.275	36.7523	111.7162	297.1714	2734.0268	153.2625004	11.79001	NA	NA
NA	64	8	143.88	161.539	500	22.82504	79.09687	300.9563	1153.83484	82.60118505	13.97072	2.562386936	0.702586265
135.4467	63	15	NA	385.175	319.019	NA	NA	NA	438.67805	29.78266877	13.40357	NA	NA
140.9604	80	5	132.674	146.977	430.389	35.35589	106.7048	295.9476	3266.951	99.8213707	9.360296	3.809335509	2.303532156
NA	67	15	489.062	689.429	696.284	31.02617	93.13898	278.1315	699.2312	24.86431654	9.72426	1.538721911	0.216821191
136.9443	71	11	134.961	92.6054	176.402	34.78909	104.3882	282.4948	694.92501	27.04355364	9.999216	NA	NA
86.24653	55	14	132.271	313	153.989	34.96167	105.3403	299.1643	1056.3211	40.78112336	9.972862	4.873962089	1.911202506
NA	66	7	129.88	114.51	233.501	25.04217	80.1934	300.341	1333.4723	72.6433277	11.59783	1.97016406	0.65677476
143.8628	59	8	147	252	600	32.68848	98.41455	287.4654	1279.491	101.5475016	15.26211	1.985432351	1.345566513
133.546	52	4	144.465	267.461	558.631	30.56758	92.10003	292.6521	610.6091	24.11572802	10.04913	2.459533251	1.139082472
77.48063	72	8	70.7604	63.7583	244.45	28.63895	86.90441	293.9237	1455.6335	119.8641362	15.7921	5.084158945	0.904470709
131.1673	67	9	8.14391	179	20.8638	NA	NA	NA	114.956257	23.51152794	54.34725	-0.314165148	1.751692585
NA	54	7	126	251	400	36.29412	112.8366	296.8531	1476.2248	91.86867806	12.59346	2.697417127	1.423189147
140.9051	58	16	224.732	257.503	279.281	35.93136	107.6383	285.8088	1213.2248	70.52518639	12.05039	2.028352154	1.140215975
122.4258	57	8	187.512	250.692	591.371	32.42783	97.94821	302.4538	2214.6604	90.9260148	10.18753	2.050254596	0.952482346
NA	69	6	92.9552	117.713	500	37.83977	113.7202	292.3132	739.13448	95.1482451	26.56168	0.964596908	1.102700775
136.5262	63	5	195.008	229	600	30.80621	90.60998	283.2175	639.0946	37.76426174	12.17416	2.019909992	1.347622409
NA	53	37	578.438	536.262	593.076	32.21507	97.41745	288.4642	1154.1129	48.55357762	10.28021	3.034888663	-1.945990721
NA	73	27	496.673	203.878	548.547	33.00008	95.65216	283.1455	923.13056	47.96821394	11.30345	2.032664007	1.667722417
NA	60	15	160.363	230.976	407.68	33.67123	101.9616	291.9884	366.721874	35.89960513	18.87474	NA	NA
133.6686	72	3	313.662	1308.59	999.21	29.33875	88.11168	292.9548	749.6649	36.16843913	10.89379	NA	NA
NA	65	7	106.513	224.205	563.153	30.29791	89.07242	284.367	751.81983	37.81972517	11.1169	NA	NA
77.44507	52	2	79.3409	16.1114	273.516	31.27084	94.41352	297.1607	1007.6837	61.30471499	12.40462	2.307865295	-0.781309271
NA	55	13	339.733	229.805	505.444	31.43277	91.76753	283.0773	751.63741	30.63073764	10.16013	NA	NA
135.5395	75	7	217.208	300.529	500	31.51976	95.13284	285.9434	694.1502	30.61196903	10.47262	2.640332594	1.474924365
11	0	0	3	0	0	2	2	2	0	0	0	7	7
34.38%	0.00%	0.00%	9.38%	0.00%	0.00%	6.25%	6.25%	6.25%	0.00%	0.00%	0.00%	21.88%	21.88%

greenfractior	DHI	DNI	GHI	UVindex	wet	pet	RH	vap	temperature	fire_carbon_emission	GDP	coastal_city	soil_type	oriVegetation
0.897555	1.421	5.948	4.779	5.67031	7.761667	3.55	NA	7.166667	10.2249999	0	58.021	0	6	28
0.950509	1.328	2.96	2.713	1.953063	12.83667	1.525	76.13411	9.116667	7.36666667	0	51.965	1	10	10
0.88632	1.649	4.083	4.031	4.221608	10.4175	3.416667	63.13997	10.61667	13.2916666	0	58.021	1	2	11
0.441317	2.546	2.549	4.509	10.31279	20.18417	3.466667	76.47266	30.36667	28.0916667	0.151523404	56.848	1	9	1
0.914581	1.712	4.227	4.15	4.448986	10.1775	3.083333	63.50652	11.88333	13.7916666	0	58.021	1	9	11
0.920142	2.251	1.886	3.677	7.417872	13.38167	3.141667	71.29622	21.84167	23.708333	16.97348911	2.192	0	9	23
0.927833	1.57	2.368	2.799	2.62154	11.585	1.666667	66.52799	10.94167	10.7000002	0.011736734	41.048	0	5	11
0.547892	2.268	2.268	3.889	7.258766	16.47167	3.125	76.72135	23.55	23.6000001	5.676851422	43.731	1	5	2
0.889876	2.938	2.685	4.994	9.234736	8.91	3.625	NA	25.88333	27.2333334	9.891050339	2.176	0	1	24
0.947473	1.477	4.775	4.362	4.370143	10.5425	3.141667	69.54166	12.375	15.4	0	37.037	1	5	13
0.924454	2.065	2.321	3.699	6.788897	18.76167	2.858333	77.57423	21.63333	21.1416667	38.40084754	22.54	1	5	2
0.957	1.299	2.806	2.606	1.833417	14.36583	1.583333	76.70637	8.258333	6.52500005	0	70.46	1	8	10
0.893424	1.556	2.999	3.205	2.798255	10.85667	2.116667	63.403	9.783333	9.8000001	8.498328209	2.187	0	3	8
0.950449	1.95	4.195	4.831	8.578001	14.76917	3.066667	73.82683	24.31667	24.3416665	0.059769455	8.71	1	9	9
0.91236	2.931	2.399	4.76	8.998062	9.556667	3.4	NA	27.20833	27.3166668	49.12289083	2.176	0	1	23
0.878822	1.981	3.35	3.938	4.01142	8.699167	2.525	65.19141	12.54167	12.2916666	2.77040432	29.288	0	9	10
0.934522	1.71	4.731	4.417	4.755525	7.878333	3.25	72.1198	13.375	17.0666668	0	26.549	1	5	13
0.970126	1.616	4.853	4.487	6.681353	11.45417	3.583333	71.60416	15.15833	18.7499999	0.230875835	49.971	1	8	6
0.21137	2.432	5.005	5.837	8.508491	1.151667	7.066667	45.93229	18.375	29.1250003	0	57.162	1	2	30
0.938802	2.035	4.928	5.31	8.862691	16.52667	3.225	NA	20.38333	23.3916669	26.03781331	8.71	0	7	9
0.523805	1.864	2.967	3.593	4.154019	21.5175	2.141667	69.81185	11.15	11.0416666	238.3902677	39.4	1	3	10
0.94104	2.607	2.482	4.543	10.63971	19.12667	3.166667	76.36459	28.91667	26.4333334	3.306612103	9.817	0	9	1
0.913727	1.513	6.449	5.553	8.716774	3.045	3.091667	NA	11.34167	13.6166667	0.39488842	13.753	0	3	12
0.923127	1.533	2.628	2.901	2.332905	12.98	2.125	65.27278	10.04167	10.5166667	0	42.107	0	5	8
0.937447	2.493	3.349	4.742	12.63003	14.91417	2.475	NA	15.63333	16.2166667	0.08253967	5.87	0	5	1
0.796373	1.503	4.308	3.829	4.047702	10.88417	2.575	NA	10.13333	9.40833314	9.167904556	58.021	0	6	11
0.933378	1.317	6.511	5.206	5.694605	5.316667	4.125	49.5879	11.56667	17.2166665	8.046166658	58.021	0	3	8
0.929619	1.765	4.439	4.416	4.697157	10.44	2.825	70.93294	14.45833	17.1583332	0.049683006	30.939	1	2	10
0.887718	1.623	2.99	3.286	2.85837	13.83833	2.383333	63.74902	10.14167	11.3416666	0	45.276	0	5	10
0.383865	1.748	5.179	4.898	8.310615	9.614167	4.083333	72.44661	17.86667	21.2666666	0.5653615	49.971	1	1	1
0.892037	1.729	3.681	3.881	3.96226	7.935833	2.483333	NA	9.166667	10.6916668	0	7.548	0	1	10
0.912261	1.615	2.813	3.143	2.937564	13.71083	2.25	63.30861	10.95	12.4916667	0	37.037	0	1	11
0	0	0	0	0	0	0	8	0	0	0	1	0	0	0
0.00%	0.00%	0.00%	0.00%	0.00%	0.00%	0.00%	25.00%	0.00%	0.00%	0.00%	3.13%	0.00%	0.00%	0.00%

Vegetation_type.1.5	koppen_climate	soil_type2	oriVegetation2	vegetation2	main_clim
2	tropical_and_subtropical_steppe_climate	Mollisols	Meadow ,Short Grassland,No WoodyCover	Grasslands	arid
1	warm_humid_continental_climate	Vertisols	Cold-Deciduous Forest,With Evergreens	Forests	warm
1	humid_subtropical_climate	Andisols	Cold-Deciduous Forest,WithoutEvergreens	Forests	warm
1	tropical_rainforest_climate	Ultisols	Tropical Evergreen Rain forest,Mangrove Forest	Forests	equatorial
1	humid_subtropical_climate	Ultisols	Cold-Deciduous Forest,WithoutEvergreens	Forests	warm
2	humid_subtropical_climate	Ultisols	Tal/Medium/Short Grass land with 10-40% Woody Tree Cover	Grasslands	warm
1	marine_west_coast_climate	Inceptisols	Cold-Deciduous Forest,WithoutEvergreens	Forests	warm
1	humid_subtropical_climate	Inceptisols	Tropical/Subtropical Evergreen Seasonal Broad leaved Forest	Forests	warm
2	tropical_savanna_climate	Alfisols	Tall/Medium/Short Grassland with gt 10% Woody TreeCover or Tuft-Plant Cov	Grasslands	equatorial
1	hot-summer_mediterranean_climate	Inceptisols	Evergreen Broad leaved Sclerophyllous Woodland	Forests	warm
1	humid_subtropical_climate	Inceptisols	Tropical/Subtropical Evergreen Seasonal Broad leaved Forest	Forests	warm
1	warm_humid_continental_climate	Spodosols	Cold-Deciduous Forest,With Evergreens	Forests	snow
1	warm_humid_continental_climate	Entisols	Temperate/Subpolar Evergreen Needle leaved Forest	Forests	snow
1	tropical_savanna_climate	Ultisols	Tropical/Subtropical Drought-Deciduous Forest	Forests	equatorial
2	tropical_savanna_climate	Alfisols	Tal/Medium/Short Grass land with 10-40% Woody Tree Cover	Grasslands	equatorial
1	hot_humid_continental_climate	Ultisols	Cold-Deciduous Forest,With Evergreens	Forests	snow
1	hot-summer_mediterranean_climate	Inceptisols	Evergreen Broad leaved Sclerophyllous Woodland	Forests	warm
1	humid_subtropical_climate	Spodosols	Evergreen Broad leaved Sclerophyllous Forest, Winter Rain	Forests	warm
3	hot_desert_climate	Andisols	Desert	Deserts	arid
1	tropical_savanna_climate	Oxisols	Tropical/Subtropical Drought-Deciduous Forest	Forests	equatorial
1	humid_subtropical_climate	Entisols	Cold-Deciduous Forest,With Evergreens	Forests	warm
1	tropical_rainforest_climate	Ultisols	Tropical Evergreen Rain forest,Mangrove Forest	Forests	equatorial
1	warm-summer_mediterranean_climate	Entisols	Xeromorphic Forest/Woodland	Forests	warm
1	warm_humid_continental_climate	Inceptisols	Temperate/Subpolar Evergreen Needle leaved Forest	Forests	warm
1	marine_west_coast_climate	Inceptisols	Tropical Evergreen Rain forest,Mangrove Forest	Forests	warm
1	cold_semi_arid_climates	Mollisols	Cold-Deciduous Forest,WithoutEvergreens	Forests	snow
1	hot-summer_mediterranean_climate	Entisols	Temperate/Subpolar Evergreen Needle leaved Forest	Forests	warm
1	hot-summer_mediterranean_climate	Andisols	Cold-Deciduous Forest,With Evergreens	Forests	warm
1	warm_humid_continental_climate	Inceptisols	Cold-Deciduous Forest,With Evergreens	Forests	warm
1	humid_subtropical_climate	Alfisols	Tropical Evergreen Rain forest,Mangrove Forest	Forests	warm
1	marine_west_coast_climate	Alfisols	Cold-Deciduous Forest,With Evergreens	Forests	warm
1	marine_west_coast_climate	Alfisols	Cold-Deciduous Forest,WithoutEvergreens	Forests	warm
0	0	0	0	0	0
0.00%	0.00%	0.00%	0.00%	0.00%	0.00%

Supplementary Table 5. Results of Two Sample Kolmogorov-Smirnov test between 55 subsamples and all samples

city	total size	sample size	p value			Kolmogorov-Smirnov Z		
			observed species	Shannon	Fisher alpha	observed species	Shannon	Fisher alpha
London	544	55	0.937	0.789	0.828	0.535	0.652	0.626
New_York_City	265	55	0.839	0.839	0.569	0.618	0.618	0.785
Hong_Kong	232	55	0.891	0.84	0.309	0.579	0.618	0.965
Ilorin	200	55	0.804	0.482	0.658	0.642	0.839	0.731
Singapore	118	55	0.998	0.998	0.784	0.389	0.389	0.655
Marseille	110	55	0.503	0.503	0.967	0.826	0.826	0.495
Taipei	96	55	0.687	0.481	0.917	0.715	0.84	0.555
Oslo	87	55	0.89	0.859	0.978	0.58	0.604	0.474
Kyiv	86	55	0.902	0.829	0.959	0.569	0.626	0.507
Rio_De_Janeiro	85	55	0.959	0.865	0.91	0.507	0.599	0.562
Offa	82	55	0.982	0.693	0.999	0.466	0.711	0.378
Seoul	77	55	0.999	0.964	0.602	0.383	0.5	0.765
Barcelona	75	55	0.998	0.933	0.905	0.389	0.539	0.567
Sydney	60	55	0.83	0.538	0.956	0.625	0.804	0.511
Doha	56	55	0.995	0.92	0.983	0.417	0.552	0.462
Denver	55	55	1	1	0.961	0	0	0.505
Stockholm	55	55	1	1	0.962	0	0	0.502
Ribeirao_Preto	39	55	1	0.982	0.999	0.305	0.465	0.374
Sendai	29	55	0.999	0.946	1	0.382	0.525	0.295
Kuala_Lumpur	26	55	1	0.756	1	0.35	0.673	0.347
Santiago	25	55	1	0.915	1	0.226	0.558	0.347
Berlin	21	55	0.997	0.875	1	0.4	0.592	0.122
Baltimore	21	55	1	1	1	0.324	0.324	0.144
Bogota	17	55	1	0.99	1	0.297	0.439	0.351
Minneapolis	17	55	0.922	0.523	1	0.551	0.813	0.22
Sacramento	16	55	0.971	0.257	1	0.488	1.012	0.06
Hanoi	16	55	0.998	0.998	1	0.395	0.395	0.146
Naples	16	55	0.997	0.982	0.999	0.405	0.466	0.362
Vienna	16	55	0.966	0.853	1	0.496	0.608	0.184
Brisbane	15	55	0.999	0.86	0.932	0.375	0.603	0.541
Sofia	15	55	1	0.95	0.964	0.166	0.52	0.499
Paris	14	55	1	0.903	0.987	0.33	0.568	0.451

Supplementary Table 6. Results of the Wilcoxon test on alpha diversity between different continents.(p-value < 0.05 as *< 0.01 as **< 0.005 as *** <0.0001 as ****)

.y.	group1	group2	p	p.adj	p.format	p.signif	method
observed_species	North_America	Europe	0.00000106	0.000018	0.0000011	****	Wilcoxon
	North_America	East_Asia	0.002415673	0.027	0.00242	**	Wilcoxon
	North_America	Sub_Saharan_Africa	0.462561905	0.93	0.46256	ns	Wilcoxon
	North_America	South_America	4.65E-11	9.8E-10	4.7E-11	****	Wilcoxon
	North_America	Oceania	0.198549335	0.6	0.19855	ns	Wilcoxon
	North_America	Middle_East	0.045413818	0.27	0.04541	*	Wilcoxon
	Europe	East_Asia	0.004814283	0.043	0.00481	**	Wilcoxon
	Europe	Sub_Saharan_Africa	0.007208353	0.058	0.00721	**	Wilcoxon
	Europe	South_America	0.0000104	0.00016	0.00001	****	Wilcoxon
	Europe	Oceania	0.00002	0.00028	0.00002	****	Wilcoxon
	Europe	Middle_East	0.0000212	0.00028	0.000021	****	Wilcoxon
	East_Asia	Sub_Saharan_Africa	0.148353943	0.59	0.14835	ns	Wilcoxon
	East_Asia	South_America	0.000000302	0.0000054	0.0000003	****	Wilcoxon
	East_Asia	Oceania	0.003068985	0.031	0.00307	**	Wilcoxon
	East_Asia	Middle_East	0.000185773	0.0022	0.00019	***	Wilcoxon
	Sub_Saharan_Africa	South_America	0.00000411	0.000066	0.0000041	****	Wilcoxon
	Sub_Saharan_Africa	Oceania	0.071355731	0.36	0.07136	ns	Wilcoxon
	Sub_Saharan_Africa	Middle_East	0.018793893	0.13	0.01879	*	Wilcoxon
	South_America	Oceania	7.13E-09	0.00000014	7.1E-09	****	Wilcoxon
	South_America	Middle_East	0.000000171	0.0000032	0.00000017	****	Wilcoxon
	Oceania	Middle_East	0.48588142	0.93	0.48588	ns	Wilcoxon
Shannon	North_America	Europe	1.06437E-06	0.000018	0.0000011	****	Wilcoxon
	North_America	East_Asia	0.002415673	0.027	0.00242	**	Wilcoxon
	North_America	Sub_Saharan_Africa	0.462561905	0.93	0.46256	ns	Wilcoxon
	North_America	South_America	4.65087E-11	9.8E-10	4.7E-11	****	Wilcoxon
	North_America	Oceania	0.198549335	0.6	0.19855	ns	Wilcoxon
	North_America	Middle_East	0.045413818	0.27	0.04541	*	Wilcoxon
	Europe	East_Asia	0.004814283	0.043	0.00481	**	Wilcoxon
	Europe	Sub_Saharan_Africa	0.007208353	0.058	0.00721	**	Wilcoxon
	Europe	South_America	1.03838E-05	0.00016	0.00001	****	Wilcoxon
	Europe	Oceania	2.00134E-05	0.00028	0.00002	****	Wilcoxon
	Europe	Middle_East	2.11515E-05	0.00028	0.000021	****	Wilcoxon
	East_Asia	Sub_Saharan_Africa	0.148353943	0.59	0.14835	ns	Wilcoxon
	East_Asia	South_America	3.01784E-07	0.0000054	0.0000003	****	Wilcoxon
	East_Asia	Oceania	0.003068985	0.031	0.00307	**	Wilcoxon
	East_Asia	Middle_East	0.000185773	0.0022	0.00019	***	Wilcoxon
	Sub_Saharan_Africa	South_America	4.10837E-06	0.000066	0.0000041	****	Wilcoxon
	Sub_Saharan_Africa	Oceania	0.071355731	0.36	0.07136	ns	Wilcoxon
	Sub_Saharan_Africa	Middle_East	0.018793893	0.13	0.01879	*	Wilcoxon
	South_America	Oceania	7.13193E-09	0.00000014	7.1E-09	****	Wilcoxon
	South_America	Middle_East	1.70542E-07	0.0000032	0.00000017	****	Wilcoxon
	Oceania	Middle_East	0.48588142	0.93	0.48588	ns	Wilcoxon
fisher_alpha	North_America	Europe	0.519340977	1	0.51934	ns	Wilcoxon
	North_America	South_America	2.19262E-12	4.2E-11	2.2E-12	****	Wilcoxon
	North_America	Oceania	0.013738424	0.14	0.01374	*	Wilcoxon
	North_America	Middle_East	0.07887207	0.47	0.07887	ns	Wilcoxon
	North_America	East_Asia	0.066101131	0.46	0.0661	ns	Wilcoxon
	North_America	Sub_Saharan_Africa	0.08326623	0.47	0.08327	ns	Wilcoxon
	Europe	South_America	2.13881E-17	4.5E-16	< 2e-16	****	Wilcoxon
	Europe	Oceania	0.00991718	0.11	0.00992	**	Wilcoxon
	Europe	Middle_East	0.021608194	0.19	0.02161	*	Wilcoxon
	Europe	East_Asia	0.006047397	0.073	0.00605	**	Wilcoxon
	Europe	Sub_Saharan_Africa	0.026392692	0.21	0.02639	*	Wilcoxon
	South_America	Oceania	7.18252E-13	1.4E-11	7.2E-13	****	Wilcoxon
	South_America	Middle_East	0.000834345	0.011	0.00083	***	Wilcoxon
	South_America	East_Asia	7.23502E-11	1.3E-09	7.2E-11	****	Wilcoxon
	South_America	Sub_Saharan_Africa	7.33465E-05	0.0012	0.000073	****	Wilcoxon
	Oceania	Middle_East	8.15075E-05	0.0012	0.000082	****	Wilcoxon
	Oceania	East_Asia	1.03754E-05	0.00018	0.00001	****	Wilcoxon
	Oceania	Sub_Saharan_Africa	0.000330675	0.0046	0.00033	***	Wilcoxon
	Middle_East	East_Asia	0.206645058	0.83	0.20665	ns	Wilcoxon
	Middle_East	Sub_Saharan_Africa	0.87501889	1	0.87502	ns	Wilcoxon
	East_Asia	Sub_Saharan_Africa	0.3949404	1	0.39494	ns	Wilcoxon

Supplementary Table 7. the relative abundance of pathogens defined based on the NIAID.

city	Brucella_ovis	Brucella_pinnipedialis	Clostridium_perfringens	Rickettsia_prowazekii	Salmonella_enterica	Salmonella_phage_HK620	Salmonella_phage_SSU5	Listeria_monocytogenes	Campylobacter_jejuni	Shigella_flexneri	Shigella_sonnei	Yersinia_enterocolitica
London	0.007131353	0.000490386	4.36436E-05	0	0.001883133	0	0	0.004811874	1.4063E-06	0	0	0
Rio_De_Janeiro	0	3.84193E-06	3.72287E-05	5.41956E-07	9.82237E-06	0	0.000109497	0	0	0	2.29136E-06	0
New_York_City	2.25077E-05	1.91019E-05	9.64394E-07	0	3.91061E-05	0	0	0	0	0	0	3.31325E-06
Auckland	0.014291739	0	0	0	0	0	0	0	0	0	0	0
Hong_Kong	0	1.68582E-06	0	0	0	0	0	0	0	0	0	0
Barcelona	6.03701E-06	0	0	0	0	0	0	1.31867E-05	0	0	1.46324E-05	0
Ilorin	2.81952E-06	7.04123E-05	2.59142E-06	0	3.60535E-07	0	0	0	0	0.000134597	1.52723E-05	0
Berlin	0	0	0	0	0	0	0	0	0	0	0	0
Bogota	7.92104E-06	5.39486E-06	0.000503076	0	0.000598135	0	0	2.22845E-05	0	0	0	0
Marseille	6.2641E-06	1.25845E-05	0	0	0	0	0	0	0	0	0	0
Denver	0	0	0	0	0.00161048	0	0	0	0	0	0	0
Taipei	0	0	0	0	0	0	0	0	0	0	0	0
Doha	0	1.37536E-05	0	0	0	0	0.001812023	0	0	0	0	0
Oslo	0	0	0	0	0	0	0	0	0	0	0	0
Kyiv	4.22439E-05	3.63921E-06	0	0	0	0	0	0	0	0	0	0
Offa	0	4.4089E-07	4.85518E-05	2.94916E-06	0	7.8974E-05	0	0	8.87083E-05	0	0.000105977	0
Seoul	0	3.68208E-05	0	0	0.000335196	0	0	0.0001258	0	0	0	0
Sacramento	0	0	0	0	0	0	0	0	0	0	0	0
Santiago	0	3.62238E-06	0.001742535	0	0.000583567	4.58927E-05	0	2.42295E-05	0	0	0	0
Sydney	0	0	6.55045E-06	0	0.001046839	0	0	0	0	0	0	0
Baltimore	0	0	0	0	0.00014934	0	0	0	0	0	0	0
Stockholm	0	0	0	0	0	0	0	0	0	0	0	0
Brisbane	0	0	0	0	2.01889E-05	0	0	0	0	0	0	0
Ribeirao_Preto	0	0	0	0	0	0	0	0	0	0	0	0
Sendai	1.83466E-05	1.9825E-05	0	0	0	0	0	0	0	0	0	0
Fukuoka	0	0	0	0	0	0	0	0	0	0	0	0
Hanoi	0	0	0	0	0	0	0	0	0	0	0	0
Kuala_Lumpur	0	0	0	0	0.001579352	0	0	0	0	0	0	0
Honolulu	0	0	0	0	0	0	0	0	0	0	0	0
Minneapolis	0	0	0	0	0.002514943	0	0	0.004246486	0	0	0	0
Naples	0	0	0	0	0	0	0	0	0	0	0	0
Paris	0	0	0	0	0	0	0	0	0	0	0	0
Vienna	0	0	0	0	0.002453419	0	0	1.31253E-05	0	0	0	0
Sofia	0	0	0	0	0	0	0	0	0	0	0	0
San_Francisco	0	0	0	0	0.02922483	0	0	0	0	0	0	0
Tokyo	0	0.000157892	0	0	0	0	0	0	0	0	0	0
Singapore	0	0	1.36533E-05	0	0	0	0	0	0	0	7.07439E-05	0
Yamaguchi	0	0	0	0	0	0	0	0	0	0	0	0

city	Hepatitis_C_virus	Influenza_A_virus	Bat_coronavirus_1A	Human_adenovirus_B	Human_adenovirus_D	Human_adenovirus_E	Human_adenovirus_F	Human_bocavirus	Human_cosavirus_E	Human_cyclovirus_VS5700009	Human_herpesvirus_1	Human_herpesvirus_4
London	0	0	0	0	1.02628E-05	1.93882E-05	0	0	0.001590057	0	0	5.29182E-05
Rio_De_Janeiro	6.42751E-07	5.98687E-06	5.93854E-06	1.32796E-05	6.85697E-05	0	0	0	2.4014E-05	4.84115E-06	0	1.34857E-05
New_York_City	0	0	0	1.29534E-05	7.81001E-06	4.73494E-06	0	1.03427E-05	1.59534E-05	0	0	0.000216369
Auckland	0	0	0	0	0	0	0	0	0	0	0	0
Hong_Kong	0	0	0	2.69368E-06	4.78473E-07	4.53063E-06	0	0	0	0	0.00016854	9.10808E-06
Barcelona	0	0	0	0	5.29378E-06	0	0	0	0	0	0	1.06695E-05
Ilorin	0	0	0	0	5.00406E-06	0	2.71648E-05	0	4.48871E-06	0	0	8.4968E-06
Berlin	0	0	0	0	0	0.000119282	0	0	0.00080651	0	0	0
Bogota	0	0	0	1.30915E-05	3.98392E-06	9.96419E-06	0	0	0.000278218	0	0	0.00028629
Marseille	0	0	0	2.10715E-05	0	0	0	0	0	0	0	8.69308E-05
Denver	0	0	0	0	0	0	0	0	0	0	0	0
Taipei	0	0	0	0	0	0	0	0	1.34965E-05	0	0	0.000843062
Doha	0	0	0	0	0	0	0	0	0	0	0	0
Oslo	0	0	0	0	0	0	0	0	0	0	0.000258752	0
Kyiv	0	0	0	0.000144541	0	0	0	0	0	0	0	0.000402127
Offa	0	0	0	0	9.93409E-05	0	0	0	6.51405E-07	0	0	2.81809E-06
Seoul	0	0	0	0	6.46616E-05	0	0	0	0	0	0	0
Sacramento	0	0	0	0	0	0	0	0.002001716	0	0	0	0
Santiago	7.5166E-06	0	0	2.19425E-06	0	2.46535E-05	0	0.001909752	0.000323425	0	0	0
Sydney	0	0	0	0	2.01013E-05	0	0	0.00031602	0	0	0	4.88137E-05
Baltimore	0	0	0	0	0	0	0	0	0	0	0	8.45484E-05
Stockholm	0	0	0	0	0	0	0	0	0	0	0	0
Brisbane	0	0	0	0	0	0	0	0	0	0	0	0
Ribeirao_Preto	0	0	0	3.46665E-05	0	0	0	0	0	0	0	6.19146E-05
Sendai	0	0	0	0	0	0	0	0	0	0	0.005444341	0
Fukuoka	0	0	0	0	0	0.000251087	0	0	0.000421029	0	0	0
Hanoi	0	0	0	0	0	0	0	0	0	0	0	0
Kuala_Lumpur	0	0	0	0	0	0	0	0	0	0	0	0.000503747
Honolulu	0	0	0	0	0	0	0	0	0	0	0	0
Minneapolis	0	0	0	0	0	0	0	0	0	0	0	0
Naples	0	0	0	0	0	0	0	0	0	0	0	0
Paris	0	0	0	0	0	0	0	0	0	0	0	0
Vienna	0	0	0	0	0	0	0	0	0	0	0	1.02045E-05
Sofia	0	0	0	0	0	0	0	0	0	0	0	0.000664602
San_Francisco	0	0	0	0	0	0	0	0	0	0	0	0
Tokyo	0	0	0	0	0	0	0	0	0	0	0	0
Singapore	0	0	0	0	1.06659E-05	2.45189E-05	0	0	0	0.000685368	0	0.001682708
Yamaguchi	0	0	0	0	0	0	0	0	0	0	0	0

city	Human_herpesvirus_5	Human_papillomavirus	Human_papillomavirus_type_134	Human_papillomavirus_type_135	Human_papillomavirus_type_137	Streptococcus_pyogenes	Streptococcus_agalactiae
London	0	0	0.000449196	0.000390424	0	3.88294E-06	1.08823E-05
Rio_De_Janeiro	0	0	0	0	0	0	0
New_York_City	4.40001E-05	6.40298E-05	2.13803E-05	0	0	1.02317E-05	2.22467E-07
Auckland	0	0	0	0	0	0	0
Hong_Kong	0	0	0.000737826	0	0	0	0
Barcelona	0	0.000124043	9.43663E-05	0	0.000125638	0	0
Ilorin	0	0	0	0	0	0	0
Berlin	0	0	0	0	0	0	0
Bogota	0	0	0.000270495	0	0	0	0
Marseille	0	0	0	0	0	0	0
Denver	0	0	0	0	0	0	0
Taipei	0	0	0	0	0	0	0
Doha	0	0	0	0	0	0	0
Oslo	0	0	0	0	0	0	0
Kyiv	0	0	0	0	0	0	0
Offa	0	0	0	0	0	0	0
Seoul	0	0	0	0	0	0	0
Sacramento	0	0	0	0	0	0	0
Santiago	0	0	0	0	0	0	0
Sydney	0	0	0	0	0	0	1.06369E-05
Baltimore	0	0	0	0	0	0	0
Stockholm	0	0	0	0	0	0	0
Brisbane	0	0	0	0	0	0	0
Ribeirao_Preto	0	0	0	0	0	0	0
Sendai	0	0	0	0	0	0	0
Fukuoka	0	0	0	0	0	0	0
Hanoi	0	0	0.001145975	0	0	0	0
Kuala_Lumpur	0	0	0	0	0	0	0
Honolulu	0	0	0	0	0	0	0
Minneapolis	0	0	0	0	0	0	0
Naples	0	0	0.000428762	0	0	0	0
Paris	0	0	0	0	0	0	0
Vienna	0	0.000260939	0	0	0	0	0
Sofia	0	0	0	0	0	0	0
San_Francisco	0	0	0	0	0	0	0
Tokyo	0	0	0	0	0	0	0
Singapore	0	0	0	0	0	0	0
Yamaguchi	0	0	0	0	0	0	0

Supplementary Table 8. the prevalence of pathogens defined based on the NIAID.

city	sample counts	Brucella_ovis	Brucella_pinnipedialis	Clostridium_perfringens	Rickettsia_prowazekii	Salmonella_enterica	Salmonella_phage_HK620	Salmonella_phage_SSU5	Listeria_monocytogenes	Campylobacter_jejuni	Shigella_flexneri	Shigella_sonnei
London	548	97	5	3	0	79	0	0	163	1	0	0
Rio_De_Janeiro	85	0	4	10	1	7	0	1	0	0	0	1
New_York_City	265	5	6	1	0	9	0	0	0	0	0	0
Auckland	8	1	0	0	0	0	0	0	0	0	0	0
Hong_Kong	232	0	1	0	0	0	0	0	0	0	0	0
Barcelona	76	1	0	0	0	0	0	0	2	0	0	1
Ilorin	200	1	6	2	0	5	0	0	0	0	1	1
Berlin	21	0	0	0	0	0	0	0	0	0	0	0
Bogota	17	1	1	12	0	15	0	0	1	0	0	0
Marseille	110	1	2	0	0	0	0	0	0	0	0	0
Denver	55	0	0	0	0	4	0	0	0	0	0	0
Taipei	96	0	0	0	0	0	0	0	0	0	0	0
Doha	56	0	1	0	0	0	0	1	0	0	0	0
Oslo	87	0	0	0	0	0	0	0	0	0	0	0
Kyiv	86	5	1	0	0	0	0	0	0	0	0	0
Offa	82	0	1	2	1	0	1	0	0	1	0	3
Seoul	77	0	2	0	0	2	0	0	2	0	0	0
Sacramento	16	0	0	0	0	0	0	0	0	0	0	0
Santiago	25	0	1	15	0	9	1	0	2	0	0	0
Sydney	60	0	0	1	0	2	0	0	0	0	0	0
Baltimore	21	0	0	0	0	2	0	0	0	0	0	0
Stockholm	55	0	0	0	0	0	0	0	0	0	0	0
Brisbane	15	0	0	0	0	1	0	0	0	0	0	0
Ribeirao_Preto	39	0	0	0	0	0	0	0	0	0	0	0
Sendai	29	1	2	0	0	0	0	0	0	0	0	0
Fukuoka	3	0	0	0	0	0	0	0	0	0	0	0
Hanoi	16	0	0	0	0	0	0	0	0	0	0	0
Kuala_Lumpur	26	0	0	0	0	5	0	0	0	0	0	0
Honolulu	7	0	0	0	0	0	0	0	0	0	0	0
Minneapolis	17	0	0	0	0	1	0	0	1	0	0	0
Naples	16	0	0	0	0	0	0	0	0	0	0	0
Paris	14	0	0	0	0	0	0	0	0	0	0	0
Vienna	16	0	0	0	0	2	0	0	1	0	0	0
Sofia	15	0	0	0	0	0	0	0	0	0	0	0
San_Francisco	8	0	0	0	0	5	0	0	0	0	0	0
Tokyo	8	0	1	0	0	0	0	0	0	0	0	0
Singapore	120	0	0	1	0	0	0	0	0	0	0	1
Yamaguchi	3	0	0	0	0	0	0	0	0	0	0	0

Yersinia_enterocolitica	Hepatitis_C_virus	Influenza_A_virus	Bat_coronavirus_1A	Human_adenovirus_B	Human_adenovirus_D	Human_adenovirus_E	Human_adenovirus_F	Human_bocavirus	Human_cosavirus_E	Human_cyclovirus_VS5700009	Human_herpesvirus_1	Human_herpesvirus_4
0	0	0	0	0	4	3	0	0	50	0	0	15
0	1	1	1	4	4	0	0	0	8	1	0	2
1	0	0	0	3	2	2	0	1	5	0	0	8
0	0	0	0	0	0	0	0	0	0	0	0	0
0	0	0	0	3	1	2	0	0	0	0	2	3
0	0	0	0	0	2	0	0	0	0	0	0	1
0	0	0	0	0	1	0	1	0	3	0	0	2
0	0	0	0	0	0	1	0	0	2	0	0	0
0	0	0	0	2	1	2	0	0	10	0	0	2
0	0	0	0	3	0	0	0	0	0	0	0	3
0	0	0	0	0	0	0	0	0	0	0	0	0
0	0	0	0	0	0	0	0	0	1	0	0	3
0	0	0	0	0	0	0	0	0	0	0	0	0
0	0	0	0	0	0	0	0	0	0	0	1	0
0	0	0	0	4	0	0	0	0	0	0	0	3
0	0	0	0	0	3	0	0	0	1	0	0	1
0	0	0	0	0	1	0	0	0	0	0	0	0
0	0	0	0	0	0	0	0	1	0	0	0	0
0	1	0	0	1	0	2	0	2	10	0	0	0
0	0	0	0	0	2	0	0	1	0	0	0	1
0	0	0	0	0	0	0	0	0	0	0	0	1
0	0	0	0	0	0	0	0	0	0	0	0	0
0	0	0	0	0	0	0	0	0	0	0	0	0
0	0	0	0	1	0	0	0	0	0	0	0	1
0	0	0	0	0	0	0	0	0	0	0	2	0
0	0	0	0	0	0	1	0	0	1	0	0	0
0	0	0	0	0	0	0	0	0	0	0	0	0
0	0	0	0	0	0	0	0	0	0	0	0	4
0	0	0	0	0	0	0	0	0	0	0	0	0
0	0	0	0	0	0	0	0	0	0	0	0	0
0	0	0	0	0	0	0	0	0	0	0	0	0
0	0	0	0	0	0	0	0	0	0	0	0	0
0	0	0	0	0	0	0	0	0	0	0	0	1
0	0	0	0	0	0	0	0	0	0	0	0	1
0	0	0	0	0	0	0	0	0	0	0	0	0
0	0	0	0	0	0	0	0	0	0	0	0	0
0	0	0	0	0	3	1	0	0	0	1	0	4
0	0	0	0	0	0	0	0	0	0	0	0	0

Human_herpesvirus_5	Human_papillomavirus	Human_papillomavirus_type_134	Human_papillomavirus_type_135	Human_papillomavirus_type_137	Streptococcus_pyogenes	Streptococcus_agalactiae	total_prevalence
0	0	2	1	0	1	3	0.77919708
0	0	0	0	0	0	0	0.541176471
1	1	1	0	0	1	1	0.181132075
0	0	0	0	0	0	0	0.125
0	0	2	0	0	0	0	0.060344828
0	1	1	0	1	0	0	0.131578947
0	0	0	0	0	0	0	0.115
0	0	0	0	0	0	0	0.142857143
0	0	1	0	0	0	0	2.823529412
0	0	0	0	0	0	0	0.081818182
0	0	0	0	0	0	0	0.072727273
0	0	0	0	0	0	0	0.041666667
0	0	0	0	0	0	0	0.035714286
0	0	0	0	0	0	0	0.011494253
0	0	0	0	0	0	0	0.151162791
0	0	0	0	0	0	0	0.170731707
0	0	0	0	0	0	0	0.090909091
0	0	0	0	0	0	0	0.0625
0	0	0	0	0	0	0	1.76
0	0	0	0	0	0	1	0.133333333
0	0	0	0	0	0	0	0.142857143
0	0	0	0	0	0	0	0
0	0	0	0	0	0	0	0.066666667
0	0	0	0	0	0	0	0.051282051
0	0	0	0	0	0	0	0.172413793
0	0	0	0	0	0	0	0.666666667
0	0	1	0	0	0	0	0.0625
0	0	0	0	0	0	0	0.346153846
0	0	0	0	0	0	0	0
0	0	0	0	0	0	0	0.117647059
0	0	1	0	0	0	0	0.0625
0	0	0	0	0	0	0	0
0	1	0	0	0	0	0	0.3125
0	0	0	0	0	0	0	0.066666667
0	0	0	0	0	0	0	0.625
0	0	0	0	0	0	0	0.125
0	0	0	0	0	0	0	0.091666667
0	0	0	0	0	0	0	0

Supplementary Table 9. Results of regression analysis, observed species VS environment and demographic characteristics

Green = p<=0.05, after regression diagnosis

White = p>0.05

observed species	Coef.	Std. Err.	t	P>t	[95% Conf.	Interval]	AdjR-squared	F
pm10	1.664698	0.4752099	3.5	0.0044	0.629305	2.700092	0.4644	F(1 12) = 12.27
soil organic carbon density	2.654975	0.9829021	2.7	0.0118	0.6382262	4.671723	0.1836	F(1 27) = 7.30
urban-rural temperature difference nigh	-21.3304	8.587104	-2.48	0.0207	-39.09418	-3.566619	0.1772	F(1 23) = 6.17
UVindex	5.944834	2.434957	2.44	0.0207	0.9719886	10.91768	0.1379	F(1 30) = 5.96
GDP	-0.7020717	0.3364952	-2.09	0.0458	-1.390282	-0.0138618	0.1005	F(1 29) = 4.35
soil organic carbon	0.0914587	0.0482603	1.9	0.0688	-0.0075633	0.1904806	0.0847	F(1 27) = 3.59
city total population	0.4424189	0.2377119	1.86	0.0733	-0.0445119	0.9293497	0.0783	F(1 28) = 3.46
city latitude	-0.419284	0.2623106	-1.6	0.1204	-0.9549938	0.1164258	0.0478	F(1 30) = 2.55
city longitude	-0.1447426	0.0927649	-1.56	0.1292	-0.3341938	0.0447085	0.0442	F(1 30) = 2.43
city ave june temp	-1.640685	1.226618	-1.34	0.1922	-4.157497	0.876127	0.0274	F(1 27) = 1.79
GHI	10.52793	8.857782	1.19	0.2439	-7.562078	28.61793	0.0131	F(1 30) = 1.41
soil ph	-1.066757	0.9273348	-1.15	0.2591	-2.960627	0.8271134	0.0103	F(1 30) = 1.32
greenfraction	42.00448	39.32722	1.07	0.294	-38.31242	122.3214	0.0045	F(1 30) = 1.14
no2	0.346106	0.3309954	1.05	0.3123	-0.3593939	1.051606	0.0058	F(1 15) = 1.09
soil total carbon	0.0317239	0.0328186	0.97	0.3423	-0.0356143	0.0990622	-0.0023	F(1 27) = 0.93
soil total phosphorus	-0.0284217	0.0295571	-0.96	0.3439	-0.0887854	0.0319421	-0.0024	F(1 30) = 0.92
RH	0.4480738	0.4834193	0.93	0.364	-0.5544765	1.450624	-0.0062	F(1 22) = 0.86
DHI	14.55513	16.50379	0.88	0.3848	-19.1501	48.26035	-0.0072	F(1 30) = 0.78
soil moisture(10-40cm)	0.7418483	0.8433093	0.88	0.3865	-0.9855926	2.469289	-0.0079	F(1 28) = 0.77
soil moisture(0-10cm)	1.958984	2.331778	0.84	0.408	-2.817446	6.735414	-0.0102	F(1 28) = 0.71
pm25	0.3535172	0.4411036	0.8	0.4292	-0.5473365	1.254371	-0.0117	F(1 30) = 0.64
fire carbon emissions	-0.1096553	0.1785517	-0.61	0.5438	-0.4743066	0.2549959	-0.0205	F(1 30) = 0.38
pet	-4.568013	7.636297	-0.6	0.5542	-20.16341	11.02739	-0.0212	F(1 30) = 0.36
co	-0.1026858	0.2127566	-0.48	0.6352	-0.5496709	0.3442993	-0.0421	F(1 18) = 0.23
o3	-0.2254151	0.545244	-0.41	0.6833	-1.356182	0.9053518	-0.0374	F(1 22) = 0.17
DNI	2.325526	5.991793	0.39	0.7007	-9.911348	14.5624	-0.0282	F(1 30) = 0.15
urban-rural temperature difference day	2.48147	7.529429	0.33	0.7447	-13.09434	18.05728	-0.0386	F(1 23) = 0.11
city population density	0.0004301	0.0016834	0.26	0.8003	-0.0030239	0.003884	-0.0345	F(1 27) = 0.07
PCI	-0.1799344	0.9370173	-0.19	0.849	-2.093579	1.73371	-0.0321	F(1 30) = 0.04
SDP	0.0318145	0.2220828	0.14	0.887	-0.421739	0.4853681	-0.0326	F(1 30) = 0.02
temperature	-0.1420808	1.134131	-0.13	0.9011	-2.458286	2.174124	-0.0328	F(1 30) = 0.02
vap	-0.0783774	1.150098	-0.07	0.9461	-2.427191	2.270436	-0.0332	F(1 30) = 0.00
soil tmp	0.05259	1.173718	0.04	0.9646	-2.351662	2.456842	-0.0356	F(1 28) = 0.00
wet	0.0370623	1.657394	0.02	0.9823	-3.347788	3.421912	-0.0333	F(1 30) = 0.00
precipitation	-0.0002419	0.0110025	-0.02	0.9826	-0.022712	0.0222281	-0.0333	F(1 30) = 0.00

	Estimate	Std. Error	t value	Pr(>t)	AdjR-squared
(Intercept)	36.31	29.93	1.213	0.235	-0.01294
main climateequatorial	21.57	34.55	0.624	0.538	
main climatesnow	9	36.65	0.246	0.808	
main climatewarm	36.38	31.39	1.159	0.256	
(Intercept)	40.818	42.745	0.955	0.348	-0.03337
dat2$vegetation2Forests	26.606	43.529	0.611	0.546	
dat2$vegetation2Grasslands	7.586	47.79	0.159	0.875	
(Intercept)	68.443	10.306	6.641	2.36e-07 ***	-0.02111
dat2$coastal city	-9.019	15.052	-0.599	0.554	
(Intercept)	55.53090909	20.24421315	2.743050998	0.011586487	-0.158924553
soil type2Andisols	6.08727273	33.05866164	0.184135486	0.855521006	
soil type2Entisols	15.98727274	30.36631973	0.526480419	0.603595183	
soil type2Inceptisols	29.59636364	25.80640948	1.146860963	0.263230251	
soil type2Mollisols	-21.78545455	37.87345484	-0.575216986	0.570729588	
soil type2Oxisols	-9.87636364	49.58799247	-0.199168451	0.843881207	
soil type2Spodosols	5.46909091	37.87345484	0.144404331	0.886439082	
soil type2Ultisols	4.696363637	27.41079419	0.171332637	0.86546067	
soil type2Vertisols	-26.74909091	49.58799247	-0.539426776	0.594776566	

Supplementary Table 10. Results of regression analysis, Shannon VS environment and demographic characteristics

Green = p<=0.05, after regression diagnosis

White = p>0.05

Shannon	Coef.	Std. Err.	t	P>t	[95% Conf.	Interval]	AdjR-squared			F
GDP	-0.0104301	0.0054012	-1.93	0.0633	-0.0214769	0.0006166	0.0834	F(1 29)	=	3.73
soil organic carbon	0.0012972	0.0007312	1.77	0.0873	-0.0002032	0.0027975	0.0712	F(1 27)	=	3.15
pm10	0.0221417	0.0121411	1.82	0.0932	-0.0043115	0.0485949	0.1518	F(1 12)	=	3.33
urban-rural temperature difference nig	-0.2283643	0.1324367	-1.72	0.0981	-0.5023305	0.0456018	0.076	F(1 23)	=	2.97
UVindex	0.0591165	0.0411825	1.44	0.1615	-0.0249894	0.1432225	0.0331	F(1 30)	=	2.06
soil total carbon	0.0007329	0.0005102	1.44	0.1623	-0.0003138	0.0017797	0.0366	F(1 27)	=	2.06
soil organic carbon density	0.0233231	0.017066	1.37	0.183	-0.0116935	0.0583397	0.0301	F(1 27)	=	1.87
greenfraction	0.828546	0.6217308	1.33	0.1927	-0.4411976	2.09829	0.0244	F(1 30)	=	1.78
no2	0.0129563	0.0101121	1.28	0.2196	-0.0085973	0.0345098	0.0386	F(1 15)	=	1.64
city total population	0.0045072	0.0039345	1.15	0.2617	-0.0035522	0.0125665	0.0107	F(1 28)	=	1.31
city latitude	-0.0038192	0.0043077	-0.89	0.3823	-0.0126166	0.0049782	-0.0069	F(1 30)	=	0.79
DHI	0.2323564	0.2635609	0.88	0.385	-0.3059069	0.7706197	-0.0072	F(1 30)	=	0.78
soil ph	-0.0130795	0.0149426	-0.88	0.3884	-0.0435965	0.0174374	-0.0076	F(1 30)	=	0.77
city longitude	-0.0012966	0.0015221	-0.85	0.401	-0.0044051	0.0018118	-0.0089	F(1 30)	=	0.73
RH	0.011257	0.0140724	0.8	0.4323	-0.0179274	0.0404413	-0.0159	F(1 22)	=	0.64
PCI	-0.0104073	0.0148519	-0.7	0.4889	-0.040739	0.0199243	-0.0167	F(1 30)	=	0.49
soil total phosphorus	-0.0003179	0.0004757	-0.67	0.509	-0.0012895	0.0006536	-0.0182	F(1 30)	=	0.45
pet	-0.078061	0.1218429	-0.64	0.5266	-0.3268975	0.1707754	-0.0194	F(1 30)	=	0.41
GHI	0.0903615	0.143804	0.63	0.5345	-0.2033254	0.3840483	-0.0199	F(1 30)	=	0.39
pm25	0.0040762	0.0070802	0.58	0.5691	-0.0103836	0.018536	-0.022	F(1 30)	=	0.33
soil moisture(10-40cm)	0.0069314	0.0133906	0.52	0.6088	-0.0204981	0.0343608	-0.0259	F(1 28)	=	0.27
wet	0.0135601	0.0263521	0.51	0.6106	-0.040258	0.0673782	-0.0243	F(1 30)	=	0.26
city population density	0.0000135	0.0000268	0.5	0.6185	-0.0000414	0.0000684	-0.0274	F(1 27)	=	0.25
precipitation	0.0000854	0.000175	0.49	0.6291	-0.000272	0.0004428	-0.0252	F(1 30)	=	0.24
soil moisture(0-10cm)	0.0175466	0.03701	0.47	0.6391	-0.058265	0.0933581	-0.0275	F(1 28)	=	0.22
SDP	0.0016682	0.0035347	0.47	0.6404	-0.0055506	0.0088869	-0.0257	F(1 30)	=	0.22
fire carbon emissions	-0.000894	0.0028646	-0.31	0.7571	-0.0067443	0.0049564	-0.03	F(1 30)	=	0.10
urban-rural temperature difference da	0.0337549	0.1096091	0.31	0.7609	-0.1929888	0.2604985	-0.0392	F(1 23)	=	0.09
o3	0.0046663	0.0158258	0.29	0.7709	-0.0281544	0.0374871	-0.0413	F(1 22)	=	0.09
soil tmp	0.0051614	0.0184474	0.28	0.7817	-0.0326264	0.0429492	-0.0328	F(1 28)	=	0.08
co	0.0016359	0.0061811	0.26	0.7943	-0.01135	0.0146218	-0.0515	F(1 18)	=	0.07
DNI	-0.0199175	0.0958574	-0.21	0.8368	-0.2156844	0.1758494	-0.0318	F(1 30)	=	0.04
vap	0.0023182	0.0183631	0.13	0.9004	-0.0351843	0.0398207	-0.0328	F(1 30)	=	0.02
city ave june temp	-0.0016525	0.0202065	-0.08	0.9354	-0.0431127	0.0398078	-0.0368	F(1 27)	=	0.01
temperature	0.0014288	0.0181145	0.08	0.9377	-0.0355659	0.0384234	-0.0331	F(1 30)	=	0.01

	Estimate	Std. Error	t value	Pr(>t)	AdjR-squared
(Intercept)	4.7845	0.4565	10.48	3.39E-11	0.07553
dat2$main climateequatorial	0.6907	0.5272	1.31	0.2008	
dat2$main climatewarm	0.9468	0.4788	1.977	0.0579	
dat2$main climatesnow	0.4315	0.5592	0.772	0.4467	
(Intercept)	5.0081	0.6753	7.417	3.58E-08	-0.0112
dat2$vegetation2Forests	0.6153	0.6876	0.895	0.378	
dat2$vegetation2Grasslands	0.2594	0.755	0.344	0.734	
(Intercept)	5.5599363	0.165557	33.583	<2e-16	-0.03333
dat2$coastal city	-0.0004461	0.2418115	-0.002	0.999	
(Intercept)	5.493058909	0.301874754	18.19648327	3.75399E-15	-0.010461955
soil type2Andisols	0.173911394	0.492959409	0.352790495	0.72745812	
soil type2Entisols	0.023654727	0.452812131	0.052239606	0.958788846	
soil type2Inceptisols	0.417070637	0.384816315	1.083817448	0.289676972	
soil type2Mollisols	-0.701550727	0.564755951	-1.242219272	0.226673171	
soil type2Oxisols	-0.191417091	0.739439113	-0.258867955	0.798039834	
soil type2Spodosols	0.205702	0.564755951	0.364231665	0.719012202	
soil type2Ultisols	0.062852	0.408740349	0.153769992	0.879132339	
soil type2Vertisols	-1.013531636	0.739439113	-1.370676257	0.183705277	

Supplementary Table 11. Results of regression analysis, Fisher alpha VS environment and demographic characteristics

Green = p<=0.05, after regression diagnosis

White = p>0.05

fisher alpha	Coef.	Std. Err.	t	P>|t|	[95% Conf.	Interval]	F	Adj R-squared
UVindex	2.571007	0.8093049	3.18	0.0034	0.9181855	4.223828	F(1 30)=10.09	0.2268
GDP	-0.338	0.108	-3.130	0.004	-0.559	-0.117	F(1 30)=9.77	0.221
pm10	0.683	0.201	3.400	0.005	0.249	1.118	F(1 13)=11.55	0.430
soil organic carbon density	0.9694457	0.3410664	2.84	0.008	0.2728951	1.665996	F(1 30)=8.08	0.1859
elevation	0.0112626	0.0040565	2.78	0.0095	0.0029661	0.019559	F(1 29)=7.71	0.1828
urban-rural temperature difference night	-7.074324	3.005855	-2.35	0.0275	-13.29241	-0.856238	F(1 23)=5.54	0.159
city latitude	-0.1879009	0.0895496	-2.1	0.0444	-0.3707856	-0.0050161	F(1 30)=4.40	0.0989
GHI	6.193248	2.973166	2.08	0.0459	0.1212321	12.26526	F(1 30)=4.34	0.0972
city longitude	-0.0578915	0.0321579	-1.8	0.0819	-0.1235668	0.0077838	F(1 30)=3.24	0.0674
city total population	0.1513654	0.083926	1.8	0.0821	-0.0205492	0.32328	F(1 28)=3.25	0.0721
pm25	0.2541577	0.1494107	1.7	0.0993	-0.0509796	0.5592951	F(1 30)=2.89	0.0576
soil organic carbon	0.024467	0.0174365	1.4	0.172	-0.0113098	0.0602438	F(1 27)=1.97	0.0334
DHI	7.567247	5.70148	1.33	0.1944	-4.076729	19.21122	F(1 30)=1.76	0.024
greenness fraction	17.89613	13.67669	1.31	0.2006	-10.03541	45.82767	F(1 30)=1.71	0.0225
city ave june temp	-0.5613447	0.4340307	-1.29	0.2069	-1.451902	0.3292127	F(1 27)=1.67	0.0235
soil moisture(10-40cm)	0.263664	0.2970147	0.89	0.3823	-0.344743	0.872071	F(1 28)=0.79	-0.0074
standard deviation of monthly precipitatio	0.062262	0.0771319	0.81	0.4259	-0.0952623	0.2197862	F(1 30)=0.65	-0.0114
DNI	1.615908	2.087303	0.77	0.4449	-2.646933	5.878748	F(1 30)=0.60	-0.0131
wet	-0.4403115	0.5760732	-0.76	0.4506	-1.61681	0.7361869	F(1 30)=0.58	-0.0136
RH	-0.1323453	0.1817136	-0.73	0.4741	-0.5091963	0.2445057	F(1 22)=0.53	-0.0208
soil total carbon	0.0077352	0.0111504	0.69	0.4932	-0.015037	0.0305074	F(1 30)=0.48	-0.017
PCI	0.2153991	0.3266829	0.66	0.5147	-0.4517764	0.8825747	F(1 30)=0.43	-0.0186
soil moisture(0-10cm)	0.5438425	0.8253723	0.66	0.5153	-1.146856	2.234541	F(1 28)=0.43	-0.0199
Soil PH	-0.2148762	0.3302194	-0.65	0.5202	-0.8892742	0.4595218	F(1 30)=0.42	-0.019
soil total phosphorus	0.0078613	0.012425	0.63	0.5317	-0.0175141	0.0332366	F(1 30)=0.40	-0.0197
soil tmp	0.2491625	0.410812	0.61	0.5491	-0.5923477	1.090673	F(1 28)=0.37	-0.0223
urban-rural temperature difference day	-1.477212	2.594881	-0.57	0.5747	-6.845132	3.890708	F(1 23)=0.32	-0.029
temperature	0.2162926	0.3961559	0.55	0.5891	-0.5927657	1.025351	F(1 30)=0.30	-0.0232
fire carbon emissions	-0.0274917	0.062854	-0.44	0.665	-0.1558567	0.1008733	F(1 30)=0.19	-0.0268
o3	-0.0851279	0.2034553	-0.42	0.6797	-0.5070683	0.3368125	F(1 22)=0.18	-0.0372
vapour	0.1627108	0.4025552	0.4	0.6889	-0.6594166	0.9848383	F(1 30)=0.16	-0.0277
co	-0.0102142	0.0635099	-0.16	0.874	-0.1436436	0.1232151	F(1 18)=0.03	-0.054
no2	-0.0201717	0.1342387	-0.15	0.8826	-0.3062948	0.2659513	F(1 15)=0.02	-0.0651
city population density	0.000057	0.0005951	0.1	0.9244	-0.0011639	0.001278	F(1 27)=0.01	-0.0367
precipitation	-0.0003078	0.0038609	-0.08	0.937	-0.0081927	0.0075771	F(1 30)=0.01	-0.0331
pet	0.1285362	2.695737	0.05	0.9623	-5.376892	5.633965	F(1 30)=0.00	-0.0333

Supplementary Table 12. Results of regression analysis, AMR VS environment and demographic characteristics

Green = p<=0.05, after regression diagnosis
White = p>0.05

Aminoglycosides prevalence	Coef.	Std. Err.	t	P>t	[95% Conf.	Interval]			F	AdjR-squared
city longitude	-0.0004237	0.0005404	-0.78	0.4391	-0.0015274	0.0006799	F(1 30)	=	0.61	-0.0126
city latitude	-0.002721	0.0014646	-1.86	0.073	-0.005712	0.0002701	F(1 30)	=	3.45	0.0733
city total population	0.0025404	0.0013284	1.91	0.0661	-0.0001808	0.0052615	F(1 28)	=	3.66	0.0839
city population density	-9.47E-06	9.12E-06	-1.04	0.3084	-0.0000282	0.00000925	F(1 27)	=	1.08	0.0028
city ave june temp	-0.0002252	0.0069894	-0.03	0.9745	-0.0145662	0.0141159	F(1 27)	=	0.00	-0.037
pm25	0.0047913	0.0023666	2.02	0.0519	-0.0000419	0.0096246	F(1 30)	=	4.10	0.0909
pm10	0.0031543	0.0017072	1.85	0.0894	-0.0005653	0.006874	F(1 12)	=	3.41	0.1566
o3	-0.0105584	0.0046607	-2.27	0.0337	-0.0202241	-0.0008927	F(1 22)	=	5.13	0.1523
no2	-0.0005781	0.0012968	-0.45	0.6621	-0.0033421	0.0021859	F(1 15)	=	0.20	-0.0527
co	-0.0012917	0.0016155	-0.8	0.4344	-0.0046856	0.0021023	F(1 18)	=	0.64	-0.0194
soil ph	-0.0023369	0.0053459	-0.44	0.6651	-0.0132548	0.008581	F(1 30)	=	0.19	-0.0268
soil organic carbon density	0.0123823	0.0057699	2.15	0.0401	0.0005986	0.0241661	F(1 30)	=	4.61	0.1042
soil organic carbon	0.00028	0.000281	1	0.3279	-0.0002966	0.0008566	F(1 27)	=	0.99	-0.0003
soil total carbon	-0.0000627	0.0001811	-0.35	0.7318	-0.0004343	0.0003089	F(1 27)	=	0.12	-0.0325
soil total phosphorus	-0.0000243	0.0001698	-0.14	0.8869	-0.0003711	0.0003224	F(1 30)	=	0.02	-0.0326
soil moisture(0-10cm)	-0.0117639	0.0129951	-0.91	0.3731	-0.0383832	0.0148554	F(1 28)	=	0.82	-0.0063
soil moisture(10-40cm)	-0.0018438	0.004761	-0.39	0.7015	-0.0115962	0.0079087	F(1 28)	=	0.15	-0.0302
soil tmp	0.012176	0.0061372	1.98	0.0571	-0.0003954	0.0247474	F(1 28)	=	3.94	0.0919
precipitation	0.0000924	0.0000599	1.54	0.1336	-0.00003	0.0002148	F(1 30)	=	2.38	0.0425
SDP	0.001916	0.0012077	1.59	0.1231	-0.0005505	0.0043825	F(1 30)	=	2.52	0.0466
PCI	-0.0041396	0.0052524	-0.79	0.4368	-0.0148664	0.0065873	F(1 30)	=	0.62	-0.0124
urban-rural temperature difference day	0.00832	0.0421522	0.2	0.8453	-0.0788785	0.0955186	F(1 23)	=	0.04	-0.0417
urban-rural temperature difference night	-0.090233	0.0342639	-2.63	0.0149	-0.1611133	-0.0193527	F(1 23)	=	6.94	0.1983
greenness fraction	0.2138523	0.2233866	0.96	0.3461	-0.242364	0.6700686	F(1 30)	=	0.92	-0.0027
DHI	0.2986105	0.0773222	3.86	0.0006	0.1406975	0.4565234	F(1 30)	=	14.91	0.3098
DNI	-0.0521908	0.0326342	-1.6	0.1202	-0.1188386	0.014457	F(1 30)	=	2.56	0.0478
GHI	0.0598181	0.0501232	1.19	0.2421	-0.0425471	0.1621833	F(1 30)	=	1.42	0.0135
UVindex	0.047198	0.0123855	3.81	0.0006	0.0219035	0.0724925	F(1 30)	=	14.52	0.3037
wet	0.0060254	0.0093157	0.65	0.5227	-0.0129999	0.0250506	F(1 30)	=	0.42	-0.0191
pet	-0.0044423	0.0434686	-0.1	0.9193	-0.0932171	0.0843325	F(1 30)	=	0.01	-0.033
RH	0.0066434	0.0044392	1.5	0.1487	-0.0025629	0.0158496	F(1 22)	=	2.24	0.0511
vap	0.0153306	0.0058773	2.61	0.014	0.0033276	0.0273335	F(1 30)	=	6.80	0.1577
temperature	0.0128475	0.0059767	2.15	0.0398	0.0006413	0.0250536	F(1 30)	=	4.62	0.1046
fire carbon emissions	-0.0001795	0.0010164	-0.18	0.861	-0.0022552	0.0018962	F(1 30)	=	0.03	-0.0323
GDP	-0.0069077	0.0015933	-4.34	0.0002	-0.0101665	-0.003649	F(1 29)	=	18.80	0.3723
anti consum	-0.0119268	0.0057291	-2.08	0.046	-0.0236271	-0.0002265	F(1 30)	=	4.33	0.0971
coastal city	-0.1301175	0.0823409	-1.58	0.1245	-0.29828	0.038045	F(1 30)	=	2.50	0.0461
main clima~l	0.3042602	0.1913588	1.59	0.1231	-0.0877206	0.6962409	F(3 28)	=	1.32	0.0302
main climate snow	0.0629287	0.2029667	0.31	0.7588	-0.3528297	0.4786871				
main climate warm	0.144579	0.1738103	0.83	0.4125	-0.2114552	0.5006132				
soil type Andisols	-0.2370938	0.1828099	-1.3	0.2075	-0.615265	0.1410774	F(8 23)	=	0.63	-0.1064
soil type Entisols	-0.1900452	0.1679217	-1.13	0.2694	-0.5374176	0.1573273				
soil type Inceptisols	-0.0195232	0.142706	-0.14	0.8924	-0.314733	0.2756866				
soil type Gelisols	-0.155131	0.2094351	-0.74	0.4664	-0.5883805	0.2781185				
soil type Oxisols	-0.1395872	0.2742149	-0.51	0.6156	-0.7068439	0.4276696				
soil type Spodosols	-0.2590169	0.2094351	-1.24	0.2287	-0.6922664	0.1742327				
soil type Ultisols	-0.0456679	0.151578	-0.3	0.7659	-0.3592309	0.2678951				
soil type Vertisols	-0.3190743	0.2742149	-1.16	0.2565	-0.8863311	0.2481824				
vegetation Forests	0.1500394	0.2231686	0.67	0.5067	-0.3063916	0.6064704	F(2 29)	=	3.78	0.1521
vegetation Grasslands	0.4569613	0.245014	1.87	0.0723	-0.0441486	0.9580712				
Europe	-0.1304392	0.0896075	-1.46	0.1579	-0.3149893	0.0541109	F(6 25)	=	4.35	0.3935
the Middle East	-0.2143813	0.1981296	-1.08	0.2896	-0.6224369	0.1936743				
North America	-0.132949	0.1085201	-1.23	0.232	-0.3564502	0.0905523				
Oceania	-0.2084289	0.1485972	-1.4	0.173	-0.5144706	0.0976128				
South America	0.2647762	0.1161638	2.28	0.0314	0.0255324	0.50402				
Sub Saharan Africa	0.3452093	0.1485972	2.32	0.0286	0.0391676	0.651251				
**Elfamycins prevalence**	**Coef.**	**Std. Err.**	**t**	**P>t**	**[95% Conf.**	**Interval]**			**F**	**AdjR-squared**
city longitude	-0.0007642	0.0005393	-1.42	0.1668	-0.0018656	0.0003371	F(1 30)	=	2.01	0.0315
city latitude	-0.0024934	0.001511	-1.65	0.1093	-0.0055792	0.0005925	F(1 30)	=	2.72	0.0527
city total population	0.0015774	0.001397	1.13	0.2684	-0.0012842	0.004439	F(1 28)	=	1.27	0.0094
city population density	6.13E-07	9.47E-06	0.06	0.9489	-0.0000188	0.00002	F(1 27)	=	0.00	-0.0369
city ave june temp	-0.0037956	0.0070757	-0.54	0.5961	-0.0183137	0.0107224	F(1 27)	=	0.29	-0.0261
pm25	0.0038561	0.0024764	1.56	0.1299	-0.0012015	0.0089137	F(1 30)	=	2.42	0.0439
pm10	0.0030875	0.0039118	0.79	0.4453	-0.0054356	0.0116106	F(1 12)	=	0.62	-0.0299
o3	-0.0046141	0.005305	-0.87	0.3938	-0.015616	0.0063879	F(1 22)	=	0.76	-0.0107
no2	0.0005541	0.0028917	0.19	0.8506	-0.0056095	0.0067178	F(1 15)	=	0.04	-0.0641
co	-0.00207	0.0021625	-0.96	0.3511	-0.0066132	0.0024733	F(1 18)	=	0.92	-0.0044
soil ph	-0.0070004	0.005321	-1.32	0.1983	-0.0178674	0.0038665	F(1 30)	=	1.73	0.023
soil organic carbon density	0.0094914	0.0060813	1.56	0.1291	-0.0029283	0.0219111	F(1 30)	=	2.44	0.0443
soil organic carbon	0.0001972	0.0002953	0.67	0.51	-0.0004088	0.0008031	F(1 27)	=	0.45	-0.0202
soil total carbon	0.0000177	0.0001828	0.1	0.9236	-0.0003574	0.0003928	F(1 27)	=	0.01	-0.0367
soil total phosphorus	-0.0000521	0.000173	-0.3	0.7655	-0.0004055	0.0003013	F(1 30)	=	0.09	-0.0302
soil moisture(0-10cm)	-0.0134004	0.0134071	-1	0.3261	-0.0408637	0.0140629	F(1 28)	=	1.00	0
soil moisture(10-40cm)	-0.002898	0.00491	-0.59	0.5598	-0.0129556	0.0071596	F(1 28)	=	0.35	-0.023
soil tmp	0.0105299	0.0064847	1.62	0.1156	-0.0027535	0.0238133	F(1 28)	=	2.64	0.0534
precipitation	-9.48E-06	0.0000635	-0.15	0.8824	-0.0001392	0.0001202	F(1 30)	=	0.02	-0.0326
SDP	-0.0001798	0.0012826	-0.14	0.8894	-0.0027992	0.0024395	F(1 30)	=	0.02	-0.0327
PCI	-0.0023285	0.005398	-0.43	0.6693	-0.0133528	0.0086958	F(1 30)	=	0.19	-0.027
urban-rural temperature difference day	0.0169377	0.0438408	0.39	0.7028	-0.073754	0.1076293	F(1 23)	=	0.15	-0.0368
urban-rural temperature difference nigh	-0.0501808	0.0393868	-1.27	0.2154	-0.1316586	0.031297	F(1 23)	=	1.62	0.0253
greenness fraction	0.1906729	0.2287655	0.83	0.4112	-0.2765286	0.6578744	F(1 30)	=	0.69	-0.0099
DHI	0.2138958	0.0882885	2.42	0.0217	0.0335866	0.394205	F(1 30)	=	5.87	0.1358
DNI	-0.0065982	0.0346694	-0.19	0.8503	-0.0774026	0.0642062	F(1 30)	=	0.04	-0.0321
GHI	0.08391	0.0500537	1.68	0.1041	-0.0183134	0.1861333	F(1 30)	=	2.81	0.0552
UVindex	0.0392898	0.0136228	2.88	0.0072	0.0114682	0.0671113	F(1 30)	=	8.32	0.191
wet	-0.0051056	0.0095263	-0.54	0.5959	-0.0245609	0.0143497	F(1 30)	=	0.29	-0.0235
pet	0.0092296	0.0443311	0.21	0.8365	-0.0813066	0.0997657	F(1 30)	=	0.04	-0.0318
RH	0.003983	0.0047825	0.83	0.4139	-0.0059353	0.0139014	F(1 22)	=	0.69	-0.0135
vap	0.0099654	0.0063885	1.56	0.1293	-0.0030817	0.0230124	F(1 30)	=	2.43	0.0442
temperature	0.0096857	0.0063083	1.54	0.1352	-0.0031976	0.0225691	F(1 30)	=	2.36	0.042
fire carbon emissions	-0.0007051	0.0010296	-0.68	0.4987	-0.0028079	0.0013976	F(1 30)	=	0.47	-0.0174
GDP	-0.0054163	0.0016301	-3.32	0.0024	-0.0087502	-0.0020824	F(1 29)	=	11.04	0.2508
anti consum	-0.008597	0.0060538	-1.42	0.1659	-0.0209605	0.0037665	F(1 30)	=	2.02	0.0318
coastal city	-0.043587	0.0870847	-0.5	0.6204	-0.2214377	0.1342637	F(1 30)	=	0.25	-0.0248
main clima~l	0.2621567	0.1975393	1.33	0.1952	-0.1424841	0.6667975	F(3 28)	=	1.08	0.0074
main climate snow	0.0194918	0.209522	0.09	0.9265	-0.4096946	0.4486783				
main climate warm	0.1556947	0.1794239	0.87	0.3929	-0.2118386	0.523228				
soil type Andisols	-0.1438864	0.1819034	-0.79	0.437	-0.5201823	0.2324095	F(8 23)	=	0.81	-0.0521
soil type Entisols	-0.1515348	0.1670889	-0.91	0.3739	-0.4971846	0.194115				
soil type Inceptisols	0.0550131	0.1419983	0.39	0.702	-0.2387328	0.348759				
soil type Gelisols	-0.2010034	0.2083965	-0.96	0.3448	-0.6321045	0.2300976				
soil type Oxisols	-0.2821162	0.2728551	-1.03	0.3119	-0.84656	0.2823276				
soil type Spodosols	-0.2795963	0.2083965	-1.34	0.1928	-0.7106974	0.1515047				
soil type Ultisols	-0.1106915	0.1508263	-0.73	0.4704	-0.4226995	0.2013166				
soil type Vertisols	-0.2713936	0.2728551	-0.99	0.3303	-0.8358374	0.2930502				
vegetation Forests	0.0981711	0.2491865	0.39	0.6965	-0.4114726	0.6078148	F(2 29)	=	0.77	-0.0153
vegetation Grasslands	0.2481225	0.2735788	0.91	0.3719	-0.311409	0.807654				
Europe	0.0671889	0.0983851	0.68	0.5009	-0.135439	0.2698167	F(6 25)	=	3.19	0.2978
the Middle East	-0.0167189	0.2175376	-0.08	0.9394	-0.4647459	0.4313081				
North America	-0.0197425	0.1191502	-0.17	0.8697	-0.265137	0.225652				
Oceania	-0.0071951	0.1631532	-0.04	0.9652	-0.3432153	0.3288251				
South America	0.4031367	0.1275427	3.16	0.0041	0.1404576	0.6658158				
Sub Saharan Africa	0.4431504	0.1631532	2.72	0.0118	0.1071302	0.7791707				
**MLS prevalence**	**Coef.**	**Std. Err.**	**t**	**P>t**	**[95% Conf.**	**Interval]**			**F**	**AdjR-squared**
city longitude	-0.0009264	0.0005901	-1.57	0.1269	-0.0021315	0.0002786	F(1 30)	=	2.47	0.0451
city latitude	0.0004098	0.0017373	0.24	0.8151	-0.0031383	0.003958	F(1 30)	=	0.06	-0.0314
city total population	0.0019418	0.0015164	1.28	0.2109	-0.0011645	0.005048	F(1 28)	=	1.64	0.0216
city population density	5.63E-07	0.0000105	0.05	0.9575	-0.0000209	0.000022	F(1 27)	=	0.00	-0.0369
city ave june temp	-0.0006799	0.0078638	-0.09	0.9317	-0.0168152	0.0154553	F(1 27)	=	0.01	-0.0367
pm25	0.001365	0.0028261	0.48	0.6326	-0.0044066	0.0071366	F(1 30)	=	0.23	-0.0254
pm10	0.0054234	0.0050195	1.08	0.3012	-0.0055132	0.01636	F(1 12)	=	1.17	0.0127
o3	0.0033416	0.0077821	0.43	0.6718	-0.0127975	0.0194806	F(1 22)	=	0.18	-0.0368
no2	0.0021272	0.0038582	0.55	0.5895	-0.0060964	0.0103508	F(1 15)	=	0.30	-0.0455
co	0.0015773	0.0028215	0.56	0.583	-0.0043504	0.007505	F(1 18)	=	0.31	-0.0375
soil ph	-0.002373	0.0060147	-0.39	0.696	-0.0146566	0.0099106	F(1 30)	=	0.16	-0.028
soil organic carbon density	0.006674	0.0068607	0.97	0.3384	-0.0073374	0.0206854	F(1 30)	=	0.95	-0.0017
soil organic carbon	0.0003693	0.0003162	1.17	0.2531	-0.0002796	0.0010181	F(1 27)	=	1.36	0.0128

soil total carbon	0.0003468	0.0001976	1.75	0.0907	-0.0000588	0.0007523	F(1 27)	=	3.08	0.0691
soil total phosphorus	0.0000876	0.0001903	0.46	0.6485	-0.000301	0.0004763	F(1 30)	=	0.21	-0.0261
soil moisture(0-10cm)	-0.0039161	0.0149366	-0.26	0.7951	-0.0345123	0.02668	F(1 28)	=	0.07	-0.0332
soil moisture(10-40cm)	-0.0026624	0.0053916	-0.49	0.6253	-0.0137065	0.0083817	F(1 28)	=	0.24	-0.0268
soil tmp	-0.0021125	0.0074241	-0.28	0.7781	-0.01732	0.0130951	F(1 28)	=	0.08	-0.0327
precipitation	-0.0000583	0.0000692	-0.84	0.4066	-0.0001996	0.0000831	F(1 30)	=	0.71	-0.0095
SDP	-0.0010764	0.0014001	-0.77	0.448	-0.0039358	0.0017829	F(1 30)	=	0.59	-0.0134
PCI	-0.0038154	0.005926	-0.64	0.5246	-0.0159178	0.0082871	F(1 30)	=	0.41	-0.0192
urban-rural temperature difference day	-0.009655	0.0441136	-0.22	0.8287	-0.100911	0.0816009	F(1 23)	=	0.05	-0.0413
urban-rural temperature difference nigh	-0.0713513	0.0381159	-1.87	0.074	-0.1502001	0.0074975	F(1 23)	=	3.50	0.0945
greenness fraction	0.3164111	0.2483595	1.27	0.2124	-0.1908068	0.823629	F(1 30)	=	1.62	0.0197
DHI	0.0278914	0.1062597	0.26	0.7947	-0.1891197	0.2449026	F(1 30)	=	0.07	-0.031
DNI	-0.0195241	0.0380604	-0.51	0.6117	-0.0972537	0.0582056	F(1 30)	=	0.26	-0.0243
GHI	-0.0121414	0.0576396	-0.21	0.8346	-0.129857	0.1055743	F(1 30)	=	0.04	-0.0318
UVindex	0.0063091	0.0169265	0.37	0.712	-0.0282593	0.0408776	F(1 30)	=	0.14	-0.0286
wet	-0.0008297	0.0105465	-0.08	0.9378	-0.0223686	0.0207091	F(1 30)	=	0.01	-0.0331
pet	-0.0438002	0.0482273	-0.91	0.371	-0.1422934	0.0546931	F(1 30)	=	0.82	-0.0057
RH	0.0001147	0.0070352	0.02	0.9871	-0.0144755	0.0147048	F(1 22)	=	0.00	-0.0454
vap	-0.0025223	0.0073052	-0.35	0.7323	-0.0174416	0.0123969	F(1 30)	=	0.12	-0.0292
temperature	-0.0021261	0.007209	-0.29	0.7701	-0.0168488	0.0125966	F(1 30)	=	0.09	-0.0303
fire carbon emissions	-0.0008297	0.0011333	-0.73	0.4698	-0.0031442	0.0014849	F(1 30)	=	0.54	-0.0152
GDP	-0.0036783	0.002164	-1.7	0.0999	-0.0081041	0.0007476	F(1 29)	=	2.89	0.0592
anti consum	0.0010936	0.0068886	0.16	0.8749	-0.0129748	0.0151621	F(1 30)	=	0.03	-0.0325
coastal city	-0.0695124	0.0955232	-0.73	0.4724	-0.2645967	0.125572	F(1 30)	=	0.53	-0.0154
main clima~l	0.1248862	0.2101701	0.59	0.5571	-0.3056277	0.5554001	F(3 28)	=	1.83	0.0747
main climate snow	0.0042832	0.222919	0.02	0.9848	-0.4523458	0.4609121				
main climate warm	0.2704211	0.1908965	1.42	0.1676	-0.1206126	0.6614547				
soil type Andisols	0.2084043	0.1855469	1.12	0.2729	-0.1754288	0.5922374	F(8 23)	=	1.42	0.0985
soil type Entisols	0.0088182	0.1704357	0.05	0.9592	-0.343755	0.3613914				
soil type Inceptisols	0.2867371	0.1448425	1.98	0.0598	-0.0128926	0.5863667				
soil type Gelisols	-0.0639693	0.2125707	-0.3	0.7662	-0.5037054	0.3757667				
soil type Oxisols	-0.1312561	0.2783204	-0.47	0.6417	-0.7070057	0.4444936				
soil type Spodosols	-0.17447	0.2125707	-0.82	0.4202	-0.6142061	0.265266				
soil type Ultisols	0.0687956	0.1538474	0.45	0.6589	-0.249462	0.3870532				
soil type Vertisols	-0.1941931	0.2783204	-0.7	0.4923	-0.7699428	0.3815565				
vegetation Forests	0.1734625	0.2798668	0.62	0.5402	-0.3989295	0.7458545	F(2 29)	=	0.20	-0.0547
vegetation Grasslands	0.1797243	0.3072623	0.58	0.5631	-0.4486977	0.8081464				
Europe	0.1768173	0.1222692	1.45	0.1606	-0.0750008	0.4286354	F(6 25)	=	1.62	0.1069
the Middle East	-0.0795129	0.2703473	-0.29	0.7711	-0.6363036	0.4772779				
North America	0.0014728	0.1480753	0.01	0.9921	-0.3034941	0.3064396				
Oceania	-0.2009414	0.2027605	-0.99	0.3312	-0.6185345	0.2166516				
South America	0.3331083	0.1585052	2.1	0.0458	0.0066608	0.6595558				
Sub Saharan Africa	0.0272903	0.2027605	0.13	0.894	-0.3903028	0.4448833				
Multi-drug resistance prevalence	Coef.	Std. Err.	t	P>t	[95% Conf.	Interval]	F			AdjR-squared
city longitude	-0.0009242	0.0004838	-1.91	0.0657	-0.0019123	0.0000639	F(1 30)	=	3.65	0.0787
city latitude	-0.0014872	0.001426	-1.04	0.3053	-0.0043996	0.0014252	F(1 30)	=	1.09	0.0028
city total population	0.0023047	0.0012371	1.86	0.073	-0.0002294	0.0048388	F(1 28)	=	3.47	0.0785
city population density	-9.51E-06	8.54E-06	-1.11	0.2757	-0.000027	0.00000802	F(1 27)	=	1.24	0.0084
city ave june temp	-0.01046	0.0062484	-1.67	0.1057	-0.0232807	0.0023608	F(1 27)	=	2.80	0.0605
pm25	0.0028103	0.0023121	1.22	0.2337	-0.0019116	0.0075322	F(1 30)	=	1.48	0.0152
pm10	0.0034699	0.0031308	1.11	0.2895	-0.0033516	0.0102913	F(1 12)	=	1.23	0.0173
o3	-0.0058371	0.0052617	-1.11	0.2793	-0.0167492	0.005075	F(1 22)	=	1.23	0.0099
no2	0.000596	0.0022799	0.26	0.7973	-0.0042634	0.0054554	F(1 15)	=	0.07	-0.0618
co	-0.0011913	0.001869	-0.64	0.5319	-0.005118	0.0027354	F(1 18)	=	0.41	-0.0323
soil ph	-0.0024805	0.0050136	-0.49	0.6244	-0.0127195	0.0077586	F(1 30)	=	0.24	-0.025
soil organic carbon density	0.0150318	0.0051287	2.93	0.0064	0.0045576	0.0255061	F(1 30)	=	8.59	0.1967
soil organic carbon	0.0003635	0.0002625	1.39	0.1774	-0.000175	0.000902	F(1 27)	=	1.92	0.0318
soil total carbon	0.0000483	0.0001636	0.3	0.7699	-0.0002874	0.0003841	F(1 27)	=	0.09	-0.0337
soil total phosphorus	-0.0000717	0.0001589	-0.45	0.6552	-0.0003961	0.0002528	F(1 30)	=	0.20	-0.0264
soil moisture(0-10cm)	-0.001787	0.0124986	-0.14	0.8873	-0.0273893	0.0238153	F(1 28)	=	0.02	-0.035
soil moisture(10-40cm)	0.0003705	0.0045267	0.08	0.9354	-0.0089021	0.009643	F(1 28)	=	0.01	-0.0355
soil tmp	0.0014273	0.0062101	0.23	0.8199	-0.0112935	0.0141481	F(1 28)	=	0.05	-0.0338
precipitation	-0.0000113	0.0000584	-0.19	0.8478	-0.0001306	0.000108	F(1 30)	=	0.04	-0.032
SDP	0.0001334	0.00118	0.11	0.9108	-0.0022765	0.0025432	F(1 30)	=	0.01	-0.0329
PCI	-0.0019581	0.0049682	-0.39	0.6963	-0.0121044	0.0081882	F(1 30)	=	0.16	-0.028
urban-rural temperature difference day	0.0031772	0.039596	0.08	0.9367	-0.0787334	0.0850879	F(1 23)	=	0.01	-0.0432
urban-rural temperature difference nigh	-0.0721536	0.0334672	-2.16	0.0418	-0.1413858	-0.0029215	F(1 23)	=	4.65	0.1319
greenness fraction	0.2551016	0.2077048	1.23	0.2289	-0.1690882	0.6792914	F(1 30)	=	1.51	0.0161
DHI	0.1187903	0.0861173	1.38	0.178	-0.0570847	0.2946652	F(1 30)	=	1.90	0.0283
DNI	-0.0231633	0.03163	-0.73	0.4697	-0.0877603	0.0414338	F(1 30)	=	0.54	-0.0152
GHI	0.0205635	0.0480059	0.43	0.6715	-0.0774776	0.1186047	F(1 30)	=	0.18	-0.0271
UVindex	0.0267416	0.0132946	2.01	0.0533	-0.0004096	0.0538927	F(1 30)	=	4.05	0.0895
wet	0.0002676	0.0088049	0.03	0.976	-0.0177144	0.0182497	F(1 30)	=	0.00	-0.0333
pet	-0.0292277	0.040459	-0.72	0.4756	-0.111856	0.0534005	F(1 30)	=	0.52	-0.0157
RH	0.0042762	0.0047815	0.89	0.3808	-0.0056399	0.0141924	F(1 22)	=	0.80	-0.0088
vap	0.0040904	0.0060646	0.67	0.5052	-0.0082951	0.016476	F(1 30)	=	0.45	-0.0179
temperature	0.0022679	0.0060125	0.38	0.7087	-0.0100112	0.014547	F(1 30)	=	0.14	-0.0285
fire carbon emissions	-0.0003685	0.0009521	-0.39	0.7015	-0.002313	0.001576	F(1 30)	=	0.15	-0.0282
GDP	-0.0036935	0.0017857	-2.07	0.0476	-0.0073456	-0.0000413	F(1 29)	=	4.28	0.0985
anti consum	-0.0099915	0.0054561	-1.83	0.077	-0.0211344	0.0011514	F(1 30)	=	3.35	0.0706
coastal city	-0.0738394	0.0793048	-0.93	0.3592	-0.2358014	0.0881227	F(1 30)	=	0.87	-0.0043
main clima~l	0.1273226	0.1901703	0.67	0.5086	-0.2622237	0.5168688	F(3 28)	=	0.17	-0.0871
main climate snow	0.0719559	0.2017061	0.36	0.724	-0.3412203	0.4851321				
main climate warm	0.0702932	0.1727308	0.41	0.6871	-0.2835297	0.4241162				
soil type Andisols	-0.0609727	0.1781388	-0.34	0.7353	-0.4294808	0.3075354	F(8 23)	=	0.37	-0.1923
soil type Entisols	0.0174918	0.1636309	0.11	0.9158	-0.3210045	0.3559881				
soil type Inceptisols	0.1284018	0.1390595	0.92	0.3654	-0.1592647	0.4160683				
soil type Gelisols	0.0085036	0.2040836	0.04	0.9671	-0.4136755	0.4306826				
soil type Oxisols	-0.137424	0.2672081	-0.51	0.612	-0.6901861	0.4153381				
soil type Spodosols	0.0725671	0.2040836	0.36	0.7254	-0.3496119	0.4947462				
soil type Ultisols	0.0455251	0.1477049	0.31	0.7607	-0.2600257	0.3510758				
soil type Vertisols	-0.163065	0.2672081	-0.61	0.5477	-0.7158272	0.3896971				
vegetation Forests	0.1056231	0.2255101	0.47	0.643	-0.3555968	0.5668429	F(2 29)	=	1.27	0.0173
vegetation Grasslands	0.2824027	0.2475847	1.14	0.2634	-0.2239648	0.7887703				
Europe	0.0218144	0.0925804	0.24	0.8156	-0.1688585	0.2124872	F(6 25)	=	2.87	0.2653
the Middle East	-0.0644197	0.2047029	-0.31	0.7556	-0.4860132	0.3571738				
North America	0.0017727	0.1121204	0.02	0.9875	-0.2291436	0.232689				
Oceania	-0.1358482	0.1535272	-0.88	0.3847	-0.4520434	0.1803469				
South America	0.3825841	0.1200177	3.19	0.0038	0.135403	0.6297652				
Sub Saharan Africa	0.238481	0.1535272	1.55	0.1329	-0.0777141	0.5546762				
Beta-lactams prevalence	Coef.	Std. Err.	t	P>t	[95% Conf.	Interval]	F			AdjR-squared
city longitude	-0.0007284	0.0004698	-1.55	0.1315	-0.0016878	0.0002311	F(1 30)	=	2.40	0.0433
city latitude	-0.0031326	0.0012594	-2.49	0.0187	-0.0057047	-0.0005605	F(1 30)	=	6.19	0.1433
city total population	0.0023973	0.0011909	2.01	0.0538	-0.0000422	0.0048368	F(1 28)	=	4.05	0.0952
city population density	-6.66E-06	8.40E-06	-0.79	0.4348	-0.0000239	0.0000106	F(1 27)	=	0.63	-0.0134
city ave june temp	-0.0081829	0.0061904	-1.32	0.1973	-0.0208844	0.0045187	F(1 27)	=	1.75	0.026
pm25	0.0032599	0.0021768	1.5	0.1447	-0.0011856	0.0077055	F(1 30)	=	2.24	0.0385
pm10	0.0042291	0.0029206	1.45	0.1732	-0.0021344	0.0105927	F(1 12)	=	2.10	0.0778
o3	-0.0096852	0.0041294	-2.35	0.0284	-0.0182491	-0.0011213	F(1 22)	=	5.50	0.1637
no2	-0.000413	0.0020695	-0.2	0.8445	-0.0048241	0.003998	F(1 15)	=	0.04	-0.0638
co	-0.0030116	0.0016551	-1.82	0.0855	-0.0064889	0.0004657	F(1 18)	=	3.31	0.1084
soil ph	-0.0051512	0.0047035	-1.1	0.2821	-0.0147571	0.0044546	F(1 30)	=	1.20	0.0064
soil organic carbon density	0.0123402	0.005064	2.44	0.021	0.0019981	0.0226823	F(1 30)	=	5.94	0.1374
soil organic carbon	0.0003372	0.0002477	1.36	0.1846	-0.000171	0.0008455	F(1 27)	=	1.85	0.0296
soil total carbon	-0.0000887	0.0001591	-0.56	0.5819	-0.0004151	0.0002378	F(1 27)	=	0.31	-0.0252
soil total phosphorus	-0.0002435	0.0001453	-1.68	0.1041	-0.0005402	0.0000532	F(1 30)	=	2.81	0.0552
soil moisture(0-10cm)	0.0001155	0.0119732	0.01	0.9924	-0.0244105	0.0246415	F(1 28)	=	0.00	-0.0357
soil moisture(10-40cm)	0.0015453	0.0043255	0.36	0.7236	-0.0073151	0.0104057	F(1 28)	=	0.13	-0.031
soil tmp	0.006014	0.005843	1.03	0.3122	-0.0059548	0.0179827	F(1 28)	=	1.06	0.002
precipitation	7.66E-06	0.0000557	0.14	0.8914	-0.000106	0.0001214	F(1 30)	=	0.02	-0.0327
SDP	0.0005415	0.0011202	0.48	0.6323	-0.0017463	0.0028293	F(1 30)	=	0.23	-0.0253
PCI	-0.0000475	0.0047461	-0.01	0.9921	-0.0097404	0.0096454	F(1 30)	=	0.00	-0.0333
urban-rural temperature difference day	0.0124728	0.0359664	0.35	0.7319	-0.0619293	0.0868749	F(1 23)	=	0.12	-0.0381
urban-rural temperature difference nigh	-0.049704	0.0317641	-1.56	0.1313	-0.1154132	0.0160051	F(1 23)	=	2.45	0.0569
greenness fraction	0.0492873	0.2026258	0.24	0.8095	-0.3645298	0.4631043	F(1 30)	=	0.06	-0.0313
DHI	0.1634158	0.0791845	2.06	0.0478	0.0016995	0.3251321	F(1 30)	=	4.26	0.0951
DNI	-0.0046543	0.0303948	-0.15	0.8793	-0.0667288	0.0574202	F(1 30)	=	0.02	-0.0325

GHI	0.0684028	0.0441496	1.55	0.1318	-0.0217627	0.1585683	F(1 30)	=	2.40	0.0432
UVindex	0.038758	0.0114908	3.37	0.0021	0.0152906	0.0622255	F(1 30)	=	11.38	0.2508
wet	-0.0004738	0.0083894	-0.06	0.9553	-0.0176073	0.0166596	F(1 30)	=	0.00	-0.0332
pet	0.0074753	0.0388611	0.19	0.8488	-0.0718897	0.0868402	F(1 30)	=	0.04	-0.0321
RH	0.0035716	0.0040861	0.87	0.3915	-0.0049024	0.0120457	F(1 22)	=	0.76	-0.0104
vap	0.0074701	0.0056603	1.32	0.1969	-0.0040898	0.0190299	F(1 30)	=	1.74	0.0234
temperature	0.0064697	0.0056197	1.15	0.2587	-0.0050073	0.0179467	F(1 30)	=	1.33	0.0104
fire carbon emissions	-0.0004884	0.0009051	-0.54	0.5935	-0.0023368	0.0013601	F(1 30)	=	0.29	-0.0234
GDP	-0.0037313	0.0016898	-2.21	0.0353	-0.0071874	-0.0002751	F(1 29)	=	4.88	0.1144
anti consum	-0.0108229	0.0051132	-2.12	0.0427	-0.0212654	-0.0003804	F(1 30)	=	4.48	0.1009
coastal city	-0.0547944	0.0759937	-0.72	0.4765	-0.2099942	0.1004055	F(1 30)	=	0.52	-0.0157
main clima~l	0.1375207	0.178254	0.77	0.4469	-0.2276161	0.5026576	F(3 28)	=	0.49	-0.052
main climate snow	0.0249832	0.189067	0.13	0.8958	-0.362303	0.4122693				
main climate warm	0.0189674	0.1619073	0.12	0.9076	-0.3126846	0.3506195				
soil type Andisols	-0.0622595	0.1727697	-0.36	0.7219	-0.4196609	0.295142	F(8 23)	=	0.26	-0.2353
soil type Entisols	-0.020751	0.1586991	-0.13	0.8971	-0.3490451	0.3075432				
soil type Inceptisols	0.0613602	0.1348683	0.45	0.6534	-0.2176362	0.3403566				
soil type Gelisols	-0.0068312	0.1979326	-0.03	0.9728	-0.416286	0.4026236				
soil type Oxisols	-0.059873	0.2591546	-0.23	0.8193	-0.5959751	0.4762292				
soil type Spodosols	-0.0207483	0.1979326	-0.1	0.9174	-0.4302031	0.3887065				
soil type Ultisols	-0.0054285	0.1432531	-0.04	0.9701	-0.3017701	0.2909131				
soil type Vertisols	-0.2650012	0.2591546	-1.02	0.3172	-0.8011033	0.271101				
vegetation Forests	0.028558	0.2186999	0.13	0.897	-0.4187336	0.4758496	F(2 29)	=	0.73	-0.018
vegetation Grasslands	0.1650808	0.2401079	0.69	0.4972	-0.3259951	0.6561567				
Europe	-0.0152013	0.0805688	-0.19	0.8519	-0.1811358	0.1507332	F(6 25)	=	4.26	0.3871
the Middle East	0.0234587	0.1781443	0.13	0.8963	-0.3434362	0.3903537				
North America	0.0102765	0.0975736	0.11	0.917	-0.1906802	0.2112331				
Oceania	0.0258397	0.1336082	0.19	0.8482	-0.2493315	0.3010109				
South America	0.4210433	0.1044463	4.03	0.0005	0.2059321	0.6361545				
Sub Saharan Africa	0.269295	0.1336082	2.02	0.0547	-0.0058763	0.5444662				

Aminocoumarins rpkm	Coef.	Std. Err.	t	P>t	[95% Conf.	Interval]			F	AdjR-squared
city longitude	-0.0000579	0.000179	-0.32	0.7486	-0.0004235	0.0003077	F(1 30)	=	0.10	-0.0297
city latitude	-0.0005254	0.0004989	-1.05	0.3007	-0.0015442	0.0004934	F(1 30)	=	1.11	0.0035
city total population	0.0000318	0.0004648	0.07	0.946	-0.0009204	0.0009839	F(1 28)	=	0.00	-0.0355
city population density	-2.39E-06	2.45E-06	-0.98	0.3369	-0.00000741	0.00000263	F(1 27)	=	0.96	-0.0016
city ave june temp	0.0021823	0.0018221	1.2	0.2415	-0.0015564	0.0059209	F(1 27)	=	1.43	0.0153
pm25	0.0014166	0.0007874	1.8	0.0821	-0.0001914	0.0030247	F(1 30)	=	3.24	0.0673
pm10	0.0001726	0.0005621	0.31	0.764	-0.001052	0.0013972	F(1 12)	=	0.09	-0.0749
o3	-0.0023907	0.0017991	-1.33	0.1975	-0.0061217	0.0013404	F(1 22)	=	1.77	0.0322
no2	-0.0000147	0.0005294	-0.03	0.9783	-0.0011431	0.0011138	F(1 15)	=	0.00	-0.0666
co	-0.0006034	0.0006971	-0.87	0.3981	-0.002068	0.0008612	F(1 18)	=	0.75	-0.0134
soil ph	0.0004515	0.0017597	0.26	0.7993	-0.0031423	0.0040452	F(1 30)	=	0.07	-0.0311
soil organic carbon density	-0.0018213	0.0020082	-0.91	0.3717	-0.0059226	0.00228	F(1 30)	=	0.82	-0.0058
soil organic carbon	-0.000017	0.0000957	-0.18	0.8604	-0.0002134	0.0001794	F(1 27)	=	0.03	-0.0358
soil total carbon	0.0000483	0.0000567	0.85	0.4021	-0.0000681	0.0001647	F(1 27)	=	0.72	-0.0099
soil total phosphorus	7.11E-07	0.0000558	0.01	0.9899	-0.0001132	0.0001146	F(1 30)	=	0.00	-0.0333
soil moisture(0-10cm)	-0.0109027	0.0038566	-2.83	0.0086	-0.0188027	-0.0030027	F(1 28)	=	7.99	0.1943
soil moisture(10-40cm)	-0.0032153	0.001462	-2.2	0.0363	-0.00621	-0.0002205	F(1 28)	=	4.84	0.1168
soil tmp	0.0049596	0.0019613	2.53	0.0174	0.000942	0.0089773	F(1 28)	=	6.39	0.1568
precipitation	7.60E-06	0.0000204	0.37	0.7123	-0.0000341	0.0000493	F(1 30)	=	0.14	-0.0286
SDP	0.000169	0.0004119	0.41	0.6844	-0.0006721	0.0010102	F(1 30)	=	0.17	-0.0276
PCI	-0.001254	0.001728	-0.73	0.4737	-0.0047829	0.002275	F(1 30)	=	0.53	-0.0155
urban-rural temperature difference day	0.0004907	0.0134028	0.04	0.9711	-0.027235	0.0282165	F(1 23)	=	0.00	-0.0434
urban-rural temperature difference nigh	-0.0112333	0.012196	-0.92	0.3666	-0.0364627	0.0139961	F(1 23)	=	0.85	-0.0064
greenness fraction	0.0665798	0.0734909	0.91	0.3722	-0.0835087	0.2166683	F(1 30)	=	0.82	-0.0058
DHI	0.0852985	0.0268933	3.17	0.0035	0.030375	0.1402221	F(1 30)	=	10.06	0.2262
DNI	-0.0179573	0.010675	-1.68	0.1029	-0.0397586	0.0038441	F(1 30)	=	2.83	0.0557
GHI	0.0124269	0.0166971	0.74	0.4625	-0.0216731	0.0465269	F(1 30)	=	0.55	-0.0146
UVindex	0.0072369	0.0047768	1.52	0.1402	-0.0025185	0.0169924	F(1 30)	=	2.30	0.0401
wet	-8.44E-06	0.0030812	0	0.9978	-0.0063012	0.0062843	F(1 30)	=	0.00	-0.0333
pet	0.0031052	0.0142696	0.22	0.8292	-0.0260372	0.0322477	F(1 30)	=	0.05	-0.0317
RH	0.0011746	0.0016646	0.71	0.4878	-0.0022776	0.0046269	F(1 22)	=	0.50	-0.0223
vap	0.0057344	0.0018644	3.08	0.0045	0.0019267	0.0095421	F(1 30)	=	9.46	0.2144
temperature	0.0050218	0.0018993	2.64	0.0129	0.0011429	0.0089006	F(1 30)	=	6.99	0.162
fire carbon emissions	6.47E-07	0.000334	0	0.9985	-0.0006815	0.0006828	F(1 30)	=	0.00	-0.0333
GDP	-0.0017892	0.0005805	-3.08	0.0045	-0.0029764	-0.0006019	F(1 29)	=	9.50	0.2208
anti consum	-0.0031005	0.001932	-1.6	0.119	-0.0070462	0.0008451	F(1 30)	=	2.58	0.0484
coastal city	-0.0165686	0.0279871	-0.59	0.5583	-0.073726	0.0405887	F(1 30)	=	0.35	-0.0214
main clima~l	0.1302151	0.05642	2.31	0.0286	0.0146441	0.2457861	F(3 28)	=	3.89	0.2187
main climate snow	0.0145067	0.0598424	0.24	0.8102	-0.1080749	0.1370883				
main climate warm	0.0301561	0.051246	0.59	0.5609	-0.0748165	0.1351287				
soil type Andisols	-0.0160043	0.0589278	-0.27	0.7884	-0.1379057	0.1058971	F(8 23)	=	0.76	-0.0654
soil type Entisols	-0.0775198	0.0541286	-1.43	0.1656	-0.1894934	0.0344538				
soil type Inceptisols	-0.0690648	0.0460005	-1.5	0.1469	-0.1642241	0.0260944				
soil type Gelisols	-0.0989048	0.0675103	-1.47	0.1564	-0.2385604	0.0407508				
soil type Oxisols	-0.0867069	0.0883917	-0.98	0.3368	-0.269559	0.0961452				
soil type Spodosols	-0.0973949	0.0675103	-1.44	0.1626	-0.2370505	0.0422607				
soil type Ultisols	-0.0206948	0.0488603	-0.42	0.6758	-0.1217701	0.0803805				
soil type Vertisols	-0.0948548	0.0883917	-1.07	0.2943	-0.2777069	0.0879973				
vegetation Forests	0.0277982	0.0676386	0.41	0.6841	-0.1105382	0.1661346	F(2 29)	=	6.97	0.2781
vegetation Grasslands	0.1585622	0.0742595	2.14	0.0413	0.0066844	0.3104401				
Europe	0.0023754	0.0297685	0.08	0.937	-0.058934	0.0636849	F(6 25)	=	4.16	0.3797
the Middle East	-0.0327527	0.0658207	-0.5	0.6231	-0.1683129	0.1028076				
North America	-0.0247566	0.0360515	-0.69	0.4986	-0.099006	0.0494928				
Oceania	-0.0398509	0.0493655	-0.81	0.4271	-0.1415211	0.0618193				
South America	0.0326838	0.0385908	0.85	0.4051	-0.0467954	0.1121631				
Sub Saharan Africa	0.2080442	0.0493655	4.21	0.0003	0.1063739	0.3097144				

Aminoglycosides rpkm	Coef.	Std. Err.	t	P>t	[95% Conf.	Interval]			F	AdjR-squared
city longitude	-0.0001202	0.0002243	-0.54	0.5959	-0.0005782	0.0003378	F(1 30)	=	0.29	-0.0235
city latitude	-0.0001766	0.0006376	-0.28	0.7837	-0.0014787	0.0011254	F(1 30)	=	0.08	-0.0307
city total population	-0.0001133	0.0005863	-0.19	0.8481	-0.0013144	0.0010877	F(1 28)	=	0.04	-0.0343
city population density	-4.21E-06	3.79E-06	-1.11	0.2763	-0.000012	0.00000357	F(1 27)	=	1.23	0.0083
city ave june temp	0.002324	0.0028775	0.81	0.4264	-0.0035802	0.0082281	F(1 27)	=	0.65	-0.0126
pm25	0.0007815	0.0010317	0.76	0.4547	-0.0013254	0.0028884	F(1 30)	=	0.57	-0.0139
pm10	-0.001461	0.0022743	-0.64	0.5327	-0.0064161	0.0034942	F(1 12)	=	0.41	-0.0473
o3	-0.0037351	0.0017649	-2.12	0.0459	-0.0073952	-0.000075	F(1 22)	=	4.48	0.1314
no2	-0.0019235	0.0017204	-1.12	0.2811	-0.0055904	0.0017435	F(1 15)	=	1.25	0.0154
co	-0.0004811	0.0003828	-1.26	0.2249	-0.0012854	0.0003231	F(1 18)	=	1.58	0.0296
soil ph	0.0016941	0.002192	0.77	0.4456	-0.0027825	0.0061707	F(1 30)	=	0.60	-0.0132
soil organic carbon density	0.003738	0.0024652	1.52	0.1399	-0.0012966	0.0087727	F(1 30)	=	2.30	0.0402
soil organic carbon	0.0001715	0.0001159	1.48	0.1503	-0.0000662	0.0004092	F(1 27)	=	2.19	0.0409
soil total carbon	-0.0000571	0.0000769	-0.74	0.464	-0.0002149	0.0001007	F(1 27)	=	0.55	-0.0163
soil total phosphorus	0.0000245	0.00007	0.35	0.7292	-0.0001184	0.0001673	F(1 30)	=	0.12	-0.0291
soil moisture(0-10cm)	-0.0083482	0.0052717	-1.58	0.1245	-0.0191468	0.0024504	F(1 28)	=	2.51	0.0494
soil moisture(10-40cm)	-0.0028784	0.0019168	-1.5	0.1444	-0.0068047	0.001048	F(1 28)	=	2.26	0.0415
soil tmp	0.0026376	0.0026899	0.98	0.3352	-0.0028723	0.0081476	F(1 28)	=	0.96	-0.0013
precipitation	0.0000216	0.0000254	0.85	0.4015	-0.0000303	0.0000735	F(1 30)	=	0.72	-0.009
SDP	0.0005057	0.0005107	0.99	0.33	-0.0005374	0.0015487	F(1 30)	=	0.98	-0.0006
PCI	-0.0012633	0.0021782	-0.58	0.5663	-0.0057118	0.0031852	F(1 30)	=	0.34	-0.0219
urban-rural temperature difference day	-0.0162788	0.0172874	-0.94	0.3562	-0.0520405	0.0194828	F(1 23)	=	0.89	-0.0047
urban-rural temperature difference nigh	-0.019823	0.0157917	-1.26	0.222	-0.0524906	0.0128447	F(1 23)	=	1.58	0.0234
greenness fraction	-0.005475	0.0936006	-0.06	0.9537	-0.1966329	0.1856828	F(1 30)	=	0.00	-0.0332
DHI	0.0619506	0.0373786	1.66	0.1079	-0.0143866	0.1382879	F(1 30)	=	2.75	0.0533
DNI	-0.0165348	0.0137044	-1.21	0.237	-0.044523	0.0114534	F(1 30)	=	1.46	0.0145
GHI	0.0020867	0.0211715	0.1	0.9221	-0.0411513	0.0453248	F(1 30)	=	0.01	-0.033
UVindex	0.0057444	0.0061391	0.94	0.3569	-0.0067932	0.0182821	F(1 30)	=	0.88	-0.004
wet	0.0009185	0.0038684	0.24	0.8139	-0.0069818	0.0088188	F(1 30)	=	0.06	-0.0314
pet	0.0017845	0.0179429	0.1	0.9214	-0.0348597	0.0384288	F(1 30)	=	0.01	-0.033
RH	0.0018655	0.0016971	1.1	0.2835	-0.001654	0.0053851	F(1 22)	=	1.21	0.009
vap	0.0042739	0.0025712	1.66	0.1069	-0.0009773	0.0095251	F(1 30)	=	2.76	0.0538
temperature	0.0030576	0.0025908	1.18	0.2472	-0.0022335	0.0083486	F(1 30)	=	1.39	0.0125
fire carbon emissions	0.0000121	0.0004197	0.03	0.9772	-0.0008451	0.0008693	F(1 30)	=	0.00	-0.0333
GDP	-0.0006966	0.0008344	-0.83	0.4106	-0.0024032	0.0010099	F(1 29)	=	0.70	-0.0102
anti consum	0.0005221	0.0025281	0.21	0.8378	-0.0046409	0.0056851	F(1 30)	=	0.04	-0.0319
coastal city	-0.0460513	0.0343607	-1.34	0.1902	-0.1162252	0.0241227	F(1 30)	=	1.80	0.025
main clima~l	0.0963332	0.0775103	1.24	0.2242	-0.0624395	0.2551059	F(3 28)	=	1.73	0.0661
main climate snow	0.1101987	0.0822121	1.34	0.1909	-0.0582051	0.2786026				

main climate warm	0.0226523	0.0704022	0.32	0.75	-0.1215602	0.1668647				
soil type Andisols	-0.0669421	0.0681354	-0.98	0.3361	-0.207891	0.0740067	F(8 23)	=	1.42	0.098
soil type Entisols	-0.0691982	0.0625864	-1.11	0.2803	-0.198668	0.0602716				
soil type Inceptisols	-0.0527242	0.0531882	-0.99	0.3319	-0.1627523	0.057304				
soil type Gelisols	0.1463233	0.0780589	1.87	0.0736	-0.0151539	0.3078005				
soil type Oxisols	-0.0627736	0.1022031	-0.61	0.5451	-0.2741969	0.1486497				
soil type Spodosols	-0.0673726	0.0780589	-0.86	0.397	-0.2288498	0.0941046				
soil type Ultisols	0.017209	0.0564949	0.3	0.7634	-0.0996596	0.1340776				
soil type Vertisols	-0.0718243	0.1022031	-0.7	0.4893	-0.2832476	0.139599				
vegetation Forests	0.0312098	0.0923418	0.34	0.7378	-0.1576504	0.22007	F(2 29)	=	3.69	0.1479
vegetation Grasslands	0.1607633	0.1013809	1.59	0.1236	-0.046584	0.3681105				
Europe	-0.056582	0.0467666	-1.21	0.2376	-0.1528996	0.0397355	F(6 25)	=	1.16	0.0305
the Middle East	-0.0665232	0.1034048	-0.64	0.5259	-0.2794893	0.1464429				
North America	0.0219811	0.0566371	0.39	0.7012	-0.0946652	0.1386275				
Oceania	-0.0662708	0.0775536	-0.85	0.4009	-0.2259954	0.0934537				
South America	-0.0356125	0.0606264	-0.59	0.5622	-0.1604749	0.08925				
Sub Saharan Africa	0.1054935	0.0775536	1.36	0.1859	-0.0542311	0.2652181				
Elfamycins rpkm	Coef.	Std. Err.	t	P>t	[95% Conf.	Interval]			F	AdjR-squared
city longitude	-0.0022166	0.0017628	-1.26	0.2183	-0.0058168	0.0013835	F(1 30)	=	1.58	0.0184
city latitude	-0.0051834	0.0050357	-1.03	0.3116	-0.0154678	0.0051009	F(1 30)	=	1.06	0.0019
city total population	-0.003907	0.0046376	-0.84	0.4067	-0.0134067	0.0055927	F(1 28)	=	0.71	-0.0101
city population density	-0.0000148	0.0000273	-0.54	0.5927	-0.0000708	0.0000413	F(1 27)	=	0.29	-0.0259
city ave june temp	0.0065066	0.0205908	0.32	0.7544	-0.0357422	0.0487554	F(1 27)	=	0.10	-0.0332
pm25	0.0140706	0.0079549	1.77	0.0871	-0.0021755	0.0303167	F(1 30)	=	3.13	0.0643
pm10	-0.0050079	0.0113666	-0.44	0.6673	-0.0297735	0.0197577	F(1 12)	=	0.19	-0.0661
o3	-0.0146873	0.0132232	-1.11	0.2787	-0.0421107	0.012736	F(1 22)	=	1.23	0.0101
no2	-0.0036089	0.0086722	-0.42	0.6832	-0.0220933	0.0148756	F(1 15)	=	0.17	-0.0545
co	-0.0067669	0.0054252	-1.25	0.2283	-0.0181647	0.0046309	F(1 18)	=	1.56	0.0284
soil ph	-0.0042108	0.0177517	-0.24	0.8141	-0.0404645	0.032043	F(1 30)	=	0.06	-0.0314
soil organic carbon density	0.0103389	0.0204446	0.51	0.6168	-0.0314147	0.0520924	F(1 30)	=	0.26	-0.0246
soil organic carbon	-0.0000364	0.0009651	-0.04	0.9702	-0.0020166	0.0019438	F(1 27)	=	0.00	-0.037
soil total carbon	-0.0004977	0.0005922	-0.84	0.4081	-0.0017129	0.0007175	F(1 27)	=	0.71	-0.0106
soil total phosphorus	-0.0001007	0.0005624	-0.18	0.8591	-0.0012493	0.0010479	F(1 30)	=	0.03	-0.0322
soil moisture(0-10cm)	-0.1102559	0.0389736	-2.83	0.0085	-0.1900897	-0.030422	F(1 28)	=	8.00	0.1945
soil moisture(10-40cm)	-0.0308379	0.0149031	-2.07	0.0479	-0.0613655	-0.0003102	F(1 28)	=	4.28	0.1017
soil tmp	0.0428637	0.0204231	2.1	0.045	0.0010288	0.0846985	F(1 28)	=	4.40	0.1051
precipitation	-0.0000148	0.0002063	-0.07	0.9435	-0.0004361	0.0004066	F(1 30)	=	0.01	-0.0332
SDP	0.0003589	0.0041653	0.09	0.9319	-0.0081478	0.0088656	F(1 30)	=	0.01	-0.0331
PCI	-0.0092789	0.0174995	-0.53	0.5998	-0.0450177	0.0264598	F(1 30)	=	0.28	-0.0237
urban-rural temperature difference day	0.0312415	0.1406271	0.22	0.8262	-0.2596679	0.3221508	F(1 23)	=	0.05	-0.0412
urban-rural temperature difference nigh	0.0076136	0.1304302	0.06	0.954	-0.2622018	0.277429	F(1 23)	=	0.00	-0.0433
greenness fraction	0.5582608	0.7443904	0.75	0.4591	-0.9619874	2.078509	F(1 30)	=	0.56	-0.0143
DHI	0.8306501	0.2743285	3.03	0.005	0.2703964	1.390904	F(1 30)	=	9.17	0.2085
DNI	-0.0821781	0.1116333	-0.74	0.4674	-0.3101636	0.1458075	F(1 30)	=	0.54	-0.015
GHI	0.2236978	0.1649826	1.36	0.1853	-0.1132416	0.5606372	F(1 30)	=	1.84	0.0263
UVindex	0.0793572	0.0478441	1.66	0.1076	-0.0183535	0.177068	F(1 30)	=	2.75	0.0535
wet	-0.0235588	0.0307798	-0.77	0.45	-0.0864197	0.039302	F(1 30)	=	0.59	-0.0135
pet	0.07926	0.143315	0.55	0.5843	-0.2134282	0.3719483	F(1 30)	=	0.31	-0.0229
RH	0.009562	0.0120626	0.79	0.4364	-0.0154543	0.0345784	F(1 22)	=	0.63	-0.0164
vap	0.0473412	0.0197601	2.4	0.023	0.0069856	0.0876967	F(1 30)	=	5.74	0.1326
temperature	0.0418131	0.0198552	2.11	0.0437	0.0012633	0.082363	F(1 30)	=	4.43	0.0997
fire carbon emissions	0.0001675	0.003369	0.05	0.9607	-0.0067128	0.0070478	F(1 30)	=	0.00	-0.0332
GDP	-0.0139539	0.0060068	-2.32	0.0274	-0.0262391	-0.0016686	F(1 29)	=	5.40	0.1278
anti consum	-0.0272163	0.0196888	-1.38	0.1771	-0.0674262	0.0129936	F(1 30)	=	1.91	0.0285
coastal city	-0.2482304	0.2802955	-0.89	0.3829	-0.8206703	0.3242094	F(1 30)	=	0.78	-0.007
main clima~l	1.215187	0.5701132	2.13	0.042	0.047363	2.383011	F(3 28)	=	3.84	0.2158
main climate snow	0.411521	0.6046963	0.68	0.5018	-0.8271433	1.650185				
main climate warm	0.1402784	0.517831	0.27	0.7885	-0.9204503	1.201007				
soil type Andisols	-0.9692627	0.5904993	-1.64	0.1143	-2.190804	0.2522782	F(8 23)	=	0.81	-0.0516
soil type Entisols	-1.036655	0.5424082	-1.91	0.0685	-2.158712	0.0854015				
soil type Inceptisols	-0.792274	0.4609584	-1.72	0.0991	-1.745839	0.1612911				
soil type Gelisols	-0.2775923	0.6765019	-0.41	0.6854	-1.677043	1.121859				
soil type Oxisols	-1.118429	0.885749	-1.26	0.2194	-2.95074	0.7138826				
soil type Spodosols	-1.115489	0.6765019	-1.65	0.1128	-2.51494	0.2839623				
soil type Ultisols	-0.7131218	0.4896162	-1.46	0.1588	-1.72597	0.2997264				
soil type Vertisols	-1.113876	0.885749	-1.26	0.2212	-2.946188	0.718435				
vegetation Forests	0.1955049	0.7018299	0.28	0.7826	-1.239899	1.630908	F(2 29)	=	5.79	0.236
vegetation Grasslands	1.437752	0.7705304	1.87	0.0722	-0.1381593	3.013664				
Europe	0.0983937	0.2435219	0.4	0.6896	-0.4031489	0.5999364	F(6 25)	=	8.50	0.592
the Middle East	-0.0390184	0.5384471	-0.07	0.9428	-1.147971	1.069934				
North America	0.2990766	0.2949196	1.01	0.3203	-0.3083217	0.906475				
Oceania	-0.0872135	0.4038353	-0.22	0.8308	-0.918928	0.7445009				
South America	0.5183755	0.3156926	1.64	0.1131	-0.1318056	1.168557				
Sub Saharan Africa	2.671641	0.4038353	6.62	0	1.839926	3.503355				
MLS rpkm	Coef.	Std. Err.	t	P>t	[95% Conf.	Interval]			F	AdjR-squared
city longitude	-0.001856	0.001161	-1.6	0.1204	-0.0042272	0.0005151	F(1 30)	=	2.56	0.0478
city latitude	0.0017743	0.003411	0.52	0.6068	-0.005192	0.0087406	F(1 30)	=	0.27	-0.0241
city total population	-0.0021698	0.0031361	-0.69	0.4947	-0.0085937	0.0042541	F(1 28)	=	0.48	-0.0183
city population density	-0.0000161	0.000021	-0.77	0.4499	-0.0000591	0.000027	F(1 27)	=	0.59	-0.0149
city ave june temp	-0.0010819	0.0159341	-0.07	0.9464	-0.0337761	0.0316122	F(1 27)	=	0.00	-0.0369
pm25	-0.0042898	0.0055349	-0.78	0.4444	-0.0155935	0.0070139	F(1 30)	=	0.60	-0.013
pm10	-0.0094433	0.0156966	-0.6	0.5586	-0.0436433	0.0247567	F(1 12)	=	0.36	-0.0516
o3	-0.0045167	0.0029498	-1.53	0.14	-0.0106342	0.0016007	F(1 22)	=	2.34	0.0552
no2	-0.0130888	0.0115802	-1.13	0.2761	-0.0377714	0.0115939	F(1 15)	=	1.28	0.017
co	-0.0016456	0.0011739	-1.4	0.178	-0.0041118	0.0008207	F(1 18)	=	1.97	0.0483
soil ph	0.0122721	0.0116686	1.05	0.3013	-0.0115584	0.0361026	F(1 30)	=	1.11	0.0034
soil organic carbon density	0.034013	0.012245	2.78	0.0093	0.0090055	0.0590206	F(1 30)	=	7.72	0.1781
soil organic carbon	0.0011082	0.0006156	1.8	0.083	-0.000155	0.0023714	F(1 27)	=	3.24	0.0741
soil total carbon	-0.0000743	0.0004215	-0.18	0.8614	-0.0009392	0.0007906	F(1 27)	=	0.03	-0.0358
soil total phosphorus	0.0002016	0.0003745	0.54	0.5942	-0.0005632	0.0009665	F(1 30)	=	0.29	-0.0234
soil moisture(0-10cm)	0.0120628	0.0296202	0.41	0.6869	-0.0486114	0.072737	F(1 28)	=	0.17	-0.0296
soil moisture(10-40cm)	0.0001744	0.0107568	0.02	0.9872	-0.0218599	0.0222086	F(1 28)	=	0.00	-0.0357
soil tmp	-0.013626	0.014543	-0.94	0.3568	-0.0434159	0.016164	F(1 28)	=	0.88	-0.0042
precipitation	-0.0000417	0.0001378	-0.3	0.7641	-0.0003231	0.0002396	F(1 30)	=	0.09	-0.0302
SDP	-0.0010463	0.0027791	-0.38	0.7092	-0.006722	0.0046295	F(1 30)	=	0.14	-0.0285
PCI	-0.0047084	0.0117252	-0.4	0.6909	-0.0286546	0.0192377	F(1 30)	=	0.16	-0.0278
urban-rural temperature difference day	-0.0187137	0.096288	-0.19	0.8476	-0.2179006	0.1804732	F(1 23)	=	0.04	-0.0418
urban-rural temperature difference nigh	0.0446501	0.0888036	0.5	0.6199	-0.1390541	0.2283542	F(1 23)	=	0.25	-0.0321
greenness fraction	-0.03491	0.5023823	-0.07	0.9451	-1.060911	0.9910916	F(1 30)	=	0.00	-0.0332
DHI	-0.1519672	0.2077663	-0.73	0.4702	-0.5762827	0.2723483	F(1 30)	=	0.53	-0.0152
DNI	0.0279885	0.0751475	0.37	0.7122	-0.1254831	0.1814602	F(1 30)	=	0.14	-0.0286
GHI	-0.0396423	0.1134243	-0.35	0.7292	-0.2712856	0.192001	F(1 30)	=	0.12	-0.0291
UVindex	-0.0173218	0.0332785	-0.52	0.6065	-0.0852857	0.050642	F(1 30)	=	0.27	-0.0241
wet	-0.0010021	0.0207819	-0.05	0.9619	-0.0434443	0.0414401	F(1 30)	=	0.00	-0.0333
pet	-0.0486844	0.0959119	-0.51	0.6154	-0.2445626	0.1471938	F(1 30)	=	0.26	-0.0245
RH	0.0017232	0.0027693	0.62	0.5402	-0.0040199	0.0074663	F(1 22)	=	0.39	-0.0274
vap	-0.0088916	0.0143308	-0.62	0.5396	-0.038159	0.0203759	F(1 30)	=	0.38	-0.0202
temperature	-0.0134914	0.01401	-0.96	0.3433	-0.0421036	0.0151208	F(1 30)	=	0.93	-0.0023
fire carbon emissions	-0.000084	0.0022529	-0.04	0.9705	-0.004685	0.004517	F(1 30)	=	0.00	-0.0333
GDP	0.0034469	0.0044842	0.77	0.4483	-0.0057244	0.0126182	F(1 29)	=	0.59	-0.0138
anti consum	0.0123363	0.0133908	0.92	0.3643	-0.0150113	0.0396838	F(1 30)	=	0.85	-0.0049
coastal city	-0.1607788	0.1875867	-0.86	0.3982	-0.5438819	0.2223244	F(1 30)	=	0.73	-0.0086
main clima~l	0.0882551	0.406499	0.22	0.8297	-0.7444203	0.9209305	F(3 28)	=	2.26	0.1084
main climate snow	0.7377054	0.4311573	1.71	0.0981	-0.1454802	1.620891				
main climate warm	0.041299	0.369221	0.11	0.9117	-0.715016	0.797614				
soil type Andisols	0.0398916	0.3283175	0.12	0.9043	-0.6392849	0.7190681	F(8 23)	=	2.46	0.273
soil type Entisols	0.0055676	0.3015789	0.02	0.9854	-0.6182959	0.6294311				
soil type Inceptisols	0.0309114	0.2562928	0.12	0.905	-0.4992706	0.5610934				
soil type Gelisols	1.475776	0.376135	3.92	0.0007	0.6976817	2.253871				
soil type Oxisols	-0.0036339	0.4924763	-0.01	0.9942	-1.022399	1.015131				
soil type Spodosols	-0.0291185	0.376135	-0.08	0.939	-0.8072129	0.748976				
soil type Ultisols	0.0915499	0.2722265	0.34	0.7397	-0.4715935	0.6546933				
soil type Vertisols	-0.0276844	0.4924763	-0.06	0.9557	-1.046449	0.9910804				
vegetation Forests	0.1544097	0.553643	0.28	0.7823	-0.9779175	1.286737	F(2 29)	=	0.08	-0.0632
vegetation Grasslands	0.0666298	0.6078378	0.11	0.9135	-1.176538	1.309798				
Europe	-0.0007949	0.2599794	0	0.9976	-0.5362325	0.5346427	F(6 25)	=	0.80	-0.04
the Middle East	-0.0397903	0.5748361	-0.07	0.9454	-1.223687	1.144107				

North America	0.5731575	0.3148507	1.82	0.0807	-0.0752896	1.221605				
Oceania	-0.0491203	0.4311271	-0.11	0.9102	-0.9370431	0.8388025				
South America	0.1155109	0.3370275	0.34	0.7347	-0.5786103	0.8096321				
Sub Saharan Africa	-0.0093836	0.4311271	-0.02	0.9828	-0.8973063	0.8785392				

Multi-drug resistance rpkm	Coef.	Std. Err.	t	P>t	[95% Conf.	Interval]			F	AdjR-squared
city longitude	-0.0002377	0.0004492	-0.53	0.6007	-0.0011551	0.0006798	F(1 30)	=	0.28	-0.0238
city latitude	-0.0015428	0.0012472	-1.24	0.2257	-0.0040899	0.0010043	F(1 30)	=	1.53	0.0168
city total population	-0.0000814	0.001167	-0.07	0.9449	-0.0024719	0.0023091	F(1 28)	=	0.00	-0.0355
city population density	-9.38E-06	6.44E-06	-1.46	0.1569	-0.0000226	0.00000384	F(1 27)	=	2.12	0.0385
city ave june temp	0.0029324	0.004995	0.59	0.562	-0.0073166	0.0131813	F(1 27)	=	0.34	-0.024
pm25	0.002634	0.0020298	1.3	0.2043	-0.0015114	0.0067794	F(1 30)	=	1.68	0.0216
pm10	-0.0012902	0.0016305	-0.79	0.4441	-0.0048428	0.0022624	F(1 12)	=	0.63	-0.0296
o3	-0.0123527	0.0039273	-3.15	0.0047	-0.0204975	-0.0042079	F(1 22)	=	9.89	0.2788
no2	-0.0011642	0.002346	-0.5	0.6269	-0.0061646	0.0038362	F(1 15)	=	0.25	-0.0494
co	-0.0028436	0.0018068	-1.57	0.1329	-0.0066395	0.0009523	F(1 18)	=	2.48	0.0721
soil ph	-0.0023904	0.0044123	-0.54	0.592	-0.0114015	0.0066207	F(1 30)	=	0.29	-0.0233
soil organic carbon density	0.0004449	0.0051227	0.09	0.9314	-0.0100171	0.0109069	F(1 30)	=	0.01	-0.0331
soil organic carbon	0.0002324	0.0002362	0.98	0.3339	-0.0002522	0.0007169	F(1 27)	=	0.97	-0.0011
soil total carbon	-0.0001231	0.0001248	-0.99	0.3328	-0.0003792	0.000133	F(1 27)	=	0.97	-0.001
soil total phosphorus	-0.0001089	0.000139	-0.78	0.4394	-0.0003928	0.000175	F(1 30)	=	0.61	-0.0126
soil moisture(0-10cm)	-0.0229967	0.0100822	-2.28	0.0304	-0.0436493	-0.0023442	F(1 28)	=	5.20	0.1266
soil moisture(10-40cm)	-0.0066128	0.0037738	-1.75	0.0907	-0.0143432	0.0011175	F(1 28)	=	3.07	0.0666
soil tmp	0.012004	0.0049644	2.42	0.0224	0.0018348	0.0221732	F(1 28)	=	5.85	0.1432
precipitation	0.0000441	0.0000508	0.87	0.3922	-0.0000597	0.000148	F(1 30)	=	0.75	-0.008
SDP	0.0004554	0.0010362	0.44	0.6635	-0.0016608	0.0025716	F(1 30)	=	0.19	-0.0267
PCI	-0.003713	0.0043345	-0.86	0.3984	-0.0125652	0.0051391	F(1 30)	=	0.73	-0.0087
urban-rural temperature difference day	0.0099664	0.0349942	0.28	0.7783	-0.0624247	0.0823575	F(1 23)	=	0.08	-0.0398
urban-rural temperature difference nigh	0.0024824	0.0324774	0.08	0.9397	-0.0647023	0.069667	F(1 23)	=	0.01	-0.0432
greenness fraction	-0.0204359	0.1874479	-0.11	0.9139	-0.4032556	0.3623838	F(1 30)	=	0.01	-0.0329
DHI	0.2205964	0.0670528	3.29	0.0026	0.0836563	0.3575365	F(1 30)	=	10.82	0.2406
DNI	-0.0472639	0.0267495	-1.77	0.0874	-0.1018937	0.007366	F(1 30)	=	3.12	0.0641
GHI	0.0295462	0.0420673	0.7	0.4879	-0.0563666	0.115459	F(1 30)	=	0.49	-0.0166
UVindex	0.0219333	0.011814	1.86	0.0732	-0.0021942	0.0460607	F(1 30)	=	3.45	0.0732
wet	0.0048882	0.0077038	0.63	0.5306	-0.010845	0.0206214	F(1 30)	=	0.40	-0.0196
pet	0.0119757	0.0358775	0.33	0.7409	-0.0612959	0.0852473	F(1 30)	=	0.11	-0.0295
RH	0.0050071	0.004121	1.22	0.2372	-0.0035393	0.0135536	F(1 22)	=	1.48	0.0203
vap	0.0161692	0.0045	3.59	0.0012	0.0069789	0.0253595	F(1 30)	=	12.91	0.2776
temperature	0.0125104	0.0047916	2.61	0.014	0.0027246	0.0222962	F(1 30)	=	6.82	0.158
fire carbon emissions	-0.0000476	0.0008407	-0.06	0.9552	-0.0017644	0.0016693	F(1 30)	=	0.00	-0.0332
GDP	-0.0022074	0.0016314	-1.35	0.1865	-0.0055439	0.0011291	F(1 29)	=	1.83	0.0269
anti consum	-0.0097622	0.0047433	-2.06	0.0484	-0.0194493	-0.0000751	F(1 30)	=	4.24	0.0945
coastal city	-0.0420943	0.070434	-0.6	0.5546	-0.1859398	0.1017511	F(1 30)	=	0.36	-0.0212
main clima~l	0.3206892	0.12169	2.64	0.0135	0.0714186	0.5699598	F(3 28)	=	8.68	0.4262
main climate snow	0.0439629	0.1290717	0.34	0.7359	-0.2204285	0.3083543				
main climate warm	-0.0318369	0.1105304	-0.29	0.7754	-0.2582482	0.1945744				
soil type Andisols	-0.2106242	0.1453926	-1.45	0.1609	-0.5113917	0.0901433	F(8 23)	=	0.91	-0.0238
soil type Entisols	-0.2280561	0.1335516	-1.71	0.1012	-0.5043287	0.0482165				
soil type Inceptisols	-0.1892938	0.1134971	-1.67	0.1089	-0.4240803	0.0454928				
soil type Gelisols	-0.0493257	0.1665681	-0.3	0.7698	-0.3938982	0.2952467				
soil type Oxisols	-0.2519452	0.2180889	-1.16	0.2599	-0.7030965	0.199206				
soil type Spodosols	-0.1803612	0.1665681	-1.08	0.2901	-0.5249337	0.1642112				
soil type Ultisols	-0.0219906	0.1205532	-0.18	0.8569	-0.2713738	0.2273927				
soil type Vertisols	-0.2522153	0.2180889	-1.16	0.2594	-0.7033665	0.1989359				
vegetation Forests	0.0749745	0.171375	0.44	0.665	-0.2755267	0.4254757	F(2 29)	=	6.69	0.2684
vegetation Grasslands	0.3989175	0.1881505	2.12	0.0427	0.0141066	0.7837284				
Europe	-0.0642721	0.0750361	-0.86	0.3998	-0.2188119	0.0902677	F(6 25)	=	4.14	0.3778
the Middle East	-0.1132764	0.1659111	-0.68	0.501	-0.4549768	0.228424				
North America	-0.0172779	0.0908733	-0.19	0.8507	-0.204435	0.1698791				
Oceania	-0.1225571	0.1244334	-0.98	0.3341	-0.3788324	0.1337182				
South America	0.0359857	0.097274	0.37	0.7145	-0.164354	0.2363253				
Sub Saharan Africa	0.5014836	0.1244334	4.03	0.0005	0.2452083	0.757759				

beta-lactams rpkm	Coef.	Std. Err.	t	P>t	[95% Conf.	Interval]			F	AdjR-squared
city longitude	-0.0000626	0.0000338	-1.85	0.0744	-0.0001317	0.00000656	F(1 30)	=	3.42	0.0723
city latitude	-0.0000985	0.0000996	-0.99	0.3305	-0.0003019	0.0001049	F(1 30)	=	0.98	-0.0007
city total population	0.0000134	0.000093	0.14	0.8867	-0.0001771	0.0002039	F(1 28)	=	0.02	-0.0349
city population density	-8.70E-07	5.96E-07	-1.46	0.1557	-0.00000209	0.000000352	F(1 27)	=	2.13	0.0389
city ave june temp	-0.0000194	0.0004651	-0.04	0.967	-0.0009737	0.0009348	F(1 27)	=	0.00	-0.037
pm25	0.0000404	0.0001649	0.25	0.8081	-0.0002964	0.0003773	F(1 30)	=	0.06	-0.0313
pm10	-0.0001055	0.0003274	-0.32	0.7528	-0.0008188	0.0006078	F(1 12)	=	0.10	-0.074
o3	-0.0007684	0.0003392	-2.27	0.0337	-0.0014718	-0.0000649	F(1 22)	=	5.13	0.1523
no2	-0.0002792	0.0002393	-1.17	0.2616	-0.0007893	0.0002309	F(1 15)	=	1.36	0.0221
co	-0.0002436	0.0001453	-1.68	0.1109	-0.0005488	0.0000616	F(1 18)	=	2.81	0.0871
soil ph	0.0000694	0.0003507	0.2	0.8445	-0.0006468	0.0007856	F(1 30)	=	0.04	-0.032
soil organic carbon density	0.0008853	0.0003719	2.38	0.0238	0.0001258	0.0016448	F(1 30)	=	5.67	0.1308
soil organic carbon	0.0000185	0.0000187	0.99	0.3305	-0.0000198	0.0000568	F(1 27)	=	0.98	-0.0006
soil total carbon	-9.43E-06	9.88E-06	-0.95	0.3485	-0.0000297	0.0000108	F(1 27)	=	0.91	-0.0032
soil total phosphorus	-8.34E-06	0.000011	-0.76	0.4546	-0.0000308	0.0000141	F(1 30)	=	0.57	-0.0139
soil moisture(0-10cm)	-0.0000687	0.0008716	-0.08	0.9377	-0.001854	0.0017166	F(1 28)	=	0.01	-0.0355
soil moisture(10-40cm)	5.11E-07	0.0003156	0	0.9987	-0.000646	0.000647	F(1 28)	=	0.00	-0.0357
soil tmp	0.0002468	0.0004308	0.57	0.5713	-0.0006357	0.0011293	F(1 28)	=	0.33	-0.0237
precipitation	-5.09E-07	4.07E-06	-0.12	0.9014	-0.00000883	0.00000781	F(1 30)	=	0.02	-0.0328
SDP	-0.0000212	0.0000822	-0.26	0.7978	-0.0001891	0.0001466	F(1 30)	=	0.07	-0.031
PCI	-0.0002209	0.0003449	-0.64	0.5267	-0.0009252	0.0004834	F(1 30)	=	0.41	-0.0194
urban-rural temperature difference day	0.0020123	0.0027033	0.74	0.4642	-0.0035799	0.0076044	F(1 23)	=	0.55	-0.0189
urban-rural temperature difference nigh	0.0008134	0.0025291	0.32	0.7506	-0.0044183	0.0060452	F(1 23)	=	0.10	-0.0388
greenness fraction	0.0061391	0.0147963	0.41	0.6812	-0.024079	0.0363571	F(1 30)	=	0.17	-0.0274
DHI	0.0054088	0.0061114	0.89	0.3832	-0.0070724	0.01789	F(1 30)	=	0.78	-0.007
DNI	-0.0005056	0.0022226	-0.23	0.8216	-0.0050448	0.0040336	F(1 30)	=	0.05	-0.0316
GHI	0.0018154	0.0033403	0.54	0.5908	-0.0050065	0.0086372	F(1 30)	=	0.30	-0.0233
UVindex	0.0009494	0.000972	0.98	0.3365	-0.0010356	0.0029344	F(1 30)	=	0.95	-0.0015
wet	0.0000941	0.0006136	0.15	0.8792	-0.001159	0.0013471	F(1 30)	=	0.02	-0.0325
pet	-0.0006467	0.0028424	-0.23	0.8216	-0.0064516	0.0051582	F(1 30)	=	0.05	-0.0316
RH	0.0001505	0.0003376	0.45	0.6601	-0.0005497	0.0008506	F(1 22)	=	0.20	-0.0361
vap	0.0004609	0.0004176	1.1	0.2785	-0.0003919	0.0013137	F(1 30)	=	1.22	0.007
temperature	0.0002529	0.0004176	0.61	0.5493	-0.0005999	0.0011057	F(1 30)	=	0.37	-0.0209
fire carbon emissions	-4.53E-06	0.0000665	-0.07	0.9462	-0.0001404	0.0001314	F(1 30)	=	0.00	-0.0332
GDP	-0.0001862	0.0001287	-1.45	0.1588	-0.0004495	0.0000771	F(1 29)	=	2.09	0.0351
anti consum	-0.0003303	0.0003965	-0.83	0.4114	-0.00114	0.0004794	F(1 30)	=	0.69	-0.01
coastal city	-0.0054957	0.0055172	-1	0.3272	-0.0167632	0.0057719	F(1 30)	=	0.99	-0.0003
main clima~l	0.0167463	0.0113341	1.48	0.1507	-0.0064706	0.0399632	F(3 28)	=	3.67	0.2054
main climate snow	0.0182066	0.0120217	1.51	0.1411	-0.0064186	0.0428318				
main climate warm	-0.0002386	0.0102947	-0.02	0.9817	-0.0213264	0.0208492				
soil type Andisols	-0.0061133	0.0113918	-0.54	0.5967	-0.0296791	0.0174524	F(8 23)	=	0.99	-0.0034
soil type Entisols	-0.0009119	0.010464	-0.09	0.9313	-0.0225584	0.0207347				
soil type Inceptisols	-0.0058692	0.0088927	-0.66	0.5158	-0.0242652	0.0125268				
soil type Gelisols	0.0240787	0.0130509	1.84	0.078	-0.0029193	0.0510766				
soil type Oxisols	-0.0051669	0.0170877	-0.3	0.7651	-0.0405155	0.0301817				
soil type Spodosols	-0.0091276	0.0130509	-0.7	0.4913	-0.0361255	0.0178704				
soil type Ultisols	0.0041472	0.0094456	0.44	0.6647	-0.0153924	0.0236869				
soil type Vertisols	-0.0100451	0.0170877	-0.59	0.5624	-0.0453937	0.0253035				
vegetation Forests	0.0062448	0.0160079	0.39	0.6993	-0.026495	0.0389846	F(2 29)	=	0.71	-0.019
vegetation Grasslands	0.0154935	0.0175749	0.88	0.3853	-0.0204511	0.0514381				
Europe	-0.000656	0.0071847	-0.09	0.928	-0.0154533	0.0141412	F(6 25)	=	1.51	0.0894
the Middle East	-0.0025627	0.0158861	-0.16	0.8731	-0.0352807	0.0301553				
North America	0.0112109	0.0087012	1.29	0.2094	-0.0067095	0.0291312				
Oceania	-0.003752	0.0119146	-0.31	0.7554	-0.0282905	0.0207865				
South America	0.0174178	0.009314	1.87	0.0732	-0.0017648	0.0366004				
Sub Saharan Africa	0.0200681	0.0119146	1.68	0.1046	-0.0044704	0.0446066				

total abundance of AMR class	Coef.	Std. Err.	t	P>t	[95% Conf.	Interval]			F	AdjR-squared
city longitude	-0.0075881	0.00584	-1.3	0.2037	-0.019515	0.0043389	F(1 30)	=	1.69	0.0217
city latitude	-0.0117457	0.0168678	-0.7	0.4916	-0.0461943	0.0227029	F(1 30)	=	0.48	-0.0169
city total population	-0.0086777	0.0154885	-0.56	0.5798	-0.0404043	0.023049	F(1 28)	=	0.31	-0.0242
city population density	-0.0001034	0.0000948	-1.09	0.2851	-0.0002979	0.0000911	F(1 27)	=	1.19	0.0067
city ave june temp	0.0355916	0.0724576	0.49	0.6273	-0.1130793	0.1842624	F(1 27)	=	0.24	-0.0279
pm25	0.0264826	0.0273161	0.97	0.3401	-0.0293043	0.0822694	F(1 30)	=	0.94	-0.0019
pm10	-0.0336524	0.0569574	-0.59	0.5656	-0.1577519	0.0904472	F(1 12)	=	0.35	-0.0527
o3	-0.0975848	0.0383484	-2.54	0.0185	-0.1771144	-0.0180552	F(1 22)	=	6.48	0.1923

no2	-0.0459912	0.0428867	-1.07	0.3005	-0.137402	0.0454196	F(1 15)	=	1.15	0.0093
co	-0.0258214	0.0155232	-1.66	0.1135	-0.0584343	0.0067916	F(1 18)	=	2.77	0.0851
soil ph	0.0176679	0.0588756	0.3	0.7662	-0.102572	0.1379078	F(1 30)	=	0.09	-0.0302
soil organic carbon density	0.1001246	0.0656357	1.53	0.1376	-0.0339214	0.2341705	F(1 30)	=	2.33	0.0411
soil organic carbon	0.0037859	0.003115	1.22	0.2347	-0.0026055	0.0101773	F(1 27)	=	1.48	0.0168
soil total carbon	-0.0014238	0.0019576	-0.73	0.4733	-0.0054403	0.0025928	F(1 27)	=	0.53	-0.0171
soil total phosphorus	-0.0001142	0.0018673	-0.06	0.9516	-0.0039276	0.0036992	F(1 30)	=	0.00	-0.0332
soil moisture(0-10cm)	-0.2462577	0.1389963	-1.77	0.0873	-0.5309788	0.0384633	F(1 28)	=	3.14	0.0687
soil moisture(10-40cm)	-0.0775886	0.0510093	-1.52	0.1395	-0.1820765	0.0268992	F(1 28)	=	2.31	0.0433
soil tmp	0.0967586	0.0705407	1.37	0.1811	-0.0477375	0.2412547	F(1 28)	=	1.88	0.0295
precipitation	0.0002	6837	0.29	0.7719	-0.0011963	0.0015962	F(1 30)	=	0.09	-0.0304
SDP	0.0034331	0.01381	0.25	0.8054	-0.0247706	0.0316368	F(1 30)	=	0.06	-0.0312
PCI	-0.0386582	0.0579148	-0.67	0.5096	-0.1569359	0.0796195	F(1 30)	=	0.45	-0.0182
urban-rural temperature difference day	-0.0776276	0.4677925	-0.17	0.8697	-1.04533	0.8900749	F(1 23)	=	0.03	-0.0422
urban-rural temperature difference nigh	-0.0482972	0.4335824	-0.11	0.9123	-0.9452308	0.8486363	F(1 23)	=	0.01	-0.0429
greenness fraction	0.4789858	2.491761	0.19	0.8489	-4.609869	5.567841	F(1 30)	=	0.04	-0.0321
DHI	2.045616	0.9708431	2.11	0.0436	0.0628899	4.028342	F(1 30)	=	4.44	0.0999
DNI	-0.3274127	0.3689731	-0.89	0.3819	-1.080956	0.4261308	F(1 30)	=	0.79	-0.0069
GHI	0.3779607	0.5597807	0.68	0.5047	-0.7652639	1.521185	F(1 30)	=	0.46	-0.0179
UVindex	0.2008701	0.1617857	1.24	0.224	-0.1295404	0.5312806	F(1 30)	=	1.54	0.0172
wet	-0.0079838	0.1031246	-0.08	0.9388	-0.2185923	0.2026247	F(1 30)	=	0.01	-0.0331
pet	0.0851104	0.4777539	0.18	0.8598	-0.8905933	1.060814	F(1 30)	=	0.03	-0.0322
RH	0.0418301	0.0382514	1.09	0.286	-0.0374986	0.1211587	F(1 22)	=	1.20	0.0084
vap	0.1360833	0.0671215	2.03	0.0516	-0.0009971	0.2731638	F(1 30)	=	4.11	0.0912
temperature	0.1006469	0.068158	1.48	0.1502	-0.0385503	0.2398442	F(1 30)	=	2.18	0.0367
fire carbon emissions	3.55E-06	0.0111803	0	0.9997	-0.0228297	0.0228368	F(1 30)	=	0.00	-0.0333
GDP	-0.0265774	0.021917	-1.21	0.2351	-0.0714026	0.0182479	F(1 29)	=	1.47	0.0154
anti consum	-0.0460191	0.0668598	-0.69	0.4966	-0.1825649	0.0905268	F(1 30)	=	0.47	-0.0173
coastal city	-1.042357	0.9228172	-1.13	0.2676	-2.927001	0.842287	F(1 30)	=	1.28	0.0088
main clima~l	3.614311	1.903531	1.9	0.0679	-0.2848942	7.513517	F(3 28)	=	3.68	0.2061
main climate snow	2.651843	2.018999	1.31	0.1997	-1.483889	6.787575				
main climate warm	0.3686446	1.728967	0.21	0.8327	-3.172985	3.910274				
soil type Andisols	-2.240896	1.850589	-1.21	0.2382	-6.069131	1.587338	F(8 23)	=	1.26	0.0621
soil type Entisols	-2.519552	1.699874	-1.48	0.1519	-6.03601	0.9969063				
soil type Inceptisols	-1.965763	1.444615	-1.36	0.1868	-4.954178	1.022652				
soil type Gelisols	2.782527	2.120116	1.31	0.2023	-1.603266	7.168321				
soil type Oxisols	-2.580475	2.775883	-0.93	0.3622	-8.322827	3.161877				
soil type Spodosols	-2.716237	2.120116	-1.28	0.2129	-7.102031	1.669557				
soil type Ultisols	-0.6257448	1.534427	-0.41	0.6872	-3.799949	2.54846				
soil type Vertisols	-2.796249	2.775883	-1.01	0.3243	-8.538601	2.946103				
vegetation Forests	0.8699278	2.482323	0.35	0.7285	-4.206994	5.946849	F(2 29)	=	3.36	0.1321
vegetation Grasslands	4.185414	2.725312	1.54	0.1354	-1.388475	9.759303				
Europe	-0.4615932	1.115731	-0.41	0.6826	-2.759485	1.836299	F(6 25)	=	2.48	0.2222
the Middle East	-0.8187459	2.466975	-0.33	0.7427	-5.899576	4.262084				
North America	1.358042	1.351218	1.01	0.3245	-1.424844	4.140927				
Oceania	-1.055388	1.850231	-0.57	0.5735	-4.866011	2.755234				
South America	0.9067007	1.446392	0.63	0.5364	-2.0722	3.885601				
Sub Saharan Africa	5.809938	1.850231	3.14	0.0043	1.999315	9.620561				
**total prevalence of AMR class**	**Coef.**	**Std. Err.**	**t**	**P>t**	**[95% Conf.**	**Interval]**			**F**	**AdjR-squared**
city longitude	-0.0092048	0.0055718	-1.65	0.109	-0.020584	0.0021744	F(1 30)	=	2.73	0.0528
city latitude	-0.0269681	0.0157347	-1.71	0.0969	-0.0591026	0.0051664	F(1 30)	=	2.94	0.0588
city total population	0.0243278	0.0142861	1.7	0.0997	-0.0049359	0.0535915	F(1 28)	=	2.90	0.0615
city population density	-0.0000707	0.0000977	-0.72	0.4754	-0.0002713	0.0001298	F(1 27)	=	0.52	-0.0173
city ave june temp	-0.0622245	0.0731641	-0.85	0.4025	-0.2123448	0.0878959	F(1 27)	=	0.72	-0.01
pm25	0.0457675	0.0255671	1.79	0.0835	-0.0064475	0.0979826	F(1 30)	=	3.20	0.0664
pm10	0.0507927	0.0233145	2.18	0.05	-0.0000052	0.1015906	F(1 12)	=	4.75	0.2237
o3	-0.0752911	0.0468326	-1.61	0.1222	-0.172416	0.0218337	F(1 22)	=	2.58	0.0645
no2	0.0014694	0.0162363	0.09	0.9291	-0.0331375	0.0360763	F(1 15)	=	0.01	-0.0661
co	-0.0185597	0.0176631	-1.05	0.3073	-0.0556684	0.018549	F(1 18)	=	1.10	0.0054
soil ph	-0.0503373	0.0564292	-0.89	0.3795	-0.165581	0.0649065	F(1 30)	=	0.80	-0.0066
soil organic carbon density	0.1561776	0.0595937	2.62	0.0136	0.034471	0.2778841	F(1 30)	=	6.87	0.1592
soil organic carbon	0.0035156	0.0030227	1.16	0.255	-0.0026864	0.0097176	F(1 27)	=	1.35	0.0124
soil total carbon	0.0003979	0.0018969	0.21	0.8354	-0.0034941	0.0042899	F(1 27)	=	0.04	-0.0353
soil total phosphorus	-0.0006906	0.0018062	-0.38	0.7049	-0.0043795	0.0029982	F(1 30)	=	0.15	-0.0283
soil moisture(0-10cm)	-0.1113445	0.1404954	-0.79	0.4347	-0.3991362	0.1764473	F(1 28)	=	0.63	-0.013
soil moisture(10-40cm)	-0.0238564	0.0512411	-0.47	0.6451	-0.1288191	0.0811062	F(1 28)	=	0.22	-0.0278
soil tmp	0.0780239	0.0690698	1.13	0.2682	-0.0634591	0.2195069	F(1 28)	=	1.28	0.0094
precipitation	0.0000636	0.0006638	0.1	0.9242	-0.0012919	0.0014192	F(1 30)	=	0.01	-0.033
SDP	0.0040482	0.0133839	0.3	0.7644	-0.0232853	0.0313816	F(1 30)	=	0.09	-0.0302
PCI	-0.0263739	0.0563656	-0.47	0.6432	-0.1414878	0.0887401	F(1 30)	=	0.22	-0.0258
urban-rural temperature difference day	0.0981811	0.4562428	0.22	0.8315	-0.8456291	1.041991	F(1 23)	=	0.05	-0.0414
urban-rural temperature difference nigh	-0.8322231	0.3859466	-2.16	0.0418	-1.630614	-0.0338318	F(1 23)	=	4.65	0.132
greenness fraction	2.749663	2.364857	1.16	0.2541	-2.08002	7.579346	F(1 30)	=	1.35	0.0112
DHI	2.372759	0.9108322	2.61	0.0142	0.5125919	4.232927	F(1 30)	=	6.79	0.1573
DNI	-0.2864323	0.3586366	-0.8	0.4308	-1.018866	0.4460014	F(1 30)	=	0.64	-0.0118
GHI	0.648857	0.5338996	1.22	0.2337	-0.4415114	1.739225	F(1 30)	=	1.48	0.0152
UVindex	0.4376128	0.1396049	3.13	0.0038	0.1525016	0.722724	F(1 30)	=	9.83	0.2216
wet	-0.0006191	0.1000018	-0.01	0.9951	-0.2048501	0.2036119	F(1 30)	=	0.00	-0.0333
pet	-0.1338014	0.4628417	-0.29	0.7745	-1.07905	0.8114474	F(1 30)	=	0.08	-0.0305
RH	0.0351019	0.0439369	0.8	0.4329	-0.0560175	0.1262214	F(1 22)	=	0.64	-0.016
vap	0.0986484	0.0670202	1.47	0.1515	-0.0382251	0.2355219	F(1 30)	=	2.17	0.0363
temperature	0.0821586	0.0667834	1.23	0.2282	-0.0542313	0.2185484	F(1 30)	=	1.51	0.0163
fire carbon emissions	-0.0053221	0.010797	-0.49	0.6257	-0.0273725	0.0167284	F(1 30)	=	0.24	-0.025
GDP	-0.0638232	0.0182249	-3.5	0.0015	-0.1010975	-0.026549	F(1 29)	=	12.26	0.273
anti consum	-0.1228213	0.0613702	-2	0.0545	-0.2481559	0.0025133	F(1 30)	=	4.01	0.0884
coastal city	-1.257858	0.8842786	-1.42	0.1652	-3.063796	0.5480796	F(1 30)	=	2.02	0.032
main clima~l	2.637972	2.082419	1.27	0.2157	-1.62767	6.903614	F(3 28)	=	0.89	-0.0105
main climate snow	0.3617338	2.208739	0.16	0.8711	-4.162663	4.88613				
main climate warm	1.457774	1.891451	0.77	0.4473	-2.416687	5.332236				
soil type Andisols	-1.377261	1.987799	-0.69	0.4953	-5.489336	2.734813	F(8 23)	=	0.49	-0.151
soil type Entisols	-1.122481	1.82591	-0.61	0.5448	-4.899663	2.654701				
soil type Inceptisols	0.6086223	1.551725	0.39	0.6985	-2.601365	3.81861				
soil type Gelisols	-1.417565	2.277309	-0.62	0.5398	-6.128538	3.293409				
soil type Oxisols	-2.047404	2.981698	-0.69	0.4992	-8.215516	4.120708				
soil type Spodosols	-2.087589	2.277309	-0.92	0.3688	-6.798563	2.623384				
soil type Ultisols	-0.3399088	1.648196	-0.21	0.8384	-3.749461	3.069644				
soil type Vertisols	-2.73738	2.981698	-0.92	0.3681	-8.905493	3.430732				
vegetation Forests	1.239849	2.539559	0.49	0.6291	-3.954133	6.433831	F(2 29)	=	1.54	0.0338
vegetation Grasslands	3.444931	2.788151	1.24	0.2265	-2.257478	9.147339				
Europe	0.1570522	0.949964	0.17	0.87	-1.799435	2.11354	F(6 25)	=	4.45	0.4003
the Middle East	-0.7483858	2.10045	-0.36	0.7246	-5.074343	3.577571				
North America	-0.3666175	1.150464	-0.32	0.7526	-2.736042	2.002807				
Oceania	-1.091243	1.575337	-0.69	0.4949	-4.335711	2.153225				
South America	4.586118	1.231498	3.72	0.001	2.049801	7.122434				
Sub Saharan Africa	3.999611	1.575337	2.54	0.0177	0.7551431	7.244078				
**total abundance of AMR gene**	**Coef.**	**Std. Err.**	**t**	**P>t**	**[95% Conf.**	**Interval]**			**F**	**AdjR-squared**
city longitude	-0.6406092	0.4708861	-1.36	0.1838	-1.602287	0.3210685	F(1 30)	=	1.85	0.0267
city latitude	-1.011777	1.362054	-0.74	0.4634	-3.793462	1.769909	F(1 30)	=	0.55	-0.0147
city total population	-0.41909	1.256796	-0.33	0.7413	-2.99352	2.15534	F(1 28)	=	0.11	-0.0316
city population density	-0.0099172	0.00779	-1.27	0.2138	-0.025901	0.0060666	F(1 27)	=	1.62	0.0217
city ave june temp	2.539462	6.005993	0.42	0.6758	-9.783818	14.86274	F(1 27)	=	0.18	-0.0302
pm25	1.842177	2.217116	0.83	0.4126	-2.685777	6.370132	F(1 30)	=	0.69	-0.0101
pm10	-2.574305	4.55714	-0.56	0.5826	-12.50346	7.354851	F(1 12)	=	0.32	-0.0553
o3	-8.74247	3.56817	-2.45	0.0227	-16.1424	-1.342538	F(1 22)	=	6.00	0.1787
no2	-3.638934	3.393758	-1.07	0.3006	-10.87256	3.59469	F(1 15)	=	1.15	0.0093
co	-2.366538	1.388234	-1.7	0.1054	-5.283109	0.5500324	F(1 18)	=	2.91	0.0912
soil ph	1.607916	4.757432	0.34	0.7377	-8.108056	11.32389	F(1 30)	=	0.11	-0.0294
soil organic carbon density	8.737071	5.271695	1.66	0.1079	-2.029166	19.50331	F(1 30)	=	2.75	0.0533
soil organic carbon	0.2759983	0.2527073	1.09	0.2844	-0.2425142	0.7945108	F(1 27)	=	1.19	0.0068
soil total carbon	-0.1150713	0.1540392	-0.75	0.4615	-0.4311336	0.200991	F(1 27)	=	0.56	-0.016
soil total phosphorus	-0.0157335	0.1509259	-0.1	0.9177	-0.3239653	0.2924984	F(1 30)	=	0.01	-0.033
soil moisture(0-10cm)	-17.23495	11.36209	-1.52	0.1405	-40.50914	6.039238	F(1 28)	=	2.30	0.0429
soil moisture(10-40cm)	-5.422376	4.155287	-1.3	0.2025	-13.93409	3.089344	F(1 28)	=	1.70	0.0237
soil tmp	7.069106	5.722282	1.24	0.227	-4.652458	18.79067	F(1 28)	=	1.53	0.0178
precipitation	0.0145469	0.0552817	0.26	0.7942	-0.0983534	0.1274472	F(1 30)	=	0.07	-0.031
SDP	0.3028775	1.116141	0.27	0.788	-1.976587	2.582342	F(1 30)	=	0.07	-0.0308
PCI	-3.428378	4.674592	-0.73	0.469	-12.97517	6.118412	F(1 30)	=	0.54	-0.0151

urban-rural temperature difference day	-0.0786908	37.84976	0	0.9984	-78.37689	78.21951	F(1 23)	=	0.00	-0.0435
urban-rural temperature difference nigh	-9.522687	35.014	-0.27	0.7881	-81.95467	62.90929	F(1 23)	=	0.07	-0.0401
greenness fraction	71.27649	201.1308	0.35	0.7255	-339.4874	482.0403	F(1 30)	=	0.13	-0.029
DHI	148.85	79.57457	1.87	0.0712	-13.66294	311.363	F(1 30)	=	3.50	0.0746
DNI	-25.82553	29.84554	-0.87	0.3937	-86.77825	35.1272	F(1 30)	=	0.75	-0.0082
GHI	25.93773	45.34711	0.57	0.5716	-66.67342	118.5489	F(1 30)	=	0.33	-0.0222
UVindex	15.35358	13.11388	1.17	0.2509	-11.42852	42.13569	F(1 30)	=	1.37	0.0118
wet	0.15488	8.337101	0.02	0.9853	-16.87175	17.18151	F(1 30)	=	0.00	-0.0333
pet	2.049078	38.63896	0.05	0.9581	-76.86221	80.96036	F(1 30)	=	0.00	-0.0332
RH	3.540078	3.544708	1	0.3288	-3.811197	10.89135	F(1 22)	=	1.00	-0.0001
vap	10.22073	5.476526	1.87	0.0718	-0.963827	21.40529	F(1 30)	=	3.48	0.0742
temperature	7.378496	5.54515	1.33	0.1933	-3.94621	18.7032	F(1 30)	=	1.77	0.0243
fire carbon emissions	0.0469044	0.9037446	0.05	0.959	-1.798788	1.892597	F(1 30)	=	0.00	-0.0332
GDP	-2.479509	1.758439	-1.41	0.1692	-6.07592	1.116902	F(1 29)	=	1.99	0.0319
anti consum	-3.263096	5.414597	-0.6	0.5513	-14.32118	7.794987	F(1 30)	=	0.36	-0.021
coastal city	-85.57104	74.54837	-1.15	0.2601	-237.8191	66.67704	F(1 30)	=	1.32	0.0101
main clima~l	281.6191	155.5914	1.81	0.081	-37.09536	600.3336	F(3 28)	=	3.40	0.1883
main climate snow	222.4238	165.0296	1.35	0.1885	-115.6239	560.4715				
main climate warm	32.06029	141.3229	0.23	0.8222	-257.4265	321.5471				
soil type Andisols	-160.0705	150.6876	-1.06	0.2991	-471.7915	151.6506	F(8 23)	=	1.20	0.0484
soil type Entisols	-169.0073	138.4154	-1.22	0.2344	-455.3414	117.3268				
soil type Inceptisols	-144.0237	117.6305	-1.22	0.2332	-387.3609	99.31341				
soil type Gelisols	247.0291	172.6343	1.43	0.1659	-110.0922	604.1505				
soil type Oxisols	-180.2828	226.0314	-0.8	0.4333	-647.8644	287.2988				
soil type Spodosols	-197.6949	172.6343	-1.15	0.2639	-554.8162	159.4265				
soil type Ultisols	-17.23	124.9436	-0.14	0.8915	-275.6954	241.2354				
soil type Vertisols	-211.7093	226.0314	-0.94	0.3587	-679.2909	255.8722				
vegetation Forests	85.71811	200.7336	0.43	0.6725	-324.8281	496.2644	F(2 29)	=	3.35	0.1315
vegetation Grasslands	351.5451	220.3829	1.6	0.1215	-99.18851	802.2787				
Europe	-42.37023	92.8906	-0.46	0.6522	-233.682	148.9415	F(6 25)	=	2.10	0.175
the Middle East	-83.41399	205.3888	-0.41	0.6881	-506.4202	339.5922				
North America	109.6752	112.4961	0.97	0.3389	-122.0148	341.3653				
Oceania	-93.08492	154.0416	-0.6	0.5511	-410.3396	224.1697				
South America	98.87932	120.4199	0.82	0.4193	-149.1301	346.8887				
Sub Saharan Africa	421.5424	154.0416	2.74	0.0113	104.2877	738.7971				

total prevalence of AMR gene	Coef.	Std. Err.	t	P>t	[95% Conf.	Interval]			F	AdjR-squared
city longitude	-0.0867818	0.0338577	-2.56	0.0156	-0.1559284	-0.0176352	F(1 30)	=	6.57	0.1523
city latitude	-0.1844374	0.1004032	-1.84	0.0761	-0.389488	0.0206133	F(1 30)	=	3.37	0.0711
city total population	0.041023	0.0966947	0.42	0.6746	-0.1570472	0.2390932	F(1 28)	=	0.18	-0.0291
city population density	-0.0006065	0.0005593	-1.08	0.2878	-0.0017541	0.0005412	F(1 27)	=	1.18	0.0062
city ave june temp	-0.4511042	0.4204186	-1.07	0.2928	-1.313732	0.4115237	F(1 27)	=	1.15	0.0054
pm25	0.2602398	0.1661093	1.57	0.1277	-0.0790007	0.5994802	F(1 30)	=	2.45	0.0448
pm10	0.1170594	0.17776	0.66	0.5226	-0.2702463	0.5043651	F(1 12)	=	0.43	-0.0455
o3	-0.2710066	0.2826204	-0.96	0.348	-0.8571255	0.3151123	F(1 22)	=	0.92	-0.0035
no2	-0.178634	0.1115312	-1.6	0.1301	-0.416357	0.0590891	F(1 15)	=	2.57	0.0891
co	-0.1642463	0.0927609	-1.77	0.0936	-0.3591297	0.030637	F(1 18)	=	3.14	0.101
soil ph	-0.4023798	0.359808	-1.12	0.2723	-1.137206	0.3324462	F(1 30)	=	1.25	0.008
soil organic carbon density	1.086173	0.3751539	2.9	0.007	0.3200061	1.852339	F(1 30)	=	8.38	0.1923
soil organic carbon	0.0149169	0.0197403	0.76	0.4564	-0.0255869	0.0554207	F(1 27)	=	0.57	-0.0156
soil total carbon	-0.0008482	0.0122432	-0.07	0.9453	-0.0259691	0.0242727	F(1 27)	=	0.00	-0.0369
soil total phosphorus	-0.0090011	0.0115134	-0.78	0.4405	-0.0325146	0.0145125	F(1 30)	=	0.61	-0.0127
soil moisture(0-10cm)	-0.7664653	0.8999636	-0.85	0.4016	-2.609957	1.077027	F(1 28)	=	0.73	-0.0096
soil moisture(10-40cm)	-0.1243684	0.3292217	-0.38	0.7085	-0.7987484	0.5500115	F(1 28)	=	0.14	-0.0305
soil tmp	0.6622134	0.4355511	1.52	0.1396	-0.2299726	1.554399	F(1 28)	=	2.31	0.0433
precipitation	-0.0012125	0.0042583	-0.28	0.7778	-0.0099091	0.0074841	F(1 30)	=	0.08	-0.0305
SDP	-0.0109933	0.0860747	-0.13	0.8992	-0.1867812	0.1647946	F(1 30)	=	0.02	-0.0328
PCI	-0.0740633	0.3631143	-0.2	0.8398	-0.8156417	0.6675151	F(1 30)	=	0.04	-0.0319
urban-rural temperature difference day	1.689565	2.841546	0.59	0.5579	-4.18862	7.567751	F(1 23)	=	0.35	-0.0277
urban-rural temperature difference nigh	-2.733274	2.591095	-1.05	0.3024	-8.093363	2.626814	F(1 23)	=	1.11	0.0047
greenness fraction	17.52122	15.19536	1.15	0.258	-13.51184	48.55428	F(1 30)	=	1.33	0.0105
DHI	14.87874	5.881427	2.53	0.0169	2.867269	26.89022	F(1 30)	=	6.40	0.1483
DNI	0.454346	2.326476	0.2	0.8465	-4.296951	5.205643	F(1 30)	=	0.04	-0.032
GHI	7.225765	3.255609	2.22	0.0342	0.5769242	13.8746	F(1 30)	=	4.93	0.1124
UVindex	2.943379	0.8824238	3.34	0.0023	1.141229	4.745529	F(1 30)	=	11.13	0.2462
wet	-0.3705678	0.6387586	-0.58	0.5662	-1.675087	0.9339513	F(1 30)	=	0.34	-0.0219
pet	1.029005	2.971126	0.35	0.7315	-5.038845	7.096854	F(1 30)	=	0.12	-0.0292
RH	-0.2448595	0.2543952	-0.96	0.3463	-0.7724427	0.2827238	F(1 22)	=	0.93	-0.0032
vap	0.6890174	0.4276386	1.61	0.1176	-0.184337	1.562372	F(1 30)	=	2.60	0.049
temperature	0.6391143	0.4238824	1.51	0.1421	-0.2265691	1.504798	F(1 30)	=	2.27	0.0395
fire carbon emissions	-0.0038421	0.0696282	-0.06	0.9564	-0.1460417	0.1383576	F(1 30)	=	0.00	-0.0332
GDP	-0.3163339	0.1270719	-2.49	0.0188	-0.5762252	-0.0564427	F(1 29)	=	6.20	0.1477
anti consum	-0.9347336	0.3834179	-2.44	0.0209	-1.717777	-0.1516899	F(1 30)	=	5.94	0.1375
coastal city	-8.742033	5.647095	-1.55	0.1321	-20.27494	2.790873	F(1 30)	=	2.40	0.0431
main clima~l	20.24254	12.94297	1.56	0.1291	-6.269926	46.75501	F(3 28)	=	1.59	0.0538
main climate snow	2.094418	13.72809	0.15	0.8798	-26.0263	30.21514				
main climate warm	6.730839	11.75603	0.57	0.5715	-17.3503	30.81198				
soil type Andisols	-14.79722	13.04597	-1.13	0.2684	-41.78487	12.19044	F(8 23)	=	0.35	-0.2016
soil type Entisols	-3.5111	11.98349	-0.29	0.7722	-28.30084	21.27864				
soil type Inceptisols	-5.76825	10.18401	-0.57	0.5766	-26.83548	15.29898				
soil type Gelisols	-12.48059	14.94604	-0.84	0.4123	-43.39883	18.43766				
soil type Oxisols	-15.62957	19.56896	-0.8	0.4326	-56.11105	24.85191				
soil type Spodosols	-15.79894	14.94604	-1.06	0.3015	-46.71718	15.11931				
soil type Ultisols	-8.815485	10.81715	-0.81	0.4235	-31.19247	13.5615				
soil type Vertisols	-17.6016	19.56896	-0.9	0.3777	-58.08308	22.87988				
vegetation Forests	7.346057	16.36367	0.45	0.6568	-26.1214	40.81352	F(2 29)	=	1.44	0.0277
vegetation Grasslands	21.15088	17.96547	1.18	0.2486	-15.59262	57.89439				
Europe	1.036555	5.335082	0.19	0.8475	-9.951251	12.02436	F(6 25)	=	7.10	0.5415
the Middle East	-1.073557	11.79631	-0.09	0.9282	-25.36851	23.2214				
North America	8.494653	6.461105	1.31	0.2005	-4.812241	21.80155				
Oceania	-1.637843	8.847232	-0.19	0.8546	-19.85906	16.58337				
South America	29.80999	6.9162	4.31	0.0002	15.56581	44.05417				
Sub Saharan Africa	39.91356	8.847232	4.51	0.0001	21.69234	58.13478				

Supplementary Table 13. Results of regression analysis, pathogens VS environment and demographic characteristics

Green = p<=0.05, after regression diagnosis

White = p>0.05

total prevenlence	Coef.	Std. Err.	t	P>t	[95% Conf.	Interval]	AdjR-squared
city longie	-0.0021972	0.0012395	-1.77	0.0864	-0.0047286	0.0003341	0.0646
city latitude	-0.0065426	0.0034921	-1.87	0.0708	-0.0136744	0.0005893	0.0749
city total population	0.0071024	0.0031255	2.27	0.0309	0.0007	0.0135048	0.1255
city population density	-0.00000107	0.0000228	-0.05	0.963	-0.0000479	0.0000457	-0.037
city ave june temp	-0.0364826	0.0156375	-2.33	0.0273	-0.0685681	-0.0043971	0.1369
pm25	0.0044761	0.0059656	0.75	0.4589	-0.0077073	0.0166595	-0.0143
pm10	0.0248426	0.0055836	4.45	0.0008	0.012677	0.0370083	0.5911
o3	-0.0047751	0.0052017	-0.92	0.3686	-0.0155627	0.0060125	-0.0069
no2	-0.0043268	0.0027244	-1.59	0.1331	-0.0101338	0.0014801	0.0869
co	-0.0015573	0.0021112	-0.74	0.4703	-0.0059928	0.0028782	-0.0246
soil ph	-0.0085066	0.012704	-0.67	0.5082	-0.0344516	0.0174384	-0.0181
soil organic carbon density	0.0451027	0.0122843	3.67	0.0009	0.0200148	0.0701906	0.287
soil organic carbon	0.000903	0.0006757	1.34	0.1926	-0.0004834	0.0022893	0.0273
soil total carbon	0.0002062	0.0004529	0.46	0.6526	-0.000723	0.0011354	-0.0291
soil total phosphorus	-0.0003344	0.0004007	-0.83	0.4106	-0.0011527	0.0004839	-0.0099
soil moisture(0-10cm)	0.0408678	0.0309472	1.32	0.1973	-0.0225246	0.1042602	0.025
soil moisture(10-40cm)	0.015722	0.0111605	1.41	0.1699	-0.0071393	0.0385833	0.0328
soil tmp	-0.0008479	0.0158564	-0.05	0.9577	-0.0333282	0.0316325	-0.0356
precipitation	-0.0000374	0.0001485	-0.25	0.8031	-0.0003405	0.0002658	-0.0312
SDP	0.0005329	0.0029991	0.18	0.8602	-0.005592	0.0066578	-0.0322
PCI	0.0031905	0.0126504	0.25	0.8026	-0.0226451	0.0290261	-0.0311
urban-rural temperature difference day	-0.0000267	0.1029215	0	0.9998	-0.2129361	0.2128828	-0.0435
urban-rural temperature difference night	-0.1328481	0.0912516	-1.46	0.159	-0.3216165	0.0559203	0.0446
greenfraction	0.4855118	0.5338797	0.91	0.3704	-0.6048161	1.57584	-0.0056
DHI	0.1735059	0.2235505	0.78	0.4437	-0.2830451	0.630057	-0.013
DNI	0.0451683	0.0807124	0.56	0.5799	-0.1196684	0.2100051	-0.0227
GHI	0.14863	0.119379	1.25	0.2228	-0.0951746	0.3924345	0.0174
UVindex	0.0872512	0.0322924	2.7	0.0112	0.0213013	0.153201	0.1689
wet	-0.0044634	0.0223714	-0.2	0.8432	-0.0501518	0.041225	-0.032
pet	-0.0748369	0.1028513	-0.73	0.4725	-0.2848874	0.1352135	-0.0154
RH	0.0008678	0.0047681	0.18	0.8572	-0.0090207	0.0107563	-0.0439
vap	-0.0023797	0.0155292	-0.15	0.8792	-0.0340946	0.0293352	-0.0325
temperature	-0.0049671	0.0152956	-0.32	0.7476	-0.0362048	0.0262707	-0.0297
fire carbon emissions	-0.0009835	0.0024201	-0.41	0.6874	-0.005926	0.0039591	-0.0277
GDP	-0.0087072	0.0046008	-1.89	0.0684	-0.0181168	0.0007025	0.0792
anti consum	0.0008266	0.0146258	0.06	0.9553	-0.0290432	0.0306964	-0.0332
coastal city	-0.3021815	0.1969375	-1.53	0.1354	-0.7043816	0.1000186	0.0419
main clima~l	0.1651143	0.4787241	0.34	0.7327	-0.8155076	1.145736	-0.0657
main climate snow	0.0385825	0.5077636	0.08	0.94	-1.001524	1.078689	
main climate warm	0.2999823	0.4348228	0.69	0.4959	-0.5907118	1.190676	
soil type Andisols	0.0093024	0.4454689	0.02	0.9835	-0.9122202	0.9308251	-0.1535
soil type Entisols	0.4527061	0.4091893	1.11	0.28	-0.3937665	1.299179	
soil type Inceptisols	0.4628735	0.3477441	1.33	0.1962	-0.2564899	1.182237	
soil type Gelisols	0.0113742	0.5103488	0.02	0.9824	-1.044363	1.067111	
soil type Oxisols	-0.032531	0.6682034	-0.05	0.9616	-1.414815	1.349753	
soil type Spodosols	-0.0113992	0.5103488	-0.02	0.9824	-1.067136	1.044338	
soil type Ultisols	0.1287309	0.3693633	0.35	0.7306	-0.6353554	0.8928171	
soil type Vertisols	-0.083813	0.6682034	-0.13	0.9013	-1.466097	1.298471	
vegetation Forests	0.2762507	0.5916933	0.47	0.6441	-0.933898	1.486399	-0.0466
vegetation Grasslands	0.0695255	0.6496127	0.11	0.9155	-1.259082	1.398133	
Europe	0.0344964	0.2216705	0.16	0.8776	-0.4220427	0.4910354	0.3483
the Middle East	-0.0879507	0.490132	-0.18	0.859	-1.097396	0.9214951	
North America	-0.0082923	0.2684564	-0.03	0.9756	-0.5611885	0.544604	
Oceania	-0.023665	0.367599	-0.06	0.9492	-0.7807493	0.7334194	
South America	1.170332	0.2873654	4.07	0.0004	0.5784919	1.762172	
Sub Saharan Africa	0.0192009	0.367599	0.05	0.9588	-0.7378835	0.7762852	
**abundance of Brucella**	**Coef.**	**Std. Err.**	**t**	**P>t**	**[95% Conf.**	**Interval]**	**AdjR-squared**
city longitude	-0.00000073	0.00000308	-0.24	0.8146	-0.00000703	0.00000557	-0.0314
city latitude	0.00000833	0.00000861	0.97	0.3413	-0.00000926	0.0000259	-0.0021
city total population	0.0000133	0.00000768	1.73	0.0954	-0.00000248	0.000029	0.0639
city population density	-3.94E-09	5.42E-08	-0.07	0.9426	-0.000000115	0.000000107	-0.0368
city ave june temp	-0.0000512	0.0000395	-1.3	0.2058	-0.0001323	0.0000299	0.0237
pm25	-0.00000506	0.0000142	-0.36	0.7247	-0.0000341	0.000024	-0.029
pm10	0.000000324	0.000000191	1.7	0.1153	-9.18E-08	0.000000739	0.1265
o3	0.000017	0.0000453	0.37	0.7121	-0.0000771	0.000111	-0.0389
no2	-0.0000385	0.0000293	-1.31	0.2094	-0.000101	0.000024	0.0431
co	0.00000941	0.000019	0.5	0.6265	-0.0000305	0.0000493	-0.0414
soil ph	0.0000474	0.0000291	1.63	0.1133	-0.0000119	0.0001068	0.0509
soil organic carbon density	0.0000227	0.0000348	0.65	0.5197	-0.0000484	0.0000937	-0.0189
soil organic carbon	-0.000000396	0.00000166	-0.24	0.8135	-0.00000381	0.00000302	-0.0349
soil total carbon	1.73E-08	0.00000108	0.02	0.9874	-0.00000221	0.00000224	-0.037
soil total phosphorus	0.00000105	0.000000941	1.11	0.2741	-0.000000874	0.00000297	0.0077
soil moisture(0-10cm)	0.0000586	0.0000751	0.78	0.442	-0.0000952	0.0002124	-0.0137
soil moisture(10-40cm)	0.0000226	0.0000271	0.83	0.4129	-0.000033	0.0000782	-0.0108
soil tmp	-0.0000333	0.0000372	-0.89	0.3788	-0.0001095	0.0000429	-0.007
precipitation	-0.000000178	0.000000351	-0.51	0.6156	-0.000000894	0.000000538	-0.0245
SDP	-0.00000322	0.00000709	-0.45	0.6531	-0.0000177	0.0000113	-0.0263
PCI	-0.00000602	0.00003	-0.2	0.8422	-0.0000673	0.0000552	-0.0319
urban-rural temperature difference day	0.0000176	0.0002464	0.07	0.9435	-0.000492	0.0005273	-0.0432
urban-rural temperature difference night	0.0000343	0.0002282	0.15	0.8818	-0.0004377	0.0005063	-0.0425
greenfraction	0.0005843	0.0012779	0.46	0.6508	-0.0020255	0.0031942	-0.0262
DHI	-0.0003473	0.0005312	-0.65	0.5182	-0.0014322	0.0007376	-0.0188
DNI	-0.0002057	0.0001885	-1.09	0.284	-0.0005908	0.0001794	0.0061
GHI	-0.0004638	0.0002775	-1.67	0.105	-0.0010304	0.0001029	0.0547
UVindex	-0.0000986	0.0000834	-1.18	0.2463	-0.0002689	0.0000717	0.0127
wet	-0.00000355	0.000053	-0.07	0.9472	-0.0001119	0.0001048	-0.0332
pet	-0.0003285	0.0002384	-1.38	0.1785	-0.0008154	0.0001584	0.0282
RH	-0.00000988	0.0000409	-0.24	0.8114	-0.0000947	0.0000749	-0.0427
vap	-0.0000252	0.0000365	-0.69	0.4962	-0.0000998	0.0000494	-0.0172
temperature	-0.0000332	0.0000358	-0.93	0.361	-0.0001063	0.0000399	-0.0045
fire carbon emissions	-0.00000164	0.00000574	-0.29	0.7768	-0.0000134	0.0000101	-0.0305
GDP	0.00000344	0.0000115	0.3	0.7678	-0.0000202	0.0000271	-0.0313
anti consum	0.00000676	0.0000346	0.2	0.8466	-0.000064	0.0000775	-0.032
coastal city	-0.0004503	0.0004776	-0.94	0.3533	-0.0014256	0.0005251	-0.0036
main clima~l	0.00000604	0.0011454	0.01	0.9958	-0.0023402	0.0023523	-0.0866
main climate snow	0.0000138	0.0012149	0.01	0.991	-0.0024748	0.0025024	
main climate warm	0.0003804	0.0010404	0.37	0.7174	-0.0017507	0.0025115	
soil type Andisols	0.00000372	0.0010849	0	0.9973	-0.0022406	0.0022481	-0.2186
soil type Entisols	0.00000718	0.0009966	0.01	0.9943	-0.0020544	0.0020688	
soil type Inceptisols	0.000943	0.0008469	1.11	0.277	-0.000809	0.002695	
soil type Gelisols	-0.0000147	0.001243	-0.01	0.9906	-0.002586	0.0025565	
soil type Oxisols	-0.0000147	0.0016274	-0.01	0.9929	-0.0033813	0.0033518	
soil type Spodosols	-0.0000147	0.001243	-0.01	0.9906	-0.002586	0.0025565	
soil type Ultisols	-0.00000796	0.0008996	-0.01	0.993	-0.0018689	0.001853	
soil type Vertisols	-0.0000147	0.0016274	-0.01	0.9929	-0.0033813	0.0033518	
vegetation Forests	0.0002763	0.001413	0.2	0.8463	-0.0026136	0.0031662	-0.063
vegetation Grasslands	0.00000466	0.0015513	0	0.9976	-0.0031681	0.0031774	
Europe	0.0006884	0.0007021	0.98	0.3363	-0.0007577	0.0021344	-0.1644
the Middle East	0.0000028	0.0015524	0	0.9986	-0.0031945	0.0032001	
North America	-0.00000263	0.0008503	0	0.9976	-0.0017539	0.0017486	
Oceania	-0.000011	0.0011643	-0.01	0.9926	-0.0024089	0.002387	
South America	-0.00000576	0.0009102	-0.01	0.995	-0.0018803	0.0018688	
Sub Saharan Africa	0.0000259	0.0011643	0.02	0.9824	-0.0023721	0.0024239	
**abundance of Salmonella enterica**	**Coef.**	**Std. Err.**	**t**	**P>t**	**[95% Conf.**	**Interval]**	**AdjR-squared**

city longitude	-0.00000175	0.00000171	-1.02	0.3139	-0.00000524	0.00000174	0.0016
city latitude	0.00000182	0.00000491	0.37	0.714	-0.00000821	0.0000118	-0.0286
city total population	-0.000000854	0.00000447	-0.19	0.85	-0.00001	0.00000831	-0.0344
city population density	-1.88E-08	2.97E-08	-0.63	0.5318	-7.97E-08	4.21E-08	-0.0219
city ave june temp	-0.000014	0.0000223	-0.63	0.5369	-0.0000597	0.0000318	-0.0222
pm25	-0.00000923	0.00000785	-1.18	0.249	-0.0000253	0.0000068	0.0122
pm10	-0.000000749	0.0000155	-0.05	0.9624	-0.0000346	0.0000331	-0.0831
o3	-0.0000109	0.0000201	-0.55	0.5906	-0.0000525	0.0000306	-0.0315
no2	-0.0000193	0.0000125	-1.54	0.1434	-0.0000459	0.00000734	0.0796
co	-0.00000406	0.00000624	-0.65	0.5235	-0.0000172	0.00000905	-0.0313
soil ph	0.0000197	0.0000167	1.18	0.2466	-0.0000144	0.0000538	0.0126
soil organic carbon density	0.0000315	0.0000189	1.67	0.105	-0.00000699	0.00007	0.0547
soil organic carbon	0.000000127	0.000000924	0.14	0.8918	-0.00000177	0.00000202	-0.0363
soil total carbon	-0.000000377	0.000000598	-0.63	0.533	-0.0000016	0.000000849	-0.0219
soil total phosphorus	0.000000673	0.000000526	1.28	0.2109	-0.000000402	0.00000175	0.0201
soil moisture(0-10cm)	0.0000227	0.0000421	0.54	0.5949	-0.0000636	0.0001089	-0.0251
soil moisture(10-40cm)	0.000000377	0.0000153	0.02	0.9806	-0.000031	0.0000318	-0.0357
soil tmp	-0.0000241	0.0000205	-1.18	0.2497	-0.0000662	0.0000179	0.013
precipitation	-6.26E-08	0.000000198	-0.32	0.7538	-0.000000467	0.000000341	-0.0299
SDP	1.31E-08	0.000004	0	0.9974	-0.00000816	0.00000818	-0.0333
PCI	-0.00000565	0.0000169	-0.34	0.7398	-0.0000401	0.0000288	-0.0295
urban-rural temperature difference day	0.0000986	0.0001154	0.85	0.4016	-0.0001401	0.0003373	-0.0114
urban-rural temperature difference night	0.0001071	0.0001063	1.01	0.324	-0.0001127	0.0003269	0.0007
greenfraction	0.0006017	0.000713	0.84	0.4055	-0.0008545	0.0020579	-0.0094
DHI	-0.0002804	0.0002966	-0.95	0.3521	-0.0008861	0.0003254	-0.0034
DNI	0.0000256	0.0001081	0.24	0.8146	-0.0001951	0.0002462	-0.0314
GHI	-0.0001097	0.000162	-0.68	0.5035	-0.0004405	0.0002211	-0.0178
UVindex	-0.0000239	0.0000478	-0.5	0.6213	-0.0001215	0.0000738	-0.0248
wet	0.0000024	0.0000298	0.08	0.9365	-0.0000585	0.0000633	-0.0331
pet	-0.0001214	0.0001365	-0.89	0.3811	-0.0004002	0.0001575	-0.0068
RH	-0.000000496	0.0000182	-0.03	0.9785	-0.0000382	0.0000372	-0.0454
vap	-0.000023	0.0000203	-1.14	0.2651	-0.0000644	0.0000184	0.0093
temperature	-0.0000297	0.0000197	-1.51	0.1424	-0.0000699	0.0000106	0.0393
fire carbon emissions	-0.00000243	0.0000032	-0.76	0.4537	-0.00000898	0.00000411	-0.0139
GDP	0.00000631	0.00000637	0.99	0.3303	-0.00000672	0.0000193	-0.0007
anti consum	-0.0000281	0.0000188	-1.49	0.1463	-0.0000665	0.0000104	0.038
coastal city	-0.0005956	0.00025	-2.38	0.0238	-0.0011062	-0.000085	0.131
main clima~l	-0.0005403	0.0006337	-0.85	0.4011	-0.0018384	0.0007578	-0.0508
main climate snow	-0.0000927	0.0006721	-0.14	0.8913	-0.0014695	0.0012841	
main climate warm	-0.0004666	0.0005756	-0.81	0.4245	-0.0016456	0.0007125	
soil type Andisols	0.00000893	0.0004817	0.02	0.9854	-0.0009875	0.0010053	0.2411
soil type Entisols	0.0001418	0.0004424	0.32	0.7515	-0.0007735	0.0010571	
soil type Inceptisols	0.0006127	0.000376	1.63	0.1168	-0.0001651	0.0013906	
soil type Gelisols	0.0020586	0.0005518	3.73	0.0011	0.0009171	0.0032001	
soil type Oxisols	-0.00000411	0.0007225	-0.01	0.9955	-0.0014987	0.0014905	
soil type Spodosols	0.0005193	0.0005518	0.94	0.3564	-0.0006222	0.0016609	
soil type Ultisols	0.0003415	0.0003994	0.86	0.4013	-0.0004847	0.0011677	
soil type Vertisols	-0.00000411	0.0007225	-0.01	0.9955	-0.0014987	0.0014905	
vegetation Forests	0.0004153	0.0007934	0.52	0.6047	-0.0012074	0.002038	-0.059
vegetation Grasslands	0.0004027	0.0008711	0.46	0.6473	-0.0013789	0.0021843	
Europe	0.0001207	0.0003872	0.31	0.7578	-0.0006766	0.0009181	-0.1186
the Middle East	-0.0002735	0.000856	-0.32	0.752	-0.0020365	0.0014895	
North America	0.0005893	0.0004689	1.26	0.2204	-0.0003764	0.0015549	
Oceania	0.00026	0.000642	0.4	0.6889	-0.0010623	0.0015823	
South America	0.0000244	0.0005019	0.05	0.9617	-0.0010093	0.001058	
Sub Saharan Africa	-0.0002733	0.000642	-0.43	0.674	-0.0015956	0.001049	

total abundance of pathogens	Coef.	Std. Err.	t	P>t	[95% Conf.	Interval]	AdjR-squared
city longitude	-0.0000033	0.00000729	-0.45	0.6542	-0.0000182	0.0000116	-0.0263
city latitude	0.0000133	0.0000206	0.65	0.5217	-0.0000287	0.0000554	-0.0191
city total population	0.0000241	0.0000185	1.3	0.2028	-0.0000138	0.0000621	0.0236
city population density	-5.82E-08	0.000000127	-0.46	0.6503	-0.000000319	0.000000202	-0.029
city ave june temp	-0.0001238	0.0000928	-1.33	0.1932	-0.0003142	0.0000666	0.0271
pm25	-0.000013	0.0000337	-0.39	0.7015	-0.0000819	0.0000558	-0.0282
pm10	0.0000352	0.0000385	0.92	0.3781	-0.0000486	0.000119	-0.0127
o3	0.0000375	0.0001007	0.37	0.7134	-0.0001713	0.0002462	-0.0389
no2	-0.0001238	0.0000619	-2	0.064	-0.0002558	0.00000816	0.1578
co	0.0000239	0.000042	0.57	0.5757	-0.0000643	0.0001122	-0.0368
soil ph	0.0001173	0.0000686	1.71	0.0976	-0.0000228	0.0002573	0.0585
soil organic carbon density	0.000136	0.0000792	1.72	0.0964	-0.0000258	0.0002977	0.0591
soil organic carbon	0.00000156	0.00000389	0.4	0.6916	-0.00000642	0.00000954	-0.0309
soil total carbon	-0.000000926	0.00000256	-0.36	0.7198	-0.00000617	0.00000432	-0.032
soil total phosphorus	0.000000804	0.00000227	0.35	0.7256	-0.00000383	0.00000544	-0.029
soil moisture(0-10cm)	0.0002957	0.0001702	1.74	0.0933	-0.0000529	0.0006442	0.0651
soil moisture(10-40cm)	0.0000984	0.0000621	1.58	0.1243	-0.0000288	0.0002257	0.0495
soil tmp	-0.0000994	0.000087	-1.14	0.2633	-0.0002777	0.0000789	0.0104
precipitation	-0.000000319	0.000000832	-0.38	0.7044	-0.00000202	0.00000138	-0.0283
SDP	-0.000000273	0.0000168	-0.02	0.9872	-0.0000347	0.0000341	-0.0333
PCI	0.0000245	0.0000709	0.35	0.7324	-0.0001204	0.0001694	-0.0292
urban-rural temperature difference day	-0.0003048	0.0005728	-0.53	0.5998	-0.0014898	0.0008802	-0.0308
urban-rural temperature difference night	0.0002288	0.0005319	0.43	0.6711	-0.0008715	0.0013291	-0.0352
greenfraction	-0.0008961	0.0030333	-0.3	0.7697	-0.0070909	0.0052987	-0.0303
DHI	-0.0009539	0.0012553	-0.76	0.4533	-0.0035175	0.0016098	-0.0138
DNI	-0.0002263	0.0004535	-0.5	0.6214	-0.0011525	0.0006999	-0.0248
GHI	-0.0007725	0.0006725	-1.15	0.2598	-0.002146	0.000601	0.0102
UVindex	-0.0001546	0.0002001	-0.77	0.4459	-0.0005633	0.0002541	-0.0132
wet	0.0000189	0.0001256	0.15	0.8812	-0.0002376	0.0002755	-0.0326
pet	-0.0006858	0.0005688	-1.21	0.2373	-0.0018474	0.0004757	0.0144
RH	-0.0000393	0.0000905	-0.43	0.6683	-0.0002271	0.0001484	-0.0366
vap	-0.0000818	0.0000859	-0.95	0.3487	-0.0002572	0.0000937	-0.003
temperature	-0.000106	0.0000838	-1.27	0.2154	-0.0002772	0.0000651	0.019
fire carbon emissions	0.0000127	0.0000134	0.95	0.3517	-0.0000147	0.0000401	-0.0034
GDP	0.0000185	0.0000271	0.68	0.5005	-0.0000369	0.0000739	-0.0181
anti consum	-0.0000676	0.0000812	-0.83	0.4115	-0.0002334	0.0000982	-0.01
coastal city	-0.0015363	0.0011132	-1.38	0.1777	-0.0038097	0.000737	0.0284
main clima~l	-0.0007736	0.0027122	-0.29	0.7776	-0.0063294	0.0047821	-0.0858
main climate snow	0.0003257	0.0028767	0.11	0.9107	-0.0055671	0.0062184	
main climate warm	0.0003539	0.0024635	0.14	0.8868	-0.0046924	0.0054001	
soil type Andisols	0.000639	0.0024451	0.26	0.7962	-0.0044191	0.0056971	-0.103
soil type Entisols	0.0029092	0.002246	1.3	0.2081	-0.001737	0.0075553	
soil type Inceptisols	0.0028298	0.0019087	1.48	0.1518	-0.0011187	0.0067783	
soil type Gelisols	0.0039091	0.0028012	1.4	0.1762	-0.0018857	0.0097039	
soil type Oxisols	-0.0001803	0.0036677	-0.05	0.9612	-0.0077674	0.0074068	
soil type Spodosols	0.000577	0.0028012	0.21	0.8386	-0.0052178	0.0063718	
soil type Ultisols	0.0008586	0.0020274	0.42	0.6759	-0.0033353	0.0050526	
soil type Vertisols	-0.0002769	0.0036677	-0.08	0.9405	-0.007864	0.0073103	
vegetation Forests	0.0001534	0.0033333	0.05	0.9636	-0.006664	0.0069707	-0.0543
vegetation Grasslands	-0.0009618	0.0036596	-0.26	0.7946	-0.0084465	0.006523	
Europe	0.0001578	0.0016933	0.09	0.9265	-0.0033297	0.0036453	-0.207
the Middle East	-0.000109	0.0037441	-0.03	0.977	-0.0078201	0.0076021	
North America	0.0002854	0.0020507	0.14	0.8904	-0.0039382	0.0045089	
Oceania	-0.0012002	0.0028081	-0.43	0.6727	-0.0069835	0.0045832	
South America	-0.000169	0.0021952	-0.08	0.9392	-0.0046901	0.004352	
Sub Saharan Africa	-0.0015849	0.0028081	-0.56	0.5775	-0.0073682	0.0041984	

Codes used in this study

```
#Alpha diversity
library(vegan)
library(ggplot2)
library(ggpubr)
otu <- read.csv('otu.csv', header=T, row.names=1)
observed_species <- estimateR(otu)[1, ]
Shannon <- diversity(otu, index = 'shannon',base = 2)
result <- data.frame(observed_species, Shannon)

# Boxplot
library(ggplot2)
library(ggpubr)
dat <- read.table("index.txt", header=T)
p<-ggplot(dat,aes(x=continent,y=fisher_alpha,fill=continent))+
  geom_boxplot(show.legend = F,width = 0.5, lwd = 0.6)+
  stat_boxplot(geom = "errorbar", width = 0.2, lwd = 0.6,show.legend = F)+
  theme_bw()+
  theme(panel.grid = element_blank())+
  stat_compare_means(aes(label = ..p.signif..))+
  xlab("")

#Beta diversity
library(vegan)
library(ggplot2)
library(ggrepel)
otu<-read.table("taxonomy.txt",header=T,row.names = 1)
otu<-as.data.frame(otu)
bray < vegdist(otu,method = "bray")
bray <- as.matrix(bray)
dis <- as.dist(bray)
group <- read.table('group.txt',header = T, row.names = 1, sep = '\t', stringsAsFactors = FALSE)
adonis_result_dis <- adonis2(dis~city,group, permutations = 999,na.action=na.omit) \
adonis_result_dis
for (i in c(54:62)){
  adonis_result<- adonis2(dis~ group[,i],group,permutations = 999,na.action=na.omit)
  write.csv(adonis_result,file=paste("",colnames(group[i]),".csv"))
}

#Bar plot
library(reshape2)
library(ggplot2)
species_top10 <- read.table('abun.txt', row.names = 1, header=T)
```

```
p <- ggplot(species_top10, aes(variable, 100*value, fill = Taxonomy)) +
  geom_col(position = 'stack', width = 0.85) +
  labs(x = '', y = 'Relative Abundancce(%)') +
  theme(panel.grid = element_blank(), panel.background = element_rect(color = 'black',fill = 'transparent'), strip.text = element_text(size = 12),strip.background = element_blank()) +
  theme(axis.text.x = element_text(angle = 60,hjust = 1))+
  theme(axis.text = element_text(size = 10), axis.title = element_text(size = 13), legend.title = element_blank(), legend.text = element_text(size = 11))
p

#scatter point
library(ggplot2)
library(ggpubr)
dat<-read.csv("regress.csv",header=T)
p<-ggplot(dat,aes(x=env,y=variable))+
  geom_point()+stat_smooth(method = lm,colour='#FDB462',se = FALSE,size=0.7)+
  theme_bw() +
  theme(panel.grid.major=element_line(colour=NA),panel.grid.minor = element_blank())
p

#CCA
library(vegan)
library(ggplot2)
otu<-read.table("cca_taxonomy.txt",header=T,row.names = 1)
group <- read.table('cca_group.txt',header = T, row.names = 1, sep = '\t', stringsAsFactors = FALSE)
print(rownames(otu)==rownames(group))
decorana(otu)
otu_cca<-cca(otu,group)
otu_cca.scaling1 <- summary(otu_cca, scaling = 1)
otu_cca.scaling1
otu_cca_test <- anova.cca(out.cca, permutations = 999)
otu_cca_test
otu_cca.site <- data.frame(otu_cca.scaling1$sites)[1:2]
otu_cca.env <- data.frame(otu_cca.scaling1$biplot)[1:2]
r2 <- RsquareAdj(res)
otu_cca_noadj <- r2$r.squared
otu_cca_adj <- r2$adj.r.squared
p <- ggplot(otu_cca.site, aes(CCA1, CCA2)) +
  geom_point(aes(color = group)) +
  scale_color_manual(values = c('#D92546','#FDB462','#FCCDE5','#538B11','#BC80BD',"#4693E2","#ABDEC4")) +
  theme(panel.grid = element_blank(), panel.background = element_rect(color = 'black', fill = 'transparent'), plot.title = element_text(hjust = 0.5), legend.key = element_rect(fill = 'transparent')) +
```

```
  geom_segment(data = otu_cca.env,aes(x=0, xend= CCA1*2.5, y=0, yend= CCA2*2.5 ), arrow = arrow(length = unit(0.2, "cm")), colour = 'blue') +
  geom_vline(xintercept = 0, color = 'gray', size = 0.5) +
  geom_hline(yintercept = 0, color = 'gray', size = 0.5) +
  geom_text(data = otu_cca.env, aes(CCA1 * 2.9, CCA2 * 2.9, label = name), color = 'blue', size = 3)
p
```
