# Supplementary material for: Environmental determinants and demographic influences on global urban microbiomes, antimicrobial resistance and pathogenicity
Source: NPJ Biofilms Microbiomes. 2023 Dec 7;9:94. doi: 10.1038/s41522-023-00459-4 (PMC10703778; doi:10.1038/s41522-023-00459-4)
Supplement: Supplementary file 2 — Reporting Summary [file 41522_2023_459_MOESM2_ESM.pdf]

nature portfolio

Corresponding author(s): Yu Sun, Yiqun Deng, Xi Fu

Last updated by author(s): Nov 15, 2023

Reporting Summary

Nature Portfolio wishes to improve the reproducibility of the work that we publish. This form provides structure for consistency and transparency in reporting. For further information on Nature Portfolio policies, see our Editorial Policies and the Editorial Policy Checklist.

Statistics

For all statistical analyses, confirm that the following items are present in the figure legend, table legend, main text, or Methods section.

n/a	Confirmed	
☐	☒	The exact sample size (*n*) for each experimental group/condition, given as a discrete number and unit of measurement
☒	☐	A statement on whether measurements were taken from distinct samples or whether the same sample was measured repeatedly
☐	☒	The statistical test(s) used AND whether they are one- or two-sided<br>*Only common tests should be described solely by name; describe more complex techniques in the Methods section.*
☐	☒	A description of all covariates tested
☒	☐	A description of any assumptions or corrections, such as tests of normality and adjustment for multiple comparisons
☐	☒	A full description of the statistical parameters including central tendency (e.g. means) or other basic estimates (e.g. regression coefficient) AND variation (e.g. standard deviation) or associated estimates of uncertainty (e.g. confidence intervals)
☐	☒	For null hypothesis testing, the test statistic (e.g. *F*, *t*, *r*) with confidence intervals, effect sizes, degrees of freedom and *P* value noted<br>*Give P values as exact values whenever suitable.*
☒	☐	For Bayesian analysis, information on the choice of priors and Markov chain Monte Carlo settings
☒	☐	For hierarchical and complex designs, identification of the appropriate level for tests and full reporting of outcomes
☐	☒	Estimates of effect sizes (e.g. Cohen's *d*, Pearson's *r*), indicating how they were calculated

Our web collection on statistics for biologists contains articles on many of the points above.

Software and code

Policy information about availability of computer code

Data collection	Not Applicable
Data analysis	Not Applicable

For manuscripts utilizing custom algorithms or software that are central to the research but not yet described in published literature, software must be made available to editors and reviewers. We strongly encourage code deposition in a community repository (e.g. GitHub). See the Nature Portfolio guidelines for submitting code & software for further information.

Data

Policy information about availability of data

All manuscripts must include a data availability statement. This statement should provide the following information, where applicable:

- Accession codes, unique identifiers, or web links for publicly available datasets
- A description of any restrictions on data availability
- For clinical datasets or third party data, please ensure that the statement adheres to our policy

The authors confirm that the data supporting the findings of this study are available within its supplementary materials.

Research involving human participants, their data, or biological material

Policy information about studies with human participants or human data. See also policy information about sex, gender (identity/presentation), and sexual orientation and race, ethnicity and racism.

Reporting on sex and gender	Not Applicable
Reporting on race, ethnicity, or other socially relevant groupings	Not Applicable
Population characteristics	Not Applicable
Recruitment	Not Applicable
Ethics oversight	Not Applicable

Note that full information on the approval of the study protocol must also be provided in the manuscript.

Field-specific reporting

Please select the one below that is the best fit for your research. If you are not sure, read the appropriate sections before making your selection.

☒ Life sciences ☐ Behavioural & social sciences ☐ Ecological, evolutionary & environmental sciences

For a reference copy of the document with all sections, see nature.com/documents/nr-reporting-summary-flat.pdf

Life sciences study design

All studies must disclose on these points even when the disclosure is negative.

Sample size	2,674 environmental samples in total. Upon this screening, we observed extensive variation in sampling intensity among cities in the MetaSUB project, ranging from 1 to 548 samples. Consequently, cities with fewer than 14 samples from transportation systems were removed, resulting in a total of 32 cities included for subsequent analysis.
Data exclusions	Predominantly, samples (numbering 2,674) were obtained from high-contact surfaces in metro and transit stations, including ticket kiosks, turnstiles, railings, and seats or benches. Lesser samples were collected from residences (381), seashores (178), schools (142), hospitals (45), parks (52), and other urban locations. To maintain sample source consistency, only samples from transportation systems were retained for further analysis.
Replication	We provided comprehensive raw data, as well as detailed descriptions of the methods and software used, etc
Randomization	Given the disparities in the number of samples collected per city, we standardized the sample size to 55 for each city to ensure a balanced comparative analysis. This was achieved using a drawing without replacement statistical technique. The number 55 was chosen because half of the cities had collected 55 or more samples from transportation systems. This selection process was iterated until the alpha diversity distribution of each city's subsample was not significantly different from alpha diversity distribution of the total sample distribution, as determined by a Two Sample Kolmogorov-Smirnov test. This strategy allowed us to control for sampling bias and to ensure the robustness of our conclusions to differences in sample size across cities.
Blinding	We performed a secondary analysis of the MetaSuB data. no blinding

Reporting for specific materials, systems and methods

We require information from authors about some types of materials, experimental systems and methods used in many studies. Here, indicate whether each material, system or method listed is relevant to your study. If you are not sure if a list item applies to your research, read the appropriate section before selecting a response.

Materials & experimental systems

n/a	Involved in the study
☒	☐ Antibodies
☒	☐ Eukaryotic cell lines
☒	☐ Palaeontology and archaeology
☒	☐ Animals and other organisms
☒	☐ Clinical data
☒	☐ Dual use research of concern
☒	☐ Plants

Methods

n/a	Involved in the study
☒	☐ ChIP-seq
☒	☐ Flow cytometry
☒	☐ MRI-based neuroimaging
